# High-throughput identification of peptide agonists against GPCRs by co-culture of mammalian reporter cells and peptide-secreting yeast cells using droplet microfluidics

**DOI:** 10.1038/s41598-019-47388-x

**Published:** 2019-07-29

**Authors:** Kenshi Yaginuma, Wataru Aoki, Natsuko Miura, Yuta Ohtani, Shunsuke Aburaya, Masato Kogawa, Yohei Nishikawa, Masahito Hosokawa, Haruko Takeyama, Mitsuyoshi Ueda

**Affiliations:** 1 Division of Applied Life Sciences, Graduate School of Agriculture, Kyoto University, Sakyo-ku, Kyoto 606-8502 Japan; 2 JST, CREST, 7 Goban-cho, Chiyoda-ku, Tokyo 102-0076 Japan; 3 JST, PRESTO, 7 Goban-cho, Chiyoda-ku, Tokyo 102-0076 Japan; 4 Graduate School of Life and Environmental Sciences, Osaka Prefecture University, 1-1 Gakuen-cho, Naka-ku, Sakai, Osaka 599-8531 Japan; 5 Japan Society for the Promotion of Science, 5-3-1 Kojimachi, Chiyoda-ku, Tokyo 102-0083 Japan; 6 Department of Life Science & Medical Bioscience, School of Advanced Science and Engineering, Waseda University, Shinjuku-ku, Tokyo 169-8555 Japan; 7 Computational Bio Big-Data Open Innovation Laboratory, AIST-Waseda University, Shinjuku-ku, Tokyo 169–0072 Japan; 8 Institute for Advanced Research of Biosystem Dynamics, Waseda Research Institute for Science and Engineering, Waseda University, Shinjuku-ku, Tokyo 169-8555 Japan

**Keywords:** Assay systems, High-throughput screening

## Abstract

Since G-protein coupled receptors (GPCRs) are linked to various diseases, screening of functional ligands against GPCRs is vital for drug discovery. In the present study, we developed a high-throughput functional cell-based assay by combining human culture cells producing a GPCR, yeast cells secreting randomized peptide ligands, and a droplet microfluidic device. We constructed a reporter human cell line that emits fluorescence in response to the activation of human glucagon-like peptide-1 receptor (hGLP1R). We then constructed a yeast library secreting an agonist of hGLP1R or randomized peptide ligands. We demonstrated that high-throughput identification of functional ligands against hGLP1R could be performed by co-culturing the reporter cells and the yeast cells in droplets. We identified functional ligands, one of which had higher activity than that of an original sequence. The result suggests that our system could facilitate the discovery of functional peptide ligands of GPCRs.

## Introduction

G-protein coupled receptors (GPCRs) are seven-transmembrane receptors representing the largest membrane protein family in humans^1^. GPCRs play key roles in transmitting extracellular signals as intracellular responses^1^. Natural ligands for GPCRs are diverse. Most neurotransmitters^2,3^, autacoids^4,5^, and hormones^6,7^ act by binding to GPCRs. Since abnormal activities of GPCRs are linked to various human diseases, screening of drugs targeting GPCRs has become an important field of study. Indeed, approximately 50% of the drugs in the market act on signal transduction systems involving GPCRs, 30% of which act on GPCRs directly^8,9^. Based on the currently clarified functions of GPCRs, about 400 GPCRs are druggable^8–11^. However, drugs targeting GPCRs have been developed only for approximately 100 GPCRs^10^. Approximately 60 GPCRs among the 400 druggable GPCRs reportedly receive peptides as ligands^10^. Some of the GPCRs are associated with diseases such as type 2 diabetes, cancer, asthma, rheumatoid arthritis, chronic obstructive pulmonary disease (COPD), HIV, epilepsy, cardiovascular disorders, or migraine^10^. Therefore, an approach that can identify drug candidates for peptide-activated GPCRs in a high-throughput manner would considerably facilitate drug discovery.

When searching for peptide-based drug candidates, functional assay systems are important methodologies for revealing novel functional ligands of GPCRs. For example, quantification of second messengers has been used to quantify GPCR activities^12^. In addition, functional assay systems in which reporter genes are regulated by second messenger response elements have also been reported^12,13^. For example, in a previous study, we constructed a hGLP1R/NLuc-293 reporter cell line^13^, which secretes NanoLuc^14^ in response to the activation of the human glucagon-like peptide 1 receptor (hGLP1R)^15^. Such a system facilitates easy and sensitive detection of GPCRs activities, in turn facilitating the identification of agonists or antagonists of target GPCRs.

**Figure 1:**
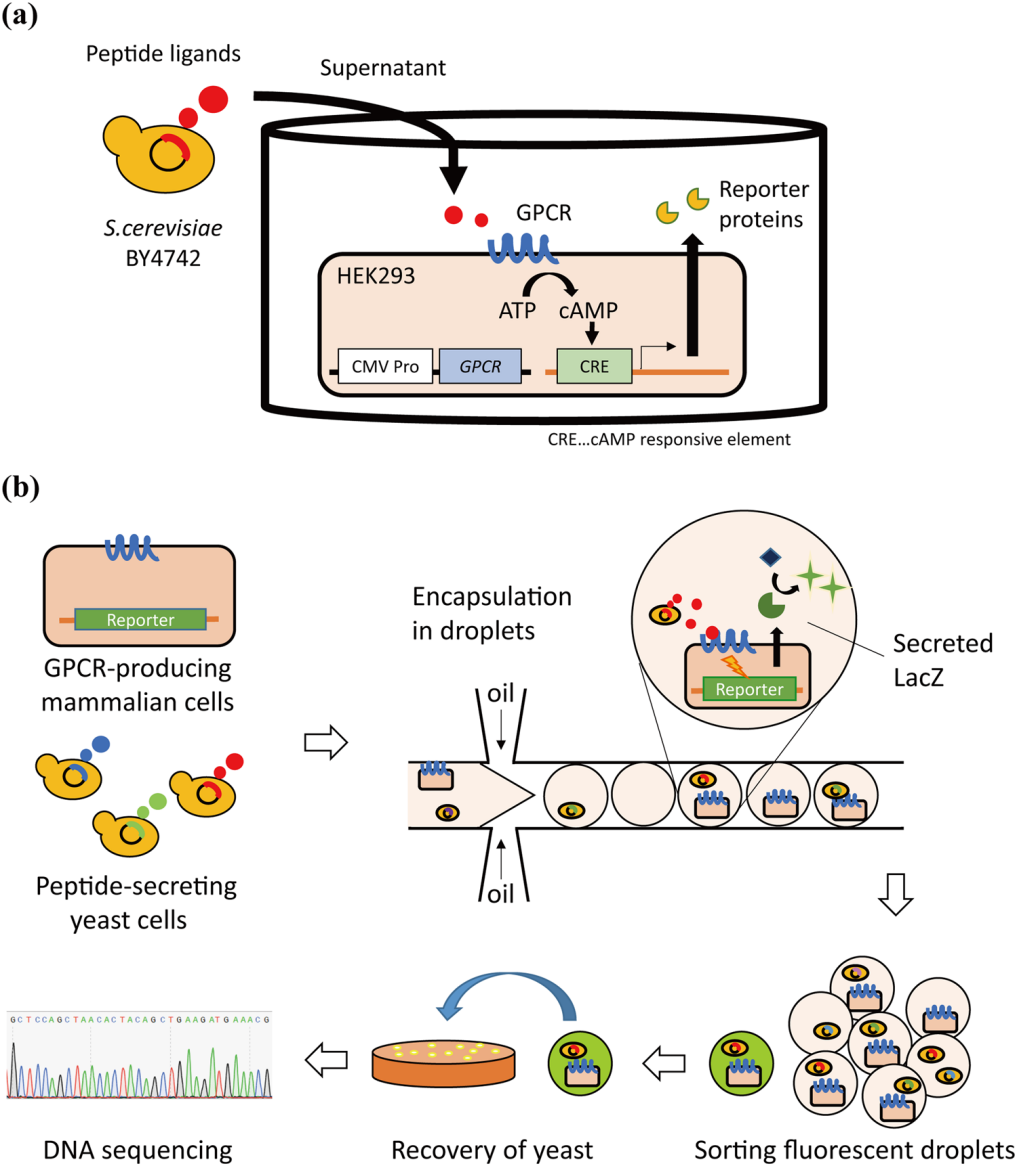
Schematic of the high-throughput functional cell-based assay using droplet microfluidics. (**a**) Functional cell-based assay using GPCR-producing reporter cells and ligand-secreting yeast cells. When peptide ligands secreted by yeast cells activate GPCRs produced by HEK293, a reporter gene is activated via cAMP response element (CRE). (**b**) High-throughput functional cell-based assay using droplet microfluidics. The reporter cells and the yeast cells secreting randomized peptide ligands are encapsulated in droplets and co-cultured. When a randomized peptide ligand secreted by a single yeast cell activates the reporter cell, resulting in the production of reporter proteins (LacZ), a droplet emits strong fluorescence. The fluorescent droplets are isolated, and the yeast cells producing functional peptide ligands are cultured on a plate medium. Finally, sequences of the functional peptide ligands are determined using DNA sequencing.

In the search for novel peptide ligands, designing a ligand library is an important process. At present, various studies have employed display technologies in the construction of peptide libraries because they can easily construct large peptide libraries at low costs^16–19^. However, in the display systems, steric hindrance could adversely affect the functions of the displayed peptides^20^. Secretion of peptides could be an alternative promising strategy for addressing the problem^13^ (Fig. 1a). Steric hindrance does not affect secreted peptides, which facilitates the evaluation of the inherent activities of the peptides. In a previous study, we developed a functional assay system using yeast cells secreting peptide ligands^13^. Such an approach permits the easy construction of a soluble peptide ligand library at relatively low cost.

Although the construction of peptide libraries has become simple, many studies have performed low throughput plate-based assays for evaluating the activities of peptide libraries^12,13^. Droplet microfluidic devices could be alternative promising tools for high-throughput assays^21^. Droplet microfluidic devices have micrometer-sized flow paths^22^, and can produce monodisperse droplets in the nanometer to micrometer diameter range at tens of thousands of droplets per second rates^22^. Droplet microfluidics is an effective approach for screening large libraries because it is compatible with fluorescence-activated sorting^23,24^. It has been reported that the cultivation of microorganisms and mammalian culture cells is possible using droplets^25,26^. In addition, various assays can be performed using droplets: screening of functional T-cell receptors^27^, screening of hybridoma cells which secrete functional antibodies^28^, and drug discovery and genomic applications^29,30^.

We have previously developed a functional assay system using combined mammalian cells, which produce NanoLuc in response to the activation of hGLP1R and yeast cells that secrete randomized peptides^13^; however, its throughput was low because we carried out functional assays using 96-well plates. In the present study, we attempt to construct a novel high-throughput cell-based assay for the identification of peptide ligands (Fig. 1b). To develop the system, we constructed a mammalian reporter cell line (hGLP1R/LacZ-293), which produces LacZ in response to the activation of hGLP1R as a model. Replacement of NanoLuc with LacZ enables high-throughput evaluation using fluorescence observation. In addition, we used a droplet microfluidics device to facilitate massively parallel functional assays. By combining an hGLP1R/LacZ-293 reporter cell line, a yeast library secreting randomized hGLP1R ligands, and a droplet microfluidic device, we identified functional ligands for hGLP1R. The methodology could be a useful platform for the discovery of novel peptide ligands of GPCRs.

## Results

### Construction of a LacZ reporter cell line for functional assay of hGLP1R

**Figure 2:**
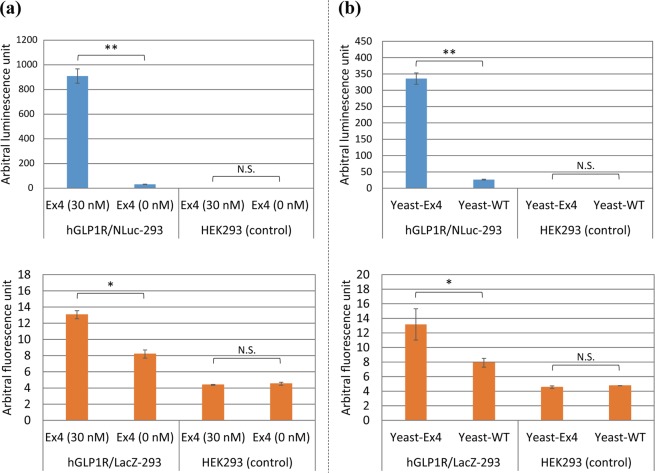
Functional cell-based assay for evaluating the reporter cell lines. (**a**) Functional cell-based assay using authentic exendin-4 (Ex4). hGLP1R/NLuc-293 and hGLP1R/LacZ-293 were cultured in DMEM media with or without 30 nM authentic Ex4. WT HEK293 was used as a control. Values are mean ± SD (n = 3). Two-tailed Student’s *t*-test was used to compare two groups (*p < 0.05, **p < 0.01). N.S., not significant. (**b**) Functional cell-based assay using culture supernatants of yeast cells secreting Ex4. hGLP1R/NLuc-293 and hGLP1R/LacZ-293 were cultured in DMEM media with culture supernatants of yeast cells producing Ex4 (Yeast-Ex4) or WT yeast cells (Yeast-WT). Values are mean ± SD (n = 3). Two-tailed Student’s *t*-test was used to compare two groups (*p < 0.05, **p < 0.01). N.S., not significant.

We constructed a human reporter cell line (hGLP1R/LacZ-293), which constitutively produces human GLP1R (hGLP1R), and inducibily secretes LacZ in response to the activation of hGLP1R. First, we compared hGLP1R/LacZ-293 and hGLP1R/NLuc-293 cell lines constructed in a previous study^13^. hGLP1R/NLuc-293 is a cell line that produced NanoLuc in response to the activation of hGLP1R. We cultured the cell lines in Dulbecco’s modified Eagle’s medium (DMEM) with or without 30 nM exendin-4 (Ex4), a representative agonist of GLP1R^31^. We observed significant reporter signals in both lines (Fig. 2a). The background signals of hGLP1R/LacZ-293 and hGLP1R/NLuc-293 (0 nM Ex4) were higher than that of HEK293 cells, probably due to leakage of *LacZ* or *NLuc* expression. Subsequently, we investigated whether the culture supernatants of Ex4-secreting yeast cells could activate the hGLP1R of hGLP1R/LacZ-293 and hGLP1R/NLuc-293 cell lines. We cultured the cell lines with or without the culture supernatants of Ex4-secretory yeast (yeast-Ex4) or wild-type (WT) yeast (yeast-WT) and we observed significant reporter signals in both lines (Fig. 2b). Although the signal-noise ratio of hGLP1R/LacZ-293 was lower than that of hGLP1R/NLuc-293, we concluded that hGLP1R/LacZ-293 could be used as a fluorescent reporter line for hGLP1R, because luciferase imaging has two drawbacks. First, we cannot detect luminescence if secreted luciferases run out of a limited amount of substrates in droplets. Second, the rate of photon production by luciferase is very low, hence luciferase imaging is not appropriate for high-throughput analysis.

### Functional assay of hGLP1R using floating hGLP1R/LacZ-293 cells

**Figure 3:**
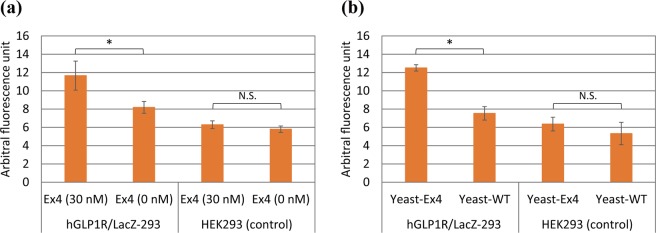
Functional cell-based assay using floating reporter cells. (**a**) Functional cell-based assay using authentic Exendin-4 (Ex4). The floating hGLP1R/LacZ-293 cells were suspended in a RPMI-1640 medium with or without 30 nM of authentic Ex4. Floating WT HEK293 was used as control. Values are mean ± SD (n = 3). Two-tailed Student’s *t*-test was used to compare two groups (*p < 0.05). N.S., not significant. (**b**) Functional cell-based assay by co-culture with yeast cells secreting Ex4. The floating mammalian hGLP1R/LacZ-293 or WT HEK293 cells were co-cultured with yeast cells producing Ex4 (Yeast-Ex4) or WT yeast cells (Yeast-WT). Values were mean ± SD (n = 3). Two-tailed Student’s *t*-test was used to compare two groups (*p < 0.05). N.S., not significant.

In Fig. 1, we used adherent reporter cells for functional assays. However, when reporter cells are encapsulated in droplets, they are in suspension. Therefore, we investigated hGLP1R of hGLP1R/LacZ-293 activity could be measured in suspension. We prepared floating hGLP1R/LacZ-293 cells in RPMI-1640 medium with or without 30 nM authentic Ex4. We observed a significant increase in fluorescence in the sample with authentic Ex4 (Fig. 3a). The result indicated that floating hGLP1R/LacZ-293 cells could respond to the activation of hGLP1R. Subsequently, we examined the potential of activating hGLP1R by co-culture with yeast cells secreting Ex4. We mixed yeast cells secreting Ex4 and hGLP1R/LacZ-293 cells in fresh RPMI-1640 containing LacZ substrate to obtain 4.5 × 10^5^ cells/50 μL and 5 × 10^3^ cells/50 μL, respectively. After incubation, we observed a significant increase in fluorescence when hGLP1R/LacZ-293 cells were co-cultured with yeast cells secreting Ex4 (Fig. 3b), suggesting the feasibility of functional cell-based assay by co-culture of hGLP1R/LacZ-293 and ligand-secreting yeast cells in droplets.

### Development of a functional assay system using droplet microfluidics

**Figure 4:**
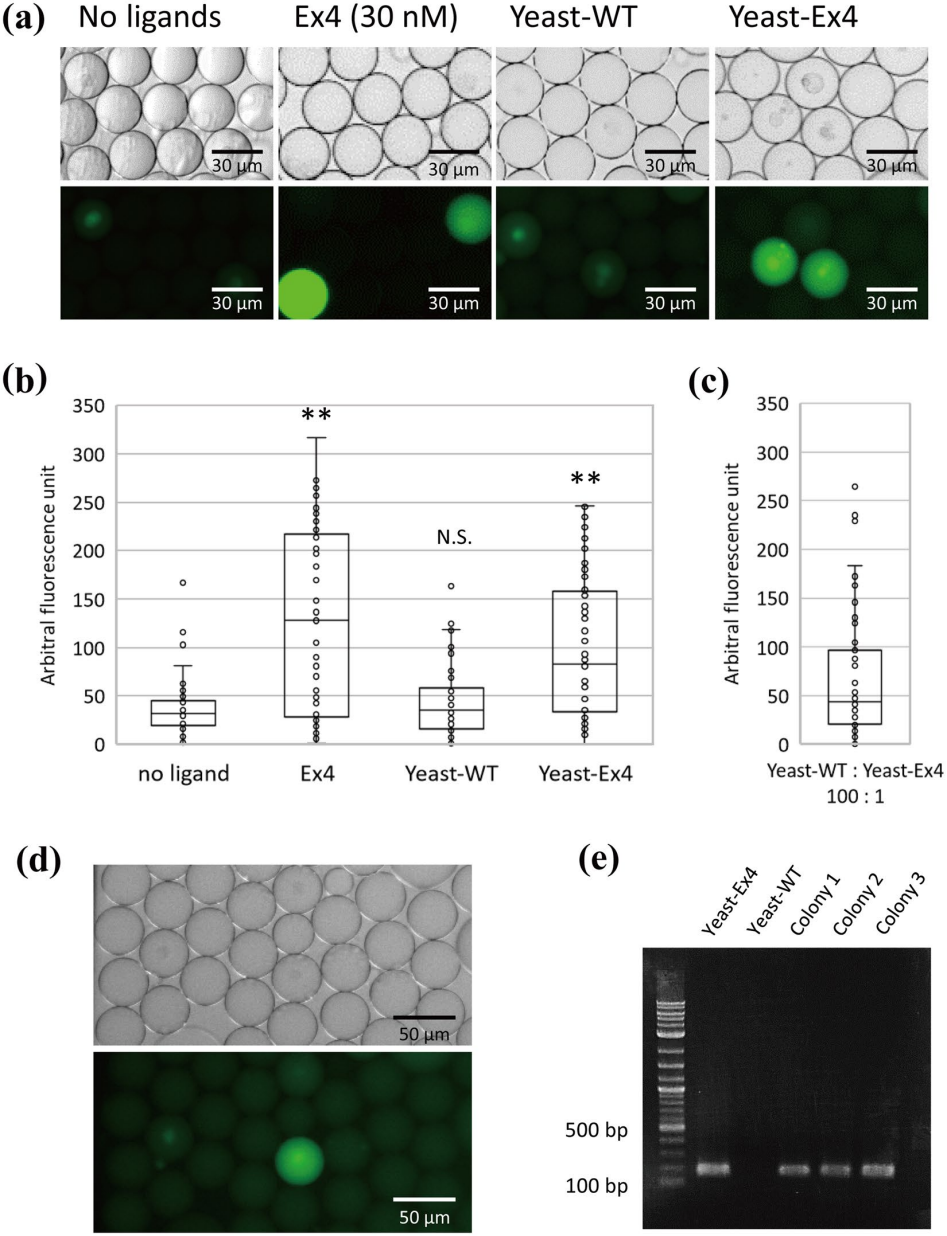
High-throughput functional cell-based assay using droplet microfluidics. (**a**) Representative fluorescence micrographs of droplets. The hGLP1R/LacZ-293 cells were encapsulated with no ligand, 30 nM authentic Exendin-4 (Ex4), WT yeast cells (Yeast-WT, 9.1 × 10^6^ cells/mL), or yeast cells producing Ex4 (Yeast-Ex4, 9.1 × 10^6^ cells/mL.). The images show the bright field images (upper image) and the fluorescence images (lower image). (**b**) Box plots of fluorescence intensity of each droplet in the experiment of (**a**). We analyzed at least 70 droplets containing a reporter cell for all samples. Statistical significance was determined by two-tailed Student’s *t*-test (**p < 0.01). N.S., not significant. (**c**) Box plots of fluorescence intensity of droplets. The hGLP1R/LacZ-293 cells were encapsulated with a mixture of yeast-WT cells and yeast-Ex4 cells at a ratio of 100:1. (**d**) A representative fluorescence image of droplets in the experiment of (**c**). The images show the bright field image (upper image) and the fluorescence image (lower image). (**e**) A colony-direct PCR was performed on yeast cells isolated in the experiment of (**c**,**d**). To determine whether colonies isolated from the strongly fluorescent droplet were yeast-WT or yeast-Ex4, a colony-direct PCR was performed on isolated cells. Yeast-Ex4 and yeast-WT were used as a positive control and a negative control, respectively.

To ascertain whether hGLP1R activity could be detected in droplets, mammalian hGLP1R/LacZ-293 cells were encapsulated in droplets using various media: DMEM medium (no ligand), DMEM containing authentic Ex4 (Ex4), DMEM containing WT yeast cells (Yeast-WT), and DMEM containing yeast cells secreting Ex4 (Yeast-Ex4). After incubating the droplets at 30 °C for 6 h, we observed fluorescence using a confocal microscope (Fig. 4a). When hGLP1R/LacZ-293 cells were encapsulated with authentic Ex4 or yeast cells secreting Ex4, significant increases in fluorescence intensity were observed (Fig. 4b). In the co-culture experiments, some droplets didn’t emit fluorescence. This is because droplets will emit fluorescence only when both yeast cells and mammalian reporter cells are encapsulated. Furthermore, some reporter cells did not well respond to the addition of Ex4, and box plots of droplets with “no ligand” and that with “Ex4” was partially overlapped (Fig. 4b). This is probably because cultured mammalian cells can show different traits even when they are derived from a monoclonal cell population.

Subsequently, we evaluated whether yeast cells that activate the reporter cell line could be isolated using fluorescence microscopy analysis. WT yeast cells and yeast cells secreting Ex4 were mixed at a ratio of 100 to 1 and suspended in DMEM to a 9.1 × 10^6^ yeast cells/mL concentration. The mixture of yeast cells was encapsulated in droplets with the reporter cell line adjusted to a 5.0 × 10^6^ cells/mL concentration. The fluorescence intensities of droplets were quantified using a confocal microscope (Fig. 4c,d). We isolated single droplet with strong fluorescence, and cultured yeast cells among the droplet. Three colonies were formed on the plate media. Using colony-direct PCR, we observed that isolated yeast cells were Ex4-secreting cells (Fig. 4e and Supplementary Information 2), which indicated that we could isolate yeast cells secreting functional peptide ligands using the system.

### Discovery of novel functional ligand sequences using droplet microfluidics

**Figure 5:**
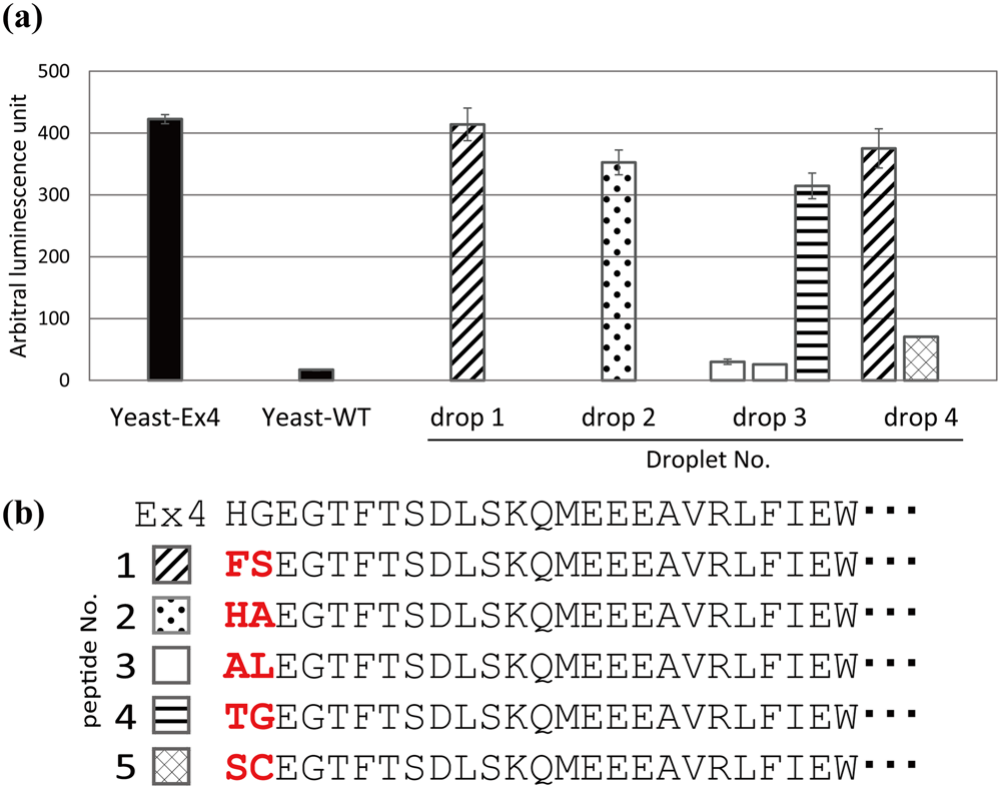
Functional evaluation of yeast cells producing randomized Exendin-4 (Ex4) variants isolated by high-throughput functional cell-based assay using droplet microfluidics. (**a**) Evaluation of the activity of the randomized Ex4 variants secreted by isolated yeast cells. The adherent hGLP1R/NLuc-293 cells were incubated with culture supernatants of the isolated yeast cells, and luminescence intensity was quantified. Yeast-Ex4 and Yeast-WT were used as a positive control and a negative control, respectively. Values were given as mean ± SD (n = 3). (**b**) Sequences of the isolated Ex4 variants. Amino acids shown in red indicate the N-terminal two amino acids.

The N-terminal two amino acids of Ex4 are involved in the activation of GLP1R^32,33^, and mutations in the N-terminal two amino acids have resulted in decreases in agonist activity^32,33^. Therefore, we explored whether we could identify novel Ex4 variant sequences randomized at the N-terminal two amino acids using a functional droplet assay system. We constructed a yeast library secreting randomized Ex4 variants using gap repair cloning^34^. In this study, we completely randomized two amino acids of N-terminus. It was expected that most mutant peptides were non-active, and that the original Ex4 appeared at the probability of 0.20% in the mutant library. We then encapsulated the yeast cells and the mammalian hGLP1R/LacZ-293 cells in droplets, and observed that some droplets exhibited strong fluorescence. We isolated six droplets from about one hundred thousand droplets and cultured yeast cells in separate YPD plates. Among a total of six plates, we obtained two plates with one colony (drop 1 and 2), one plate with three colonies (drop 3), one plate with two colonies (drop 4), and two plates with no colonies (drops 5 and 6). We cultured the yeast cells, and their culture supernatants were added to adherent hGLP1R/NLuc-293 cells to re-evaluate the activities of the peptides secreted by the yeast cells. At least one yeast cell in each droplet secreted a functional peptide ligand (Fig. 5a). The sequences of the ligands were determined by Sanger sequencing, and all peptides had an N-terminal sequence that was different from that of WT Ex4 (Fig. 5b).

**Figure 6:**
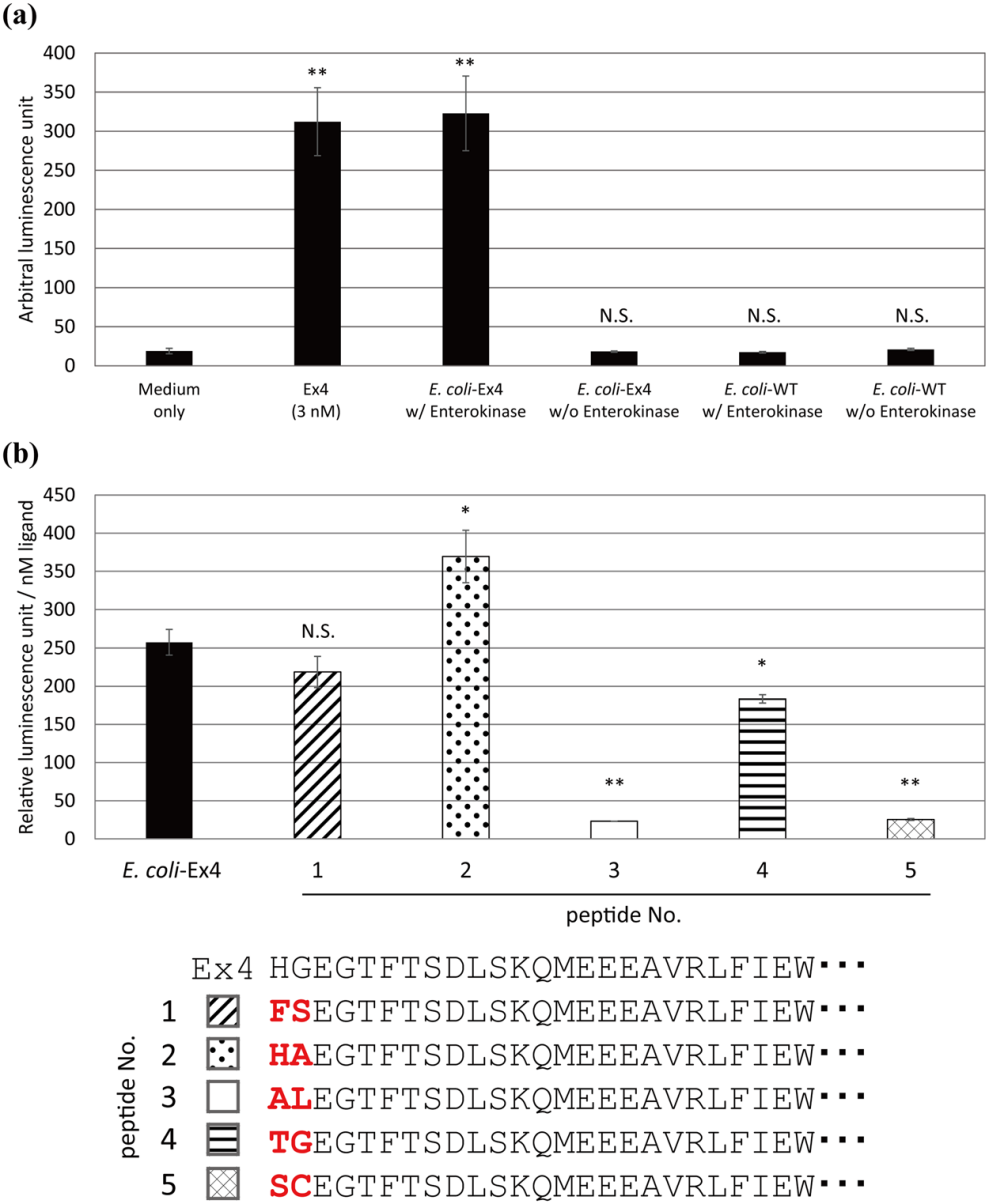
Functional evaluation of Exendin-4 (Ex4) variants produced by *E*. *coli.* (**a**) The functional assay using WT Ex4 produced by *E*. *coli*. We cultured *E*. *coli* producing WT Ex4 fused with a FLAG sequence at the N-terminal (*E*. *coli*-Ex4) and WT *E*. *coli* (*E*. *coli*-WT). The cell lysates were purified using anti-FLAG resin, reacted with or without enterokinase, and assayed with the adherent hGLP1R/NLuc-293 cells. DMEM media with or without 3 nM authentic Ex4 were used as a positive control and a negative control. Values are mean ± SD (n = 3). Two-tailed Student’s *t*-test was used to compare two groups (**p < 0.01). N.S., not significant. (**b**) Activities of each of the Ex4 variants produced by *E*. *coli*. Relative luminescence units were corrected based on concentrations of each peptide. Values are mean ± SD (n = 3). Two-tailed Student’s *t*-test was used to compare two groups (*p < 0.05, **p < 0.01). N.S., not significant.

To investigate whether the activity of the peptides was not influenced by the post-translational modifications by yeast or differences in secretion efficiencies, WT Ex4 and Ex4 variants fused with an N-terminal FLAG sequence were produced using *Escherichia coli*. The peptides were purified using anti-FLAG resin, and reacted with enterokinase, which cleaves the peptides after the FLAG sequence to expose free N-terminal of Ex4 peptides. We added the purified WT Ex4 produced by *E*. *coli* with or without enterokinase reactions to the hGLP1R/NLuc-293 cells, and observed a significant increase in luminescence only when the peptides were cleaved by enterokinase (Fig. 6a). The result indicated that WT Ex4 produced by *E*. *coli* is functional and a free N-terminal was vital for the activation of GLP1R. The amounts of the purified WT Ex4 and Ex4 variants produced by *E*. *coli* were then quantified using liquid chromatography coupled to mass spectrometry, and their activities were evaluated using the hGLP1R/NLuc-293 cells. The Ex4 variants produced by *E*. *coli* had an activation capacity higher or comparable to that of WT Ex4 produced by *E*. *coli* (Fig. 6b). Though we did not obtain a variant with an at least 2-fold increased activity compared to Ex4, this is probably because Ex4 is a very strong agonist which was isolated from the venome of the Gila monster^31^, and we think it is worthwhile that we succeeded in improving the activity of such a strong agonist.

## Discussion

In our previous study^13^, we developed a functional cell-based assay using a reporter mammalian cell line (hGLP1R/NLuc-293) and a yeast library secreting peptide ligand. However, the system used a luminescence assay system in a 96-well format, leading to low throughput. In the present study, we modified the system and replaced the reporter protein from NanoLuc with LacZ, which facilitates the evaluation of ligand activity using fluorescence. Although the replacement resulted in a decrease in the signal-noise ratio, we could identify functional droplets using fluorescence microscopy (Fig. 4a and b). The replacement facilitated compatibility with fluorescence-activated sorting, which could lead to improvements in throughput identification of functional ligands. In our co-culture assay system, compounds produced by yeast cells could affect the viability of mammalian cells. In our previous study, addition of culture supernatants of yeast cells containing GLP1R agonists successfully induced expression of NLuc of mammalian reporter cells^13^. In addition, this study showed that co-cultivation of yeast cells and mammalian cells could significantly increase reporter signals (Figs 3b and 4). Hence we assumed that potential toxicity of yeast metabolites didn’t disturb our purpose to screen yeast cells secreting functional ligands. Ever if the mammalian reporter cells were dead, fluorescent molecules produced by LacZ continue existing in droplets, hence we could observe fluorescence signals and isolate yeast cells. In this study, we didn’t check how fast the hGLP1R/LacZ-293 cells respond to the activation of hGLP1R. Optimization of incubation times could lead to more quantitative evaluation of agonists’ activity which enables to distinguish strong agonist and weak agonist.

A droplet microfluidic device can produce highly monodisperse droplets in the nanometer to micrometer diameter range, at rates of tens of thousands of droplets per second^22^. In the present study, we optimized cell concentrations for better encapsulation of the reporter cells and yeast cells. Based on the results, the concentration of reporter cells was set to 5.0 × 10^6^ cells/mL (Table S1) and that of yeast cells to 9.1 × 10^6^ cells/mL (Table S2). At such concentrations, the probability that at least one mammalian cell was included in a droplet was 6.55%, and the probability that at least one yeast cell was included in a droplet was 11.29%, hence the probability that both were included in a droplet was 0.74%. In our previous study, we separately cultured each ligand-secreting yeast mutant, and manually transferred culture supernatants to a 96-well plate^13^, hence it was very laborious to evaluate even one hundred of yeast mutants. The droplet-based approach developed in this study enabled us to evaluate thousands of ligand-secreting yeast mutants, because the microfluidics device could generate droplets containing both mammalian reporter cells and ligand-secreting yeast cells at a speed of about several tens of droplets/sec. Although we manually observed droplets with a fluorescence microscope, we could evaluate ten times or more ligand-secreting yeast mutants compared to the previous study.

To provide proof-of-principle for our system, we attempt to identify novel ligand sequences using a randomized yeast library. In our experiments, we isolated six droplets and recovered yeast cells from four droplets (Fig. 5a). Some single yeast cells were assumed to have been lost during the isolation process using a micromanipulator. We also found that some droplets included several yeast cells (Fig. 5a), which could be because multiple cells can be encapsulated in one droplet (Table S2). To exclude such non-specific yeast cells, screening yeast cells over several cycles can be beneficial^35^.

Based on the results of the experiments using *E*. *coli* as a host for producing peptides, we demonstrated that the activities of the identified Ex4 variants were not influenced by post-translational modifications or differences in secretion efficiencies (Fig. 6b). A previous study revealed that the hydrogen bonding of Glu387 of GLP1R and histidine at the N-terminus of the ligand is vital for the activity of GLP1R^32^. In the present study, functional peptides contained Ser (Fig. 6b, peptide 1) or Thr (Fig. 6b, peptide 4) at the N-terminus, suggesting that the amino acids were selected due to hydrogen bonding with hGLP1R.

It is considered that further improvement of the throughput will be necessary to comprehensive characterization of the peptide’s mutation space. To improve the throughput, there is a method for increasing the generation speed of droplets^36^ and methods of increasing the proportions of droplets containing cells^29,37^. In addition, fluorescence-activated cell sorting on a microfluidics will improve the throughput to retrieve fluorescence-positive droplets.

In conclusion, we demonstrated that functional assay of hGLP1R could be performed by temporarily co-culturing the reporter mammalian cells and the yeast cells in a bulk assay. In addition, we encapsulated the mammalian cells and yeast cells in water-in-oil droplets using a droplet microfluidic device for high-throughput identification of peptide ligands against hGLP1R. We identified novel functional ligands, one of which exhibited higher activity than that of Ex4 (Fig. 6b, peptide 2). The result suggested that the droplet assay system constructed in the present study could be effective in the identification of novel peptide ligands. In future studies, we will optimize our system to improve encapsulation efficiency and throughput by combining fluorescence-activated sorting and next-generation sequencers.

## Methods

### Construction of reporter cells

To construct a reporter cell line that constitutively produces human GLP1R (hGLP1R) and inducibily secretes NanoLuc in response to the activation of hGLP1R, pIRES-hGLP1R, and pCRE-NLuc^13^, were co-transfected in HEK293 cells (European Collection of Cell Culture, Salisbury, UK) using Xfect™ (Clontech Laboratries, Inc., Mountain View, CA, USA). The transfected cells were cultured in Dulbecco’s Modified Eagle Medium (DMEM) (Nacalai Tesque, Kyoto, Japan) containing 10% FBS (GE Healthcare, Little Chalfont, UK), 1% penicillin-streptomycin (Sigma-Aldrich, St. Louis, MO, USA), 400 µg/mL G418 (Nacalai Tesque), and 0.1 µg/mL puromycin (Wako Pure Chemical Industries, Osaka, Japan). The reporter cells were selected to establish a stable cell line harboring *GLP1R* and secretory *NanoLuc* genes (hGLP1R/NLuc-293)^13^. To construct a reporter cell line that secretes LacZ in response to the activation of hGLP1R, pIRES-hGLP1R^13^, and pCRE-LacZ were co-transfected in HEK293 cells, and a reporter cell line, hGLP1R/LacZ-293, was selected using a medium containing G418 and puromycin. A full sequence of pIRES-hGLP1R and partial sequences of pCRE-NLuc and pCRE-LacZ are provided in Supplementary Information 3.

### Construction of yeast cells secreting peptide ligands

*Saccharomyces cerevisiae* BY4742 [*MATα*, *his3*Δ*1*, *leu2*Δ*0*, *lys2*Δ*0*, *ura3*Δ*0*] (GE Healthcare) was used for the secretion of a hGLP1R agonist, Exendin-4 (Ex4, produced by the Gila monster^31^) and randomized Ex4 variants. To construct an Ex4-secretory yeast, pULS-Ex4^13^ was transformed into the BY4742 strain using the Frozen-EZ Yeast Transformation-II Kit (Zymo Research, Orange, CA, USA). Yeast cells secreting randomized Ex4 variants were constructed using gap repair cloning^34^ as follows. pULS plasmid^13^ was digested with *Eco*RI and *Kpn*I to obtain a linear vector fragment. Insert DNA fragments encoding Ex4 genes whose two N-terminal amino acids were randomized were PCR-amplified from pULS-Ex4 using the following primers: 5′-GCCAGCATTGCTGCTAAAGAAGAAGGGGTATCTTTGGATAAAAGANNKNNKGAAGGAACATTTACCAGTGACTTGTC-3′ and 5′-AGTCTCTTTCTCGGTCTAGCTAGTTTTACT-3′. The BY4742 strains were transformed using the linear vector fragments (500 ng) and the randomized insert fragments (250 ng) for gap repair cloning. The transformants were selected on synthetic dextrose solid (SDC) medium without uracil [0.67% (*w/v*), yeast nitrogen base without amino acids, 2% (*w/v*) glucose, 1% (*w/v*) casamino acids, 0.002% (*w/v*) adenine, 0.002% (*w/v*) l-tryptophan, and 2% (*w/v*) agar]. Full sequences of the plasmids used in the present study are provided in Supplementary Information 3.

### Construction of *E*. *coli* strains producing peptide ligands

pKPY514^38^, with an isopropyl-β-d-thiogalactoside (IPTG) inducible T5 promoter, was a gift from David Tirrell (Addgene plasmid #62598; http://n2t.net/addgene:62598; RRID:Addgene_62598). pKPY514 was digested using *Eco*RI and *Hin*dIII to obtain a linear vector fragment. Plasmids encoding Ex4 variants fused with a FLAG sequence at the N-terminus were obtained using In-Fusion^®^ HD Cloning Kit (Clontech Laboratries, Inc.). The resultant plasmids were named pKPY-Ex4, pKPY-FS-Ex4, pKPY-HA-Ex4, pKPY-AL-Ex4, pKPY-TG-Ex4, and pKPY-SC-Ex4. *E*. *coli* BL21 Star (DE3) [*F- ompT hsdS*_B_ (r_B_-m_B_-) *gal dcm rne131* (DE3)] (Thermo Fisher Scientific, Waltham, MA, USA) was used for the production of WT Ex4 and Ex4 variants. The transformants were selected on Luria-Bertani (LB) medium [1% (*w/v*) tryptone, 0.5% (*w/v*) yeast extract, 1% (*w/v*) sodium chloride, and 2% (*w/v*) agarose] containing 50 µg/mL kanamycin. Full sequences of pKPY514 and pKPY-Ex4 and partial sequences of the other plasmids are provided in Supplementary Information 3.

### Preparation of media containing hGLP1R ligands secreted by yeast cells

To prepare media containing hGLP1R ligands secreted by yeast cells, the yeast cells were cultured in a 96-well plate (Thermo Fisher Scientific) containing 300 μL of SDC medium at 30 °C for 48 h. Thereafter, yeast cells were spun down and the medium was exchanged with 250 μL DMEM or RPMI-1640 (Nacalai Tesque) and incubated at 30 °C for 12 h. DMEM medium was used when the reporter cells were in an adherent state and RPMI-1640 medium was used when the reporter cells were in suspension.

### Activation of hGLP1R in various experimental procedures

To perform a functional assay using the adherent hGLP1R/NLuc-293 cell line, hGLP1R/NLuc-293 cells were seeded at 5 × 10^3^ cells/100 µL in a 96-well plate (Thermo Fisher Scientific) and cultured at 37 °C for 24 h. Thereafter, the medium was exchanged with 50 µL DMEM containing a hGLP1R ligand, and further cultured at 37 °C for 12 h. Afterwards, 50 µL luciferase substrate (Nano-Glo™ Luciferase Assay System, Promega, Fitchburg, WI, USA) was added, and luminescence intensity was measured using a Fluoroskan Ascent™ Microplate Fluorometer (Thermo Fisher Scientific).

To perform a functional assay using the adherent hGLP1R/LacZ-293 cell line, hGLP1R/LacZ-293 cells were seeded at 5 × 10^3^ cells/100 µL in a 96-well plate (Thermo Fisher Scientific) and cultured at 37 °C for 24 h. The medium was then exchanged with 50 µL DMEM containing a hGLP1R ligand and 50 µM 5-chloromethylfluorescein di-β-D-galactopyranoside (CMFDG, Thermo Fisher Scientific). After 12 h incubation at 37 °C, fluorescence intensity was measured using Fluoroskan Ascent™ Microplate Fluorometer.

An assay using floating hGLP1R/LacZ-293 was carried out using the following procedure. The hGLP1R/LacZ-293 cells were suspended in RPMI-1640 medium containing a hGLP1R ligand and CMFDG (50 µM) at a concentration of 5 × 10^3^ cells/50 µL and then adequately stirred by pipetting. Immediately after the modification, the solution was placed in a 96-well plate and incubated at 37 °C for 12 h, and fluorescence intensity was measured.

Co-culture of floating hGLP1R/LacZ-293 and ligand-secreting yeast cells was carried out using the following procedure. The yeast cells were suspended in 5 mL SDC medium and cultured at 37 °C for 6 h. Thereafter, the yeast cells and hGLP1R/LacZ-293 cells were suspended in RPMI-1640 to obtain 4.5 × 10^5^ cells/50 µL and 5 × 10^3^ cells/50 µL, respectively. CMFDG was then added to the medium to a volume of 50 µM and adequately stirred by pipetting. Immediately after the modification, the solution was incubated in a 96-well plate at 37 °C for 12 h, and fluorescence intensity was measured.

### Functional cell-based assay in droplet

A single-inlet microfluidic device was designed according to previous reports^39,40^ (Fig. S1). In brief, polydimethylsiloxane (PDMS, Sylgard 184, Dow Corning Corp, Midland, MI, USA) and its cross-linker were mixed at a ratio of 10:1 (w/w), degassed, poured onto a master mold, and cured. The cured PDMS was peeled from the mold and punched with 0.75 mm biopsy punch (World Precision Instruments, Sarasota, FL, USA). The PDMS slab was bonded to a glass slide by plasma treatment (Plasma Cleaner PDG-32G; Harrick Scientific, Pleasantville, NY, USA), and baked for at least 30 min at 70 °C. The microfluidics device, an air compressor, Mitos P-Pump Basic (Dolomite, Charleston, MA, USA) were connected, and the pressure was set to be about 150 mbar for aqueous phase and about 300 mbar for oil phase. To generate droplets, cell suspension and a carrier phase (FC40 containing 5% (v/v) of the surfactant Pico-Surf1, Dolomite), were pumped into the cross-junction.

To investigate the optimal concentration of hGLP1R/LacZ-293 cells for droplet encapsulation, hGLP1R/LacZ-293 cells were stained using 20 µg/mL 4′6-diamidino-2-phenylindole dihydrochloride (DAPI, Nacalai Tesque). The stained cells were then suspended in PBS to obtain 1.0 × 10^6^, 2.5 × 10^6^, 5.0 × 10^6^, 7.5 × 10^6^, and 10 × 10^6^ cells/mL. The suspensions were enclosed in droplets using the single inlet microfluidic device^39,40^. The number of cells encapsulated in each droplet was determined by image analysis using a confocal microscope (Fig. S2 and Table S1). To investigate the optimal concentration of yeast cells for droplet encapsulation, *S*. *cerevisiae* BY4742 cells were stained using Fungi-Fluor (Polysciences, Inc., Warrington, PA, USA), and suspended in PBS 1.9 × 10^6^, 5.5 × 10^6^, 9.1 × 10^6^, 12.7 × 10^6^, and 16.3 × 10^6^ cells/mL concentrations. The number of cells encapsulated in each droplet was determined by image analysis using a confocal microscope (Fig. S3 and Table S2). In addition, dividing or aggregated yeast cells populations were determined by observing same yeast suspension (Fig. S4 and Table S3).

As a proof-of-principle experiment, hGLP1R/LacZ-293 was suspended at 5.0 × 10^6^ cells/mL in the following media: DMEM, DMEM containing authentic Ex4, DMEM containing WT *S*. *cerevisiae* (Yeast-WT), or DMEM containing Ex4-secreting *S*. *cerevisiae* (Yeast-Ex4). The concentration of yeast cells was set to be 9.1 × 10^6^ cells/mL. CMFDG was added in the media to realize a concentration of 50 µM. After encapsulation, the droplets were incubated at 30 °C for 6 h. Fluorescence intensity of each droplet was calculated by image analysis under a confocal laser scanning fluorescence microscope.

As a competitive assay, yeast-WT and yeast-Ex4 were mixed at a ratio of 100 to 1 in DMEM at 9.1 × 10^6^ cells/mL with 50 µM CMFDG. hGLP1R/LacZ-293 cells were suspended in the solution at a 5.0 × 10^6^ cells/mL concentration. The solution was enclosed in droplets, and incubated at 37 °C for 6 h. Fluorescence intensity was calculated by image analysis. In addition, single droplet emitting strong fluorescence was isolated using a micromanipulator (Drummond Scientific Co., Broomall, PA, USA), and incubated in 200 µL of YPD medium at 30 °C for 4 h. The yeast cells were then cultured on YPD plate medium. Colony-direct PCR was performed using the following PCR primers: 5′-GCCAGCATTGCTGCTAAAGAAGAAG-3′ and 5′-CCAAGTCGACTTACGATGGTG-3′.

### High-throughput evaluation of Ex4 variants using droplet microfluidics

A yeast library producing Ex4 variants constructed by gap repair cloning was cultured in SDC medium at 30 °C for 6 h. hGLP1R/LacZ-293 cells and the yeast cells were then suspended in RPMI-1640 medium with 50 µM CMFDG to obtain 5.0 × 10^6^ cells/mL and 9.1 × 10^6^ cells/mL concentrations, respectively. The medium was enclosed in droplets, and incubated at 37 °C for 6 h. Then, 10 μL of the droplets were put on a slide glass. We manually observed about 20 thousand droplets with a fluorescence microscope (IX71, Olympus, Tokyo, Japan), and a droplet with strong fluorescence was isolated by a micromanipulator (Drummond Scientific Co.). This process was repeated six times to isolate six fluorescence droplets. Yeast cells in the fluorescence droplets were isolated as described above. The sequences of isolated Ex4 variants were determined by Sanger sequencing.

To verify the activities of the identified Ex4 variants, we prepared variant peptides using *E*. *coli* as follows. *E*. *coli* strains harboring plasmids encoding Ex4 variants were cultured in 5 mL LB medium containing 50 µg/mL kanamycin. Thereafter, 500 μL of the culture solution was added to 5 mL fresh LB medium and the solution incubated at 37 °C for 1 h. IPTG was then added to the solution to obtain a final volume of 1 mM, and *E*. *coli* cells were further cultured for 4 h. The *E*. *coli* cells were suspended in 150 μL PBS and disrupted using Bioruptor UCD-250 (Cosmo Bio Co., Ltd., Tokyo, Japan). The ligand peptides were purified using ANTI-FLAG M2 Affinity Gel (Sigma-Aldrich), digested with enterokinase, and diluted 100-fold with DMEM medium. The resultant solutions were used as media containing hGLP1R ligands produced by *E*. *coli*. The purified ligands were mixed with adherent hGLP1R/NLuc-293 to confirm their activities. Concentrations of the ligands were determined using selected reaction-monitoring against FLAG peptides using liquid chromatography (Nexera UHPLC/HPLC System; Shimadzu, Kyoto, Japan)-triple-quadrupole mass spectrometry (LCMS-8060; Shimadzu). The calibration curve used for quantification was obtained with standard FLAG peptides (Wako Pure Chemical Industries).

## Supplementary information

Supplementary Information 1

Supplementary Information 2

Supplementary Information 3
