# Supplementary material for: High-throughput identification of peptide agonists against GPCRs by co-culture of mammalian reporter cells and peptide-secreting yeast cells using droplet microfluidics
Source: Sci Rep. 2019 Jul 29;9:10920. doi: 10.1038/s41598-019-47388-x (PMC6662714; doi:10.1038/s41598-019-47388-x)
Supplement: Supplementary file 1 — Supplementary Information 1 [file 41598_2019_47388_MOESM1_ESM.docx]

**TITLE**

High-throughput identification of peptide agonists against GPCRs by co-culture of mammalian reporter cells and peptide-secreting yeast cells using droplet microfluidics

**AUTHORS AND AFFILIATONS**

Kenshi Yaginuma^1^, Wataru Aoki^1,2,3^, Natsuko Miura^4^, Yuta Ohtani^1^, Shunsuke Aburaya^1,5^, Masato Kogawa^6,7^, Yohei Nishikawa^6^, Masahito Hosokawa^3,8^, Haruko Takeyama^6,7,8^, Mitsuyoshi Ueda^1,2,*^

^1^Division of Applied Life Sciences, Graduate School of Agriculture, Kyoto University, Sakyo-ku, Kyoto 606-8502, Japan

^2^JST, CREST, 7 Goban-cho, Chiyoda-ku, Tokyo 102-0076, Japan

^3^JST, PRESTO, 7 Goban-cho, Chiyoda-ku, Tokyo 102-0076, Japan

^4^Graduate School of Life and Environmental Sciences, Osaka Prefecture University, 1-1 Gakuen-cho, Naka-ku, Sakai, Osaka 599-8531, Japan

^5^Japan Society for the Promotion of Science, 5-3-1 Kojimachi, Chiyoda-ku, Tokyo 102-0083, Japan

^6^Department of Life Science & Medical Bioscience, School of Advanced Science and Engineering, Waseda University, Shinjuku-ku, Tokyo 169-8555, Japan

^7^Computational Bio Big-Data Open Innovation Laboratory, AIST-Waseda University, 3-4-1 Okubo, Shinjuku-ku, Tokyo, 169–0072, Japan

^8^Institute for Advanced Research of Biosystem Dynamics, Waseda Research Institute for Science and Engineering, Waseda University, Shinjuku-ku, Tokyo 169-8555, Japan

*Correspondence should be addressed to: Mitsuyoshi Ueda

Tel.: +81-75-753-6495; Fax: +81-75-753-6112; E-mail: miueda@kais.kyoto-u.ac.jp


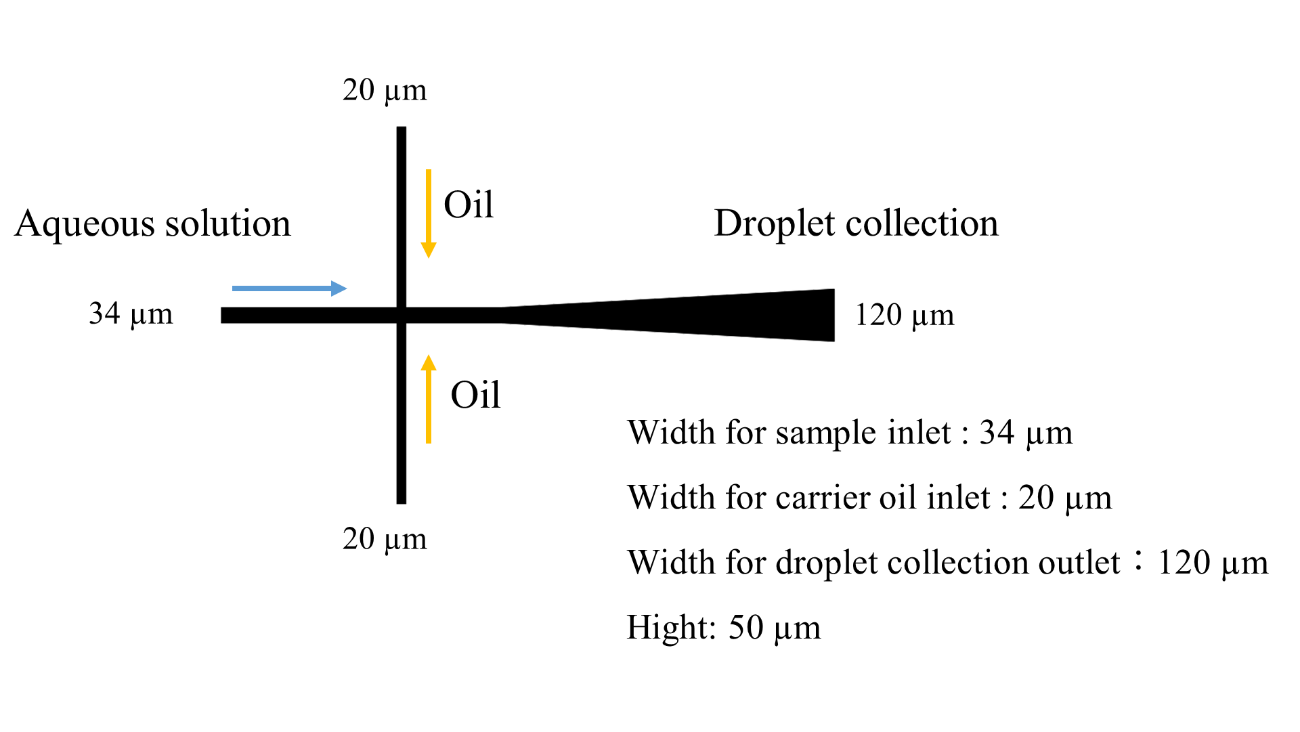


**Figure S1.** Design of a single-inlet microfluidic droplet generator. The cross-junction was designed to be 34 μm width for aqueous solution and 20 μm width for oil solution.


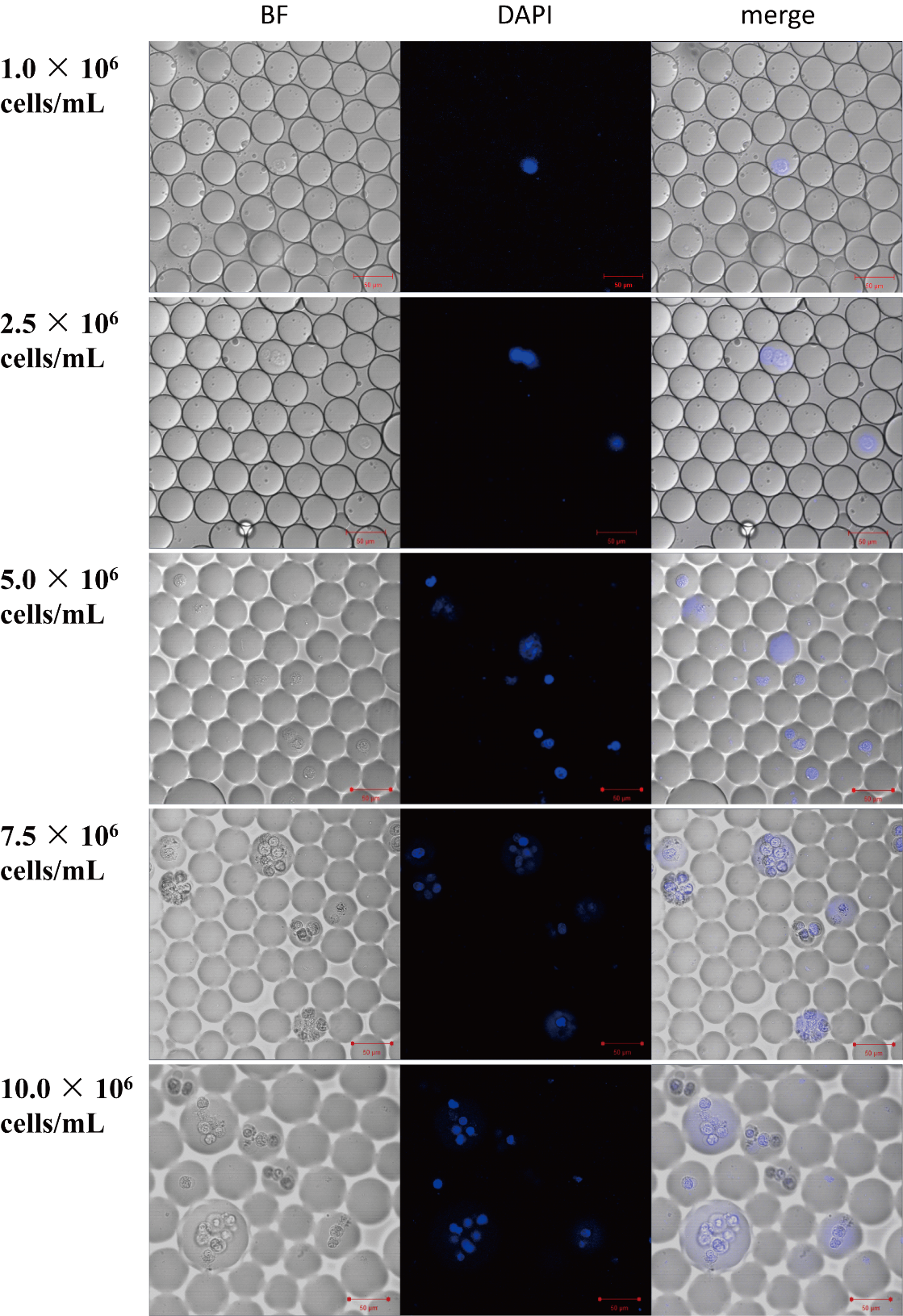
**Figure S2.** Images of droplets encapsulating hGLP1R/LacZ-293 cells. To investigate the relationship between concentrations of the cells in media and the number of cells encapsulated in each droplet, we generated droplets using various mammalian hGLP1R/LacZ-293 cell suspension concentrations.　The DAPI-stained cells were suspended in PBS 1.0 × 10^6^ cells/mL, 2.5 × 10^6^ cells/mL, 5.0 × 10^6^ cells/mL, 7.5 × 10^6^ cells/mL, or 10.0 × 10^6^ cells/mL concentrations, and these cell suspensions were encapsulated in droplets with a diameter of approximately 30 µm using a single inlet microchannel. Observation was carried out by using a confocal laser scanning fluorescence microscope (LSM700, Carl Zeiss, Oberkochen, Germany). The images show the bright field images (BF, left column), the DAPI fluorescence images (DAPI, center column) and the merged images (merge, right column).

**Table S1.** Relationships between concentrations of GLP1R/LacZ-293 cells and the number of cells encapsulated in each droplet. The ratio of cell-containing droplets increased with increasing cell concentration when concentrations were 5.0 × 10^6^ cells/mL or less. At concentrations of 7.5 × 10^6^ cells/mL or more, it was not possible to accurately determine the number of cells encapsulated in each droplet since cell aggregation occurred and rupture or fusion of droplets were observed.

| Concentration |  | Total | Containing  0 cell | Containing  1 cell | Containing  2 cells | Containing  3 cells | Containing  4 cells | Containing  5 cells |
| --- | --- | --- | --- | --- | --- | --- | --- | --- |
| 1.0 × 10^6^  cells/mL | Number of droplets | 1011 | 996 | 10 | 3 | 2 | 0 | 0 |
|  | Ratio (%) |  | 98.51 | 0.99 | 0.30 | 0.20 | 0.00 | 0.00 |
|  | Theoretical ratio^※^ (%) |  | 98.02 | 1.96 | 0.02 | 0.00 | 0.00 | 0.00 |
| 2.5 × 10^6^  cells/mL | Number of droplets | 1385 | 1336 | 38 | 7 | 3 | 1 | 0 |
|  | Ratio (%) |  | 96.46 | 2.74 | 0.51 | 0.22 | 0.07 | 0.00 |
|  | Theoretical ratio^※^ (%) |  | 95.12 | 4.76 | 0.12 | 0.00 | 0.00 | 0.00 |
| 5.0 × 10^6^  cells/mL | Number of droplets | 1267 | 1184 | 44 | 17 | 9 | 12 | 1 |
|  | Ratio (%) |  | 93.45 | 3.47 | 1.34 | 0.71 | 0.95 | 0.08 |
|  | Theoretical ratio^※^ (%) |  | 90.48 | 9.05 | 0.45 | 0.02 | 0.00 | 0.00 |

^※^The theoretical ratio was calculated assuming Poisson distribution.


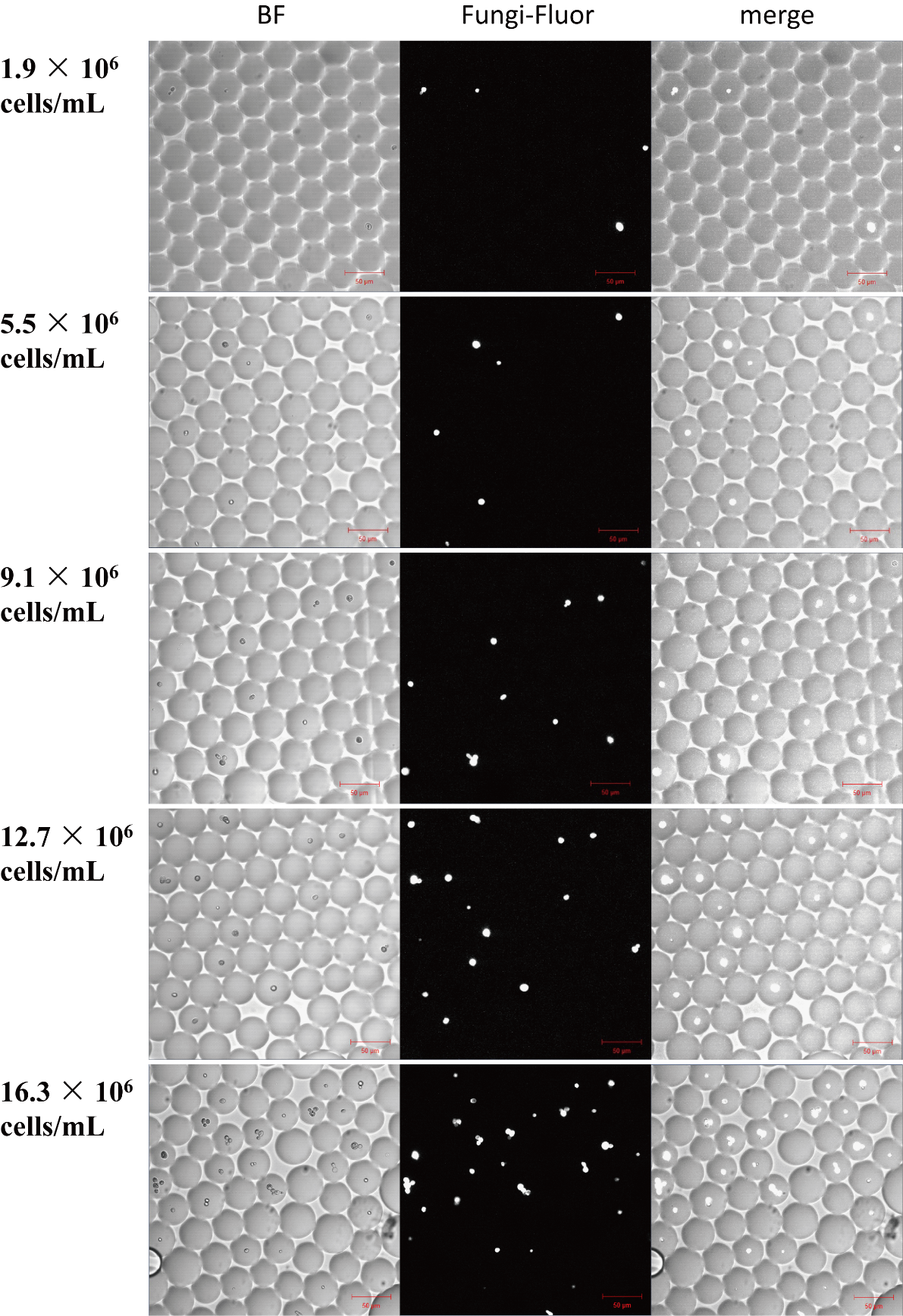
**Figure S3.** Images of droplets encapsulating yeast cells. We investigated the optimal concentration of yeast cells for droplet encapsulation. Yeast *S. cerevisiae* BY4742 was stained with Fungi-Fluor which binds to beta-linked polysaccharides found in the cell walls such as chitin and cellulose. The strained yeast cells were suspended in PBS 1.9 × 10^6^, 5.5 × 10^6^, 9.1 × 10^6^, 12.7 × 10^6^, or 16.3 × 10^6^ cells/mL concentrations. These yeast suspensions were encapsulated in droplets with a diameter of about 30 μm. Observation was carried out by using a confocal laser scanning fluorescence microscope. The images show the bright field images (BF, left column), the Fungi-Fluor fluorescence images (Fungi-Fluor, center column) and the merged images (merge, right column).

**Table S2.** Relationships between concentrations of yeast cells and the number of cells encapsulated in each droplet. After observation under a confocal microscope, the number of encapsulated yeast cells in each droplet was analyzed. In all concentrations, we observed that the proportion of droplets containing multiple yeast cells was greater than the theoretical values, probably because some yeast cells populations are dividing or aggregated.

| Concentration |  | Total | Containing  0 yeast cells | Containing  1 yeast cell | Containing  2 yeast cells | Containing  3 yeast cells | Containing  4~ yeast cells |
| --- | --- | --- | --- | --- | --- | --- | --- |
| 1.9x10^6^  cells/mL | Number of droplets | 1391 | 1336 | 42 | 8 | 5 | 0 |
|  | Ratio (%) |  | 96.04 | 3.02 | 0.58 | 0.36 | 0.00 |
|  | Theoretical ratio^※^ (%) |  | 96.08 | 3.84 | 0.08 | 0.001 | 0.000 |
| 5.5x10^6^  cells/mL | Number of droplets | 1342 | 1242 | 79 | 15 | 5 | 1 |
|  | Ratio (%) |  | 92.55 | 5.89 | 1.12 | 0.37 | 0.07 |
|  | Theoretical ratio^※^ (%) |  | 89.58 | 9.86 | 0.54 | 0.02 | 0.000 |
| 9.1x10^6^  cells/mL | Number of droplets | 1276 | 1132 | 103 | 20 | 14 | 7 |
|  | Ratio (%) |  | 88.71 | 8.07 | 1.57 | 1.10 | 0.55 |
|  | Theoretical ratio^※^ (%) |  | 85.21 | 13.63 | 1.09 | 0.06 | 0.002 |
| 12.7x10^6^  cells/mL | Number of droplets | 1540 | 1240 | 202 | 56 | 35 | 7 |
|  | Ratio (%) |  | 80.52 | 13.12 | 3.64 | 2.27 | 0.45 |
|  | Theoretical ratio^※^ (%) |  | 80.25 | 17.66 | 1.94 | 0.14 | 0.008 |
| 16.3x10^6^  cells/mL | Number of droplets | 1508 | 1156 | 220 | 71 | 41 | 20 |
|  | Ratio (%) |  | 76.66 | 14.59 | 4.71 | 2.72 | 1.33 |
|  | Theoretical ratio^※^ (%) |  | 71.89 | 23.72 | 3.91 | 0.43 | 0.04 |

^※^The theoretical ratio was calculated assuming Poisson distribution.


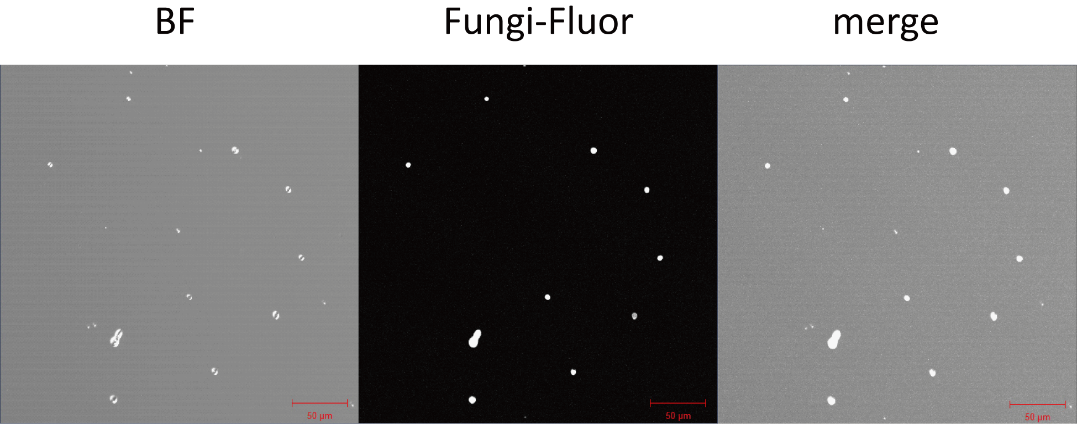
**Figure S4.** Fluorescence image of yeast stained with Fungi-Fluor. The yeast cells stained using Fungi-Fluor were suspended in PBS to prepare a yeast suspension solution of 1.9 x 10^5^ cells/mL. After that, yeast cells were observed with a confocal microscope. The images show the bright field image (BF, left column), the Fungi-Fluor fluorescence image (Fungi-Fluor, center column), and the merged image (merge, right column).

**Table S3.** The number and percentage of yeast cells which exist in single, doublet, or triplet. This result was considered to indicate that some yeast cells are dividing or aggregated.

|  | Total | singlet cell | doublet cells | triplet cells |
| --- | --- | --- | --- | --- |
| Number of yeast cells | 344 | 275 | 52 | 17 |
| Ratio (%) |  | 79.9 % | 15.1 % | 5.0 % |
