# Supplementary material for: High-throughput identification of peptide agonists against GPCRs by co-culture of mammalian reporter cells and peptide-secreting yeast cells using droplet microfluidics
Source: Sci Rep. 2019 Jul 29;9:10920. doi: 10.1038/s41598-019-47388-x (PMC6662714; doi:10.1038/s41598-019-47388-x)
Supplement: Supplementary file 2 — Supplementary Information 2 [file 41598_2019_47388_MOESM2_ESM.docx]

**TITLE**

High-throughput identification of peptide agonists against GPCRs by co-culture of mammalian reporter cells and peptide-secreting yeast cells using droplet microfluidics

**AUTHORS AND AFFILIATONS**

Kenshi Yaginuma^1^, Wataru Aoki^1,2,3^, Natsuko Miura^4^, Yuta Ohtani^1^, Shunsuke Aburaya^1,5^, Masato Kogawa^6,7^, Yohei Nishikawa^6^, Masahito Hosokawa^3,8^, Haruko Takeyama^6,7,8^, Mitsuyoshi Ueda^1,2,*^

^1^Division of Applied Life Sciences, Graduate School of Agriculture, Kyoto University, Sakyo-ku, Kyoto 606-8502, Japan

^2^JST, CREST, 7 Goban-cho, Chiyoda-ku, Tokyo 102-0076, Japan

^3^JST, PRESTO, 7 Goban-cho, Chiyoda-ku, Tokyo 102-0076, Japan

^4^Graduate School of Life and Environmental Sciences, Osaka Prefecture University, 1-1 Gakuen-cho, Naka-ku, Sakai, Osaka 599-8531, Japan

^5^Japan Society for the Promotion of Science, 5-3-1 Kojimachi, Chiyoda-ku, Tokyo 102-0083, Japan

^6^Department of Life Science & Medical Bioscience, School of Advanced Science and Engineering, Waseda University, Shinjuku-ku, Tokyo 169-8555, Japan

^7^Computational Bio Big-Data Open Innovation Laboratory, AIST-Waseda University, 3-4-1 Okubo, Shinjuku-ku, Tokyo, 169–0072, Japan

^8^Institute for Advanced Research of Biosystem Dynamics, Waseda Research Institute for Science and Engineering, Waseda University, Shinjuku-ku, Tokyo 169-8555, Japan

*Correspondence should be addressed to: Mitsuyoshi Ueda

Tel.: +81-75-753-6495; Fax: +81-75-753-6112; E-mail: miueda@kais.kyoto-u.ac.jp


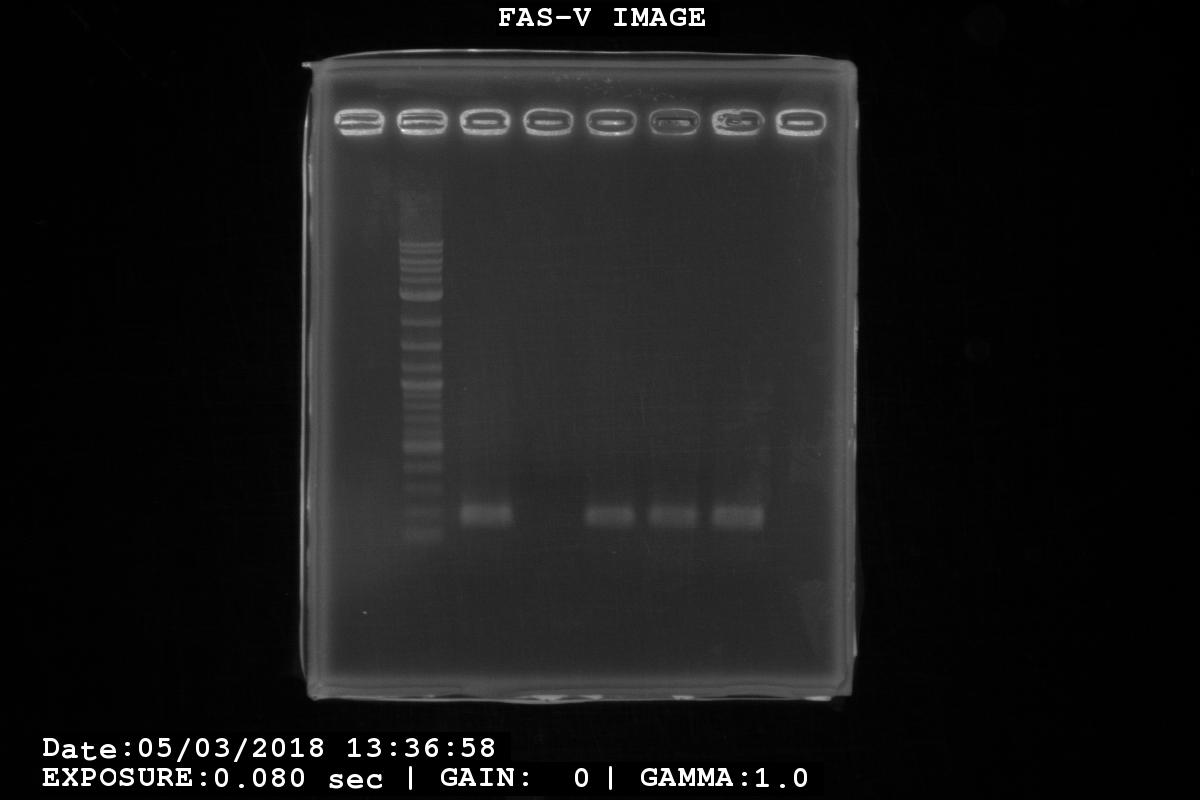


Full-length gel image of Figure 4e
