# Supplementary material for: High-throughput identification of peptide agonists against GPCRs by co-culture of mammalian reporter cells and peptide-secreting yeast cells using droplet microfluidics
Source: Sci Rep. 2019 Jul 29;9:10920. doi: 10.1038/s41598-019-47388-x (PMC6662714; doi:10.1038/s41598-019-47388-x)

TITLE

High-throughput identification of peptide agonists against GPCRs by co-culture of mammalian reporter cells and peptide-secreting yeast cells using droplet microfluidics

AUTHORS AND AFFILIATONS

Kenshi Yaginuma[1], Wataru Aoki[1,2,3], Natsuko Miura[4], Yuta Ohtani[1], Shunsuke Aburaya[1,5], Masato Kogawa[6,7], Yohei Nishikawa[6], Masahito Hosokawa[3,8], Haruko Takeyama[6,7,8], Mitsuyoshi Ueda[1,2,*]

[1]Division of Applied Life Sciences, Graduate School of Agriculture, Kyoto University, Sakyo-ku, Kyoto 606-8502, Japan

[2]JST, CREST, 7 Goban-cho, Chiyoda-ku, Tokyo 102-0076, Japan

[3]JST, PRESTO, 7 Goban-cho, Chiyoda-ku, Tokyo 102-0076, Japan

[4]Graduate School of Life and Environmental Sciences, Osaka Prefecture University, 1-1 Gakuen-cho, Naka-ku, Sakai, Osaka 599-8531, Japan

[5]Japan Society for the Promotion of Science, 5-3-1 Kojimachi, Chiyoda-ku, Tokyo 102-0083, Japan

[6]Department of Life Science & Medical Bioscience, School of Advanced Science and Engineering, Waseda University, Shinjuku-ku, Tokyo 169-8555, Japan

[7]Computational Bio Big-Data Open Innovation Laboratory, AIST-Waseda University, 3-4-1 Okubo, Shinjuku-ku, Tokyo, 169–0072, Japan

[8]Institute for Advanced Research of Biosystem Dynamics, Waseda Research Institute for Science and Engineering, Waseda University, Shinjuku-ku, Tokyo 169-8555, Japan

*Correspondence should be addressed to: Mitsuyoshi Ueda

Tel.: +81-75-753-6495; Fax: +81-75-753-6112; E-mail: miueda@kais.kyoto-u.ac.jp

pIRES-hGLP1R (6488 bp)

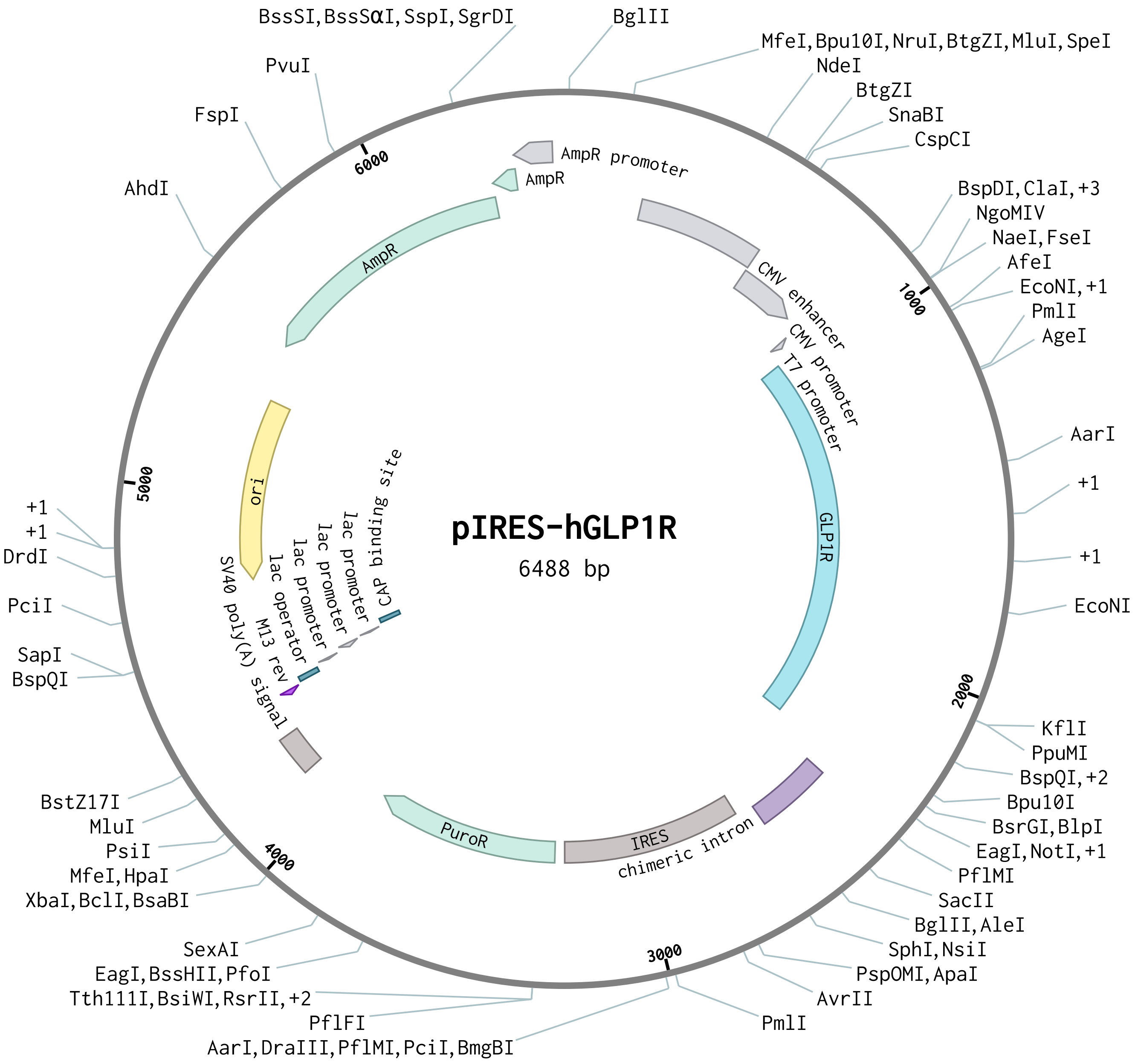

pIRES-hGLP1R (6488 bp)

BglII

gacggatcgggagatctcccgatcccctatggtcgactctcagtacaatctgctctgatgccgcatagttaagccagtatctgctccctgcttgtgtgttggaggtc
ctgcctagccctctagagggctaggggataccagctgagagtcatgttagacgagactacggcgtatcaattcggtcatagacgagggacgaacacacaacctccag

20 40 60 80 100

MfeI Bpu10I NruI

gctgagtagtgcgcgagcaaaatttaagctacaacaaggcaaggcttgaccgacaattgcatgaagaatctgcttagggttaggcgttttgcgctgcttcgcgatgt
cgactcatcacgcgctcgttttaaattcgatgttgttccgttccgaactggctgttaacgtacttcttagacgaatcccaatccgcaaaacgcgacgaagcgctaca

120 140 160 180 200

MluI
BtgZI SpeI

acgggccagatatacgcgttgacattgattattgactagttattaatagtaatcaattacggggtcattagttcatagcccatatatggagttccgcgttacataac
tgcccggtctatatgcgcaactgtaactaataactgatcaataattatcattagttaatgccccagtaatcaagtatcgggtatatacctcaaggcgcaatgtattg

CMV enhancer

220 240 260 280 300 320

ttacggtaaatggcccgcctggctgaccgcccaacgacccccgcccattgacgtcaataatgacgtatgttcccatagtaacgccaatagggactttccattgacgt
aatgccatttaccgggcggaccgactggcgggttgctgggggcgggtaactgcagttattactgcatacaagggtatcattgcggttatccctgaaaggtaactgca

CMV enhancer

340 360 380 400 420

NdeI

caatgggtggactatttacggtaaactgcccacttggcagtacatcaagtgtatcatatgccaagtacgccccctattgacgtcaatgacggtaaatggcccgcctg
gttacccacctgataaatgccatttgacgggtgaaccgtcatgtagttcacatagtatacggttcatgcgggggataactgcagttactgccatttaccgggcggac

CMV enhancer

440 460 480 500 520

SnaBI
BtgZI CspCI

gcattatgcccagtacatgaccttatgggactttcctacttggcagtacatctacgtattagtcatcgctattaccatggtgatgcggttttggcagtacatcaatg
cgtaatacgggtcatgtactggaataccctgaaaggatgaaccgtcatgtagatgcataatcagtagcgataatggtaccactacgccaaaaccgtcatgtagttac

CMV enhancer | CMV promoter

540 560 580 600 620 640

ggcgtggatagcggtttgactcacggggatttccaagtctccaccccattgacgtcaatgggagtttgttttggcaccaaaatcaacgggactttccaaaatgtcgt
ccgcacctatcgccaaactgagtgcccctaaaggttcagaggtggggtaactgcagttaccctcaaacaaaaccgtggttttagttgccctgaaaggttttacagca

CMV promoter

660 680 700 720 740

```
aacaactccgccccattgacgcaaatgggcggtaggcgtgtacggtgggaggtctatataagcagagctctctggctaactagagaacccactgcttactggcttat
ttgttgaggcggggtaactgcgtttacccgccatccgcacatgccaccctccagatatattcgtctcgagagaccgattgatctcttgggtgacgaatgaccgaata
CMV promoter
760 780 800 820 840
```

```
PvuI
BspDI
ClaI
NaeI
NgoMIV
cgaaattaatacgactcactatagggagacccaagcttggtacCGAGCTCGGATCGATCGCCACCATGGCCGGCGCCCCCGGCCCGCTGCGCCTTGCGCTGCTGCTG
gctttaattatgctgagtgatatccctctgggttcgaaccatgGCTCGAGCCTAGCTAGCGGTGGTACCGGCCGCGGGGGCCGGGCGACGCGGAACGCGACGACGAC
T7 promoter
GLP1R
860 880 900 920 940 960
```

```
FseI
NaeI
NgoMIV
AfeI
CTCGGGATGGTGGGCAGGGCCGGCCCCCGCCCCCAGGGTGCCACTGTGTCCCTCTGGGAGACGGTGCAGAAATGGCGAGAATACCGACGCCAGTGCCAGCGCTCCCT
GAGCCCTACCACCCGTCCCGGCCGGGGGCGGGGGTCCCACGGTGACACAGGGAGACCCTCTGCCACGTCTTTACCGCTCTTATGGCTGCGGTCACGGTCGCGAGGGA
GLP1R
980 1,000 1,020 1,040 1,060
```

```
BamHI
EcoNI
GACTGAGGATCCACCTCCTGCCACAGACTTGTTCTGCAACCGGACCTTCGATGAATACGCCTGCTGGCCAGATGGGGAGCCAGGCTCGTTCGTGAATGTCAGCTGCC
CTGACTCCTAGGTGGAGGACGGTGTCTGAACAAGACGTTGGCCTGGAAGCTACTTATGCGGACGACCGGTCTACCCCTCGGTCCGAGCAAGCACTTACAGTCGACGG
GLP1R
1,080 1,100 1,120 1,140 1,160
```

```
AgeI
PmlI
CCTGGTACCTGCCCTGGGCCAGCAGTGTGCCGCAGGGCCACGTGTACCGGTTCTGCACAGCTGAAGGCCTCTGGCTGCAGAAGGACAACTCCAGCCTGCCCTGGAGG
GGACCATGGACGGGACCCGGTCGTCACACGGCGTCCCGGTGCACATGGCCAAGACGTGTCGACTTCCGGAGACCGACGTCTTCCTGTTGAGGTCGGACGGGACCTCC
GLP1R
1,180 1,200 1,220 1,240 1,260 1,280
```

```
GACTTGTCGGAGTGCGAGGAGTCCAAGCGAGGGGAGAGAAGCTCCCCGGAGGAGCAGCTCCTGTTCCTCTACATCATCTACACGGTGGGCTACGCACTCTCCTTCTC
CTGAACAGCCTCACGCTCCTCAGGTTCGCTCCCCTCTCTTCGAGGGGCCTCCTCGTCGAGGACAAGGAGATGTAGTAGATGTGCCACCCGATGCGTGAGAGGAAGAG
GLP1R
1,300 1,320 1,340 1,360 1,380
```

AarI

TGCTCTGGTTATCGCCTCTGCGATCCTCCTCGGCTTCAGACACCTGCACTGCACCCGGAACTACATCCACCTGAACCTGTTTGCATCCTTCATCCTGCGAGCATTGT
ACGAGACCAATAGCGGAGACGCTAGGAGGAGCCGAAGTCTGTGGACGTGACGTGGGCCTTGATGTAGGTGGACTTGGACAAACGTAGGAAGTAGGACGCTCGTAACA

GLP1R

1,400 1,420 1,440 1,460 1,480

BstXI

CCGTCTTCATCAAGGACGCAGCCCTGAAGTGGATGTATAGCACAGCCGCCCAGCAGCACCAGTGGGATGGGCTCCTCTCCTACCAGGACTCTCTGAGCTGCCGCCTG
GGCAGAAGTAGTTCCTGCGTCGGGACTTCACCTACATATCGTGTCGGCGGGTCGTCGTGGTCACCCTACCCGAGGAGAGGATGGTCCTGAGAGACTCGACGGCGGAC

GLP1R

1,500 1,520 1,540 1,560 1,580 1,600

BsrGI

GTGTTTCTGCTCATGCAGTACTGTGTGGCGGCCAATTACTACTGGCTCTTGGTGGAGGGCGTGTACCTGTACACACTGCTGGCCTTCTCGGTCTTCTCTGAGCAATG
CACAAAGACGAGTACGTCATGACACACCGCCGGTTAATGATGACCGAGAACCACCTCCCGCACATGGACATGTGTGACGACCGGAAGAGCCAGAAGAGACTCGTTAC

GLP1R

1,620 1,640 1,660 1,680 1,700

EcoNI

GATCTTCAGGCTCTACGTGAGCATAGGCTGGGGTGTTCCCCTGCTGTTTGTTGTCCCCTGGGGCATTGTCAAGTACCTCTATGAGGACGAGGGCTGCTGGACCAGGA
CTAGAAGTCCGAGATGCACTCGTATCCGACCCCACAAGGGGACGACAAACAACAGGGGACCCCGTAACAGTTCATGGAGATACTCCTGCTCCCGACGACCTGGTCCT

GLP1R

1,720 1,740 1,760 1,780 1,800

ACTCCAACATGAACTACTGGCTCATTATCCGGCTGCCCATTCTCTTTGCCATTGGGGTGAACTTCCTCATCTTTGTTCGGGTCATCTGCATCGTGGTATCCAAACTG
TGAGGTTGTACTTGATGACCGAGTAATAGGCCGACGGGTAAGAGAAACGGTAACCCCACTTGAAGGAGTAGAAACAAGCCCAGTAGACGTAGCACCATAGGTTTGAC

GLP1R

1,820 1,840 1,860 1,880 1,900 1,920

AAGGCCAATCTCATGTGCAAGACAGACATCAAATGCAGACTTGCCAAGTCCACGCTGACACTCATCCCCCTGCTGGGGACTCATGAGGTCATCTTTGCCTTTGTGAT
TTCCGGTTAGAGTACACGTTCTGTCTGTAGTTTACGTCTGAACGGTTCAGGTGCGACTGTGAGTAGGGGGACGACCCCTGAGTACTCCAGTAGAAACGGAAACACTA

GLP1R

1,940 1,960 1,980 2,000 2,020

PpuMI
KflI

GGACGAGCACGCCCGGGGGACCCTGCGCTTCATCAAGCTGTTTACAGAGCTCTCCTTCACCTCCTTCCAGGGGCTGATGGTGGCCATCTTATACTGCTTTGTCAACA
CCTGCTCGTGCGGGCCCCCTGGGACGCGAAGTAGTTCGACAAATGTCTCGAGAGGAAGTGGAGGAAGGTCCCCGACTACCACCGGTAGAATATGACGAAACAGTTGT

GLP1R

2,040 2,060 2,080 2,100 2,120 2,140

BspQI
SapI
AfeI

ATGAGGTCCAGCTGGAATTTCGGAAGAGCTGGGAGCGCTGGCGGCTTGAGCACTTGCACATCCAGAGGGACAGCAGCATGAAGCCCCTCAAGTGTCCCACCAGCAGC
TACTCCAGGTCGACCTTAAAGCCTTCTCGACCCTCGCGACCGCCGAACTCGTGAACGTGTAGGTCTCCCTGTCGTCGTACTTCGGGGAGTTCACAGGGTGGTCGTCG

GLP1R

2,160 2,180 2,200 2,220 2,240

NotI
EagI
Bpu10I BsrGI BlpI BstXI

CTGAGCAGTGGAGCCACGGCGGGCAGCAGCATGTACACAGCCACTTGCCAGGCCTCCTGCAGCTGAgcggccgcatagataactgatccagtgtgctggaattaatt
GACTCGTCACCTCGGTGCCGCCCGTCGTCGTACATGTGTCGGTGAACGGTCCGGAGGACGTCGACTcgccggcgtatctattgactaggtcacacgaccttaattaa

GLP1R

2,260 2,280 2,300 2,320 2,340

PflMI

cgctgtctgcgagggccagctgttggggtgagtactccctctcaaaagcgggcatgacttctgcgctaagattgtcagtttccaaaaacgaggaggatttgatattc
gcgacagacgctcccggtcgacaaccccactcatgagggagagttttcgcccgtactgaagacgcgattctaacagtcaaaggtttttgctcctcctaaactataag

chimeric intron

2,360 2,380 2,400 2,420 2,440 2,460

SacII BglII

acctggcccgcggtgatgcctttgagggtggccgcgtccatctggtcagaaaagacaatctttttgttgtcaagcttgaggtgtggcaggcttgagatctggccata
tggaccgggcgccactacggaaactcccaccggcgcaggtagaccagtcttttctgttagaaaaacaacagttcgaactccacaccgtccgaactctagaccggtat

chimeric intron

2,480 2,500 2,520 2,540 2,560

NsiI
AleI SphI

cacttgagtgacaatgacatccactttgcctttctctccacaggtgtccactcccaggtccaactgcaggtcgagcatgcatctagggcggccaattccgcccctct
gtgaactcactgttactgtaggtgaaacggaaagagaggtgtccacaggtgagggtccaggttgacgtccagctcgtacgtagatcccgccggttaaggcggggaga

chimeric intron

2,580 2,600 2,620 2,640 2,660

ccctcccccccccctaacgttactggccgaagccgcttggaataaggccggtgtgcgtttgtctatatgtgattttccaccatattgccgtcttttggcaatgtgag
gggaggggggggggattgcaatgaccggcttcggcgaaccttattccggccacacgcaaacagatatacactaaaaggtggtataacggcagaaaaccgttacactc

IRES

2,680 2,700 2,720 2,740 2,760 2,780

ApaI
PspOMI AvrII

ggcccggaaacctggccctgtcttcttgacgagcattcctaggggtctttcccctctcgccaaaggaatgcaaggtctgttgaatgtcgtgaaggaagcagttcctc
ccgggcctttggaccgggacagaagaactgctcgtaaggatccccagaaaggggagagcggtttccttacgttccagacaacttacagcacttccttcgtcaaggag

IRES

2,800 2,820 2,840 2,860 2,880

PmlI

tggaagcttcttgaagacaaacaacgtctgtagcgaccctttgcaggcagcggaaccccccacctggcgacaggtgcctctgcggccaaaagccacgtgtataagat
accttcgaagaacttctgtttgttgcagacatcgctgggaaacgtccgtcgccttggggggtggaccgctgtccacggagacgccggttttcggtgcacatattcta

IRES

2,900 2,920 2,940 2,960 2,980

AarI DraIII

acacctgcaaaggcggcacaaccccagtgccacgttgtgagttggatagttgtggaaagagtcaaatggctctcctcaagcgtattcaacaaggggctgaaggatgc
tgtggacgtttccgccgtgttggggtcacggtgcaacactcaacctatcaacacctttctcagtttaccgagaggagttcgcataagttgttccccgacttcctacg

IRES

3,000 3,020 3,040 3,060 3,080 3,100

PflMI PciI

ccagaaggtaccccattgtatgggatctgatctggggcctcggtgcacatgctttacatgtgtttagtcgaggttaaaaaaacgtctaggccccccgaaccacgggg
ggtcttccatggggtaacataccctagactagaccccggagccacgtgtacgaaatgtacacaaatcagctccaatttttttgcagatccggggggcttggtgcccc

IRES

3,120 3,140 3,160 3,180 3,200

BmgBI

acgtggttttcctttgaaaaacacgatgataagcttgccacaacccacaaggagacgaccttccatgaccgagtacaagcccacggtgcgcctcgccacccgcgacg
tgcaccaaaaggaaactttttgtgctactattcgaacggtgttgggtgttcctctgctggaaggtactggctcatgttcgggtgccacgcggagcggtgggcgctgc

IRES PuroR

3,220 3,240 3,260 3,280 3,300

Tth111I
PflFI BsiWI RsrII BstEII

acgtcccccgggccgtacgcaccctcgccgccgcgttcgccgactaccccgccacgcgccacaccgtcgacccggaccgccacatcgagcgggtcaccgagctgcaa
tgcagggggcccggcatgcgtgggagcggcggcgcaagcggctgatggggcggtgcgcggtgtggcagctgggcctggcggtgtagctcgcccagtggctcgacgtt

PuroR

3,320 3,340 3,360 3,380 3,400 3,420

SacII

gaactcttcctcacgcgcgtcgggctcgacatcggcaaggtgtgggtcgcggacgacggcgccgcggtggcggtctggaccacgccggagagcgtcgaagcgggggc
cttgagaaggagtgcgcgcagcccgagctgtagccgttccacacccagcgcctgctgccgcggcgccaccgccagacctggtgcggcctctcgcagcttcgcccccg

PuroR

3,440 3,460 3,480 3,500 3,520

ggtgttcgccgagatcggcccgcgcatggccgagttgagcggttcccggctggccgcgcagcaacagatggaaggcctcctggcgccgcaccggcccaaggagcccg
ccacaagcggctctagccgggcgcgtaccggctcaactcgccaagggccgaccggcgcgtcgttgtctaccttccggaggaccgcggcgtggccgggttcctcgggc

PuroR

3,540 3,560 3,580 3,600 3,620

EagI BssHII

cgtggttcctggccaccgtcggcgtctcgcccgaccaccagggcaagggtctgggcagcgccgtcgtgctccccggagtggaggcggccgagcgcgccggggtgccc
gcaccaaggaccggtggcagccgcagagcgggctggtggtcccgttcccagacccgtcgcggcagcacgaggggcctcacctccgccggctcgcgcggccccacggg

PuroR

3,640 3,660 3,680 3,700 3,720 3,740

PfoI SexAI

gccttcctggagacctccgcgccccgcaacctccccttctacgagcggctcggcttcaccgtcaccgccgacgtcgagtgcccgaaggaccgcgcgacctggtgcat
cggaaggacctctggaggcgcggggcgttggaggggaagatgctcgccgagccgaagtggcagtggcggctgcagctcacgggcttcctggcgcgctggaccacgta

PuroR

3,760 3,780 3,800 3,820 3,840

gacccgcaagcccggtgcctgacgcccgccccacgacccgcagcgcccgaccgaaaggagcgcacgaccccatggctccgaccgaagccgacccgggcggccccgcc
ctgggcgttcgggccacggactgcgggcggggtgctgggcgtcgcgggctggctttcctcgcgtgctggggtaccgaggctggcttcggctgggcccgccggggcgg

PuroR

3,860 3,880 3,900 3,920 3,940

BsaBI

XbaI BclI

gaccccgcacccgcccccgaggcccaccgactctagataactgatcataatcagccataccacatttgtagaggttttacttgctttaaaaaacctcccacacctcc
ctggggcgtgggcgggggctccgggtggctgagatctattgactagtattagtcggtatggtgtaaacatctccaaaatgaacgaaattttttggagggtgtggagg

3,960 3,980 4,000 4,020 4,040 4,060

MfeI HpaI PsiI

ccctgaacctgaaacataaaatgaatgcaattgttgttgttaacttgtttattgcagcttataatggttacaaataaagcaatagcatcacaaatttcacaaataaa
gggacttggactttgtattttacttacgttaacaacaacaattgaacaaataacgtcgaatattaccaatgtttatttcgttatcgtagtgtttaaagtgtttattt

SV40 poly(A) signal

4,080 4,100 4,120 4,140 4,160

MluI

gcatttttttcactgcattctagttgtggtttgtccaaactcatcaatgtatcttaacgcgtcgagtgcattctagttgtggtttgtccaaactcatcaatgtatct
cgtaaaaaaagtgacgtaagatcaacaccaaacaggtttgagtagttacatagaattgcgcagctcacgtaagatcaacaccaaacaggtttgagtagttacataga

SV40 poly(A) signal

4,180 4,200 4,220 4,240 4,260 4,280

BstZ17I

tatcatgtctgtataccgtcgacctctagctagagcttggcgtaatcatggtcatagctgtttcctgtgtgaaattgttatccgctcacaattccacacaacatacg
atagtacagacatatggcagctggagatcgatctcgaaccgcattagtaccagtatcgacaaaggacacactttaacaataggcgagtgttaaggtgtgttgtatgc

M13 rev
lac operator

4,300 4,320 4,340 4,360 4,380

agccggaagcataaagtgtaaagcctggggtgcctaatgagtgagctaactcacattaattgcgttgcgctcactgcccgctttccagtcgggaaacctgtcgtgcc
tcggccttcgtatttcacatttcggaccccacggattactcactcgattgagtgtaattaacgcaacgcgagtgacgggcgaaaggtcagccctttggacagcacgg

CAP binding site

4,400 4,420 4,440 4,460 4,480

BspQI
SapI

agctgcattaatgaatcggccaacgcgcggggagaggcggtttgcgtattgggcgctcttccgcttcctcgctcactgactcgctgcgctcggtcgttcggctgcgg
tcgacgtaattacttagccggttgcgcgcccctctccgccaaacgcataacccgcgagaaggcgaaggagcgagtgactgagcgacgcgagccagcaagccgacgcc

4,500 4,520 4,540 4,560 4,580 4,600

PciI

cgagcggtatcagctcactcaaaggcggtaatacggttatccacagaatcaggggataacgcaggaaagaacatgtgagcaaaaggccagcaaaaggccaggaaccg
gctcgccatagtcgagtgagtttccgccattatgccaataggtgtcttagtcccctattgcgtcctttcttgtacactcgttttccggtcgttttccggtccttggc

4,620 4,640 4,660 4,680 4,700

DrdI

taaaaaggccgcgttgctggcgtttttccataggctccgcccccctgacgagcatcacaaaaatcgacgctcaagtcagaggtggcgaaacccgacaggactataaa
atttttccggcgcaacgaccgcaaaaaggtatccgaggcggggggactgctcgtagtgtttttagctgcgagttcagtctccaccgctttgggctgtcctgatattt

ori

4,720 4,740 4,760 4,780 4,800

BssSαI
BssSI

gataccaggcgtttccccctggaagctccctcgtgcgctctcctgttccgaccctgccgcttaccggatacctgtccgcctttctcccttcgggaagcgtggcgctt
ctatggtccgcaaagggggaccttcgagggagcacgcgagaggacaaggctgggacggcgaatggcctatggacaggcggaaagagggaagcccttcgcaccgcgaa

ori

4,820 4,840 4,860 4,880 4,900 4,920

tctcaatgctcacgctgtaggtatctcagttcggtgtaggtcgttcgctccaagctgggctgtgtgcacgaaccccccgttcagcccgaccgctgcgccttatccgg
agagttacgagtgcgacatccatagagtcaagccacatccagcaagcgaggttcgacccgacacacgtgcttggggggcaagtcgggctggcgacgcggaataggcc

ori

4,940 4,960 4,980 5,000 5,020

taactatcgtcttgagtccaacccggtaagacacgacttatcgccactggcagcagccactggtaacaggattagcagagcgaggtatgtaggcggtgctacagagt
attgatagcagaactcaggttgggccattctgtgctgaatagcggtgaccgtcgtcggtgaccattgtcctaatcgtctcgctccatacatccgccacgatgtctca

ori

5,040 5,060 5,080 5,100 5,120

tcttgaagtggtggcctaactacggctacactagaaggacagtatttggtatctgcgctctgctgaagccagttaccttcggaaaaagagttggtagctcttgatcc
agaacttcaccaccggattgatgccgatgtgatcttcctgtcataaaccatagacgcgagacgacttcggtcaatggaagcctttttctcaaccatcgagaactagg

ori

5,140 5,160 5,180 5,200 5,220 5,240

ggcaaacaaaccaccgctggtagcggtggtttttttgtttgcaagcagcagattacgcgcagaaaaaaaggatctcaagaagatcctttgatcttttctacggggtc
ccgtttgtttggtggcgaccatcgccaccaaaaaaacaaacgttcgtcgtctaatgcgcgtctttttttcctagagttcttctaggaaactagaaaagatgccccag

ori

5,260 5,280 5,300 5,320 5,340

tgacgctcagtggaacgaaaactcacgttaagggattttggtcatgagattatcaaaaaggatcttcacctagatccttttaaattaaaaatgaagttttaaatcaa
actgcgagtcaccttgcttttgagtgcaattccctaaaaccagtactctaatagtttttcctagaagtggatctaggaaaatttaatttttacttcaaaatttagtt

5,360 5,380 5,400 5,420 5,440

tctaaagtatatatgagtaaacttggtctgacagttaccaatgcttaatcagtgaggcacctatctcagcgatctgtctatttcgttcatccatagttgcctgactc
agatttcatatatactcatttgaaccagactgtcaatggttacgaattagtcactccgtggatagagtcgctagacagataaagcaagtaggtatcaacggactgag

AmpR

5,460 5,480 5,500 5,520 5,540 5,560

AhdI

cccgtcgtgtagataactacgatacgggagggcttaccatctggccccagtgctgcaatgataccgcgagacccacgctcaccggctccagatttatcagcaataaa
gggcagcacatctattgatgctatgccctcccgaatggtagaccggggtcacgacgttactatggcgctctgggtgcgagtggccgaggtctaaatagtcgttattt

AmpR

5,580 5,600 5,620 5,640 5,660

ccagccagccggaagggccgagcgcagaagtggtcctgcaactttatccgcctccatccagtctattaattgttgccgggaagctagagtaagtagttcgccagtta
ggtcggtcggccttcccggctcgcgtcttcaccaggacgttgaaataggcggaggtaggtcagataattaacaacggcccttcgatctcattcatcaagcggtcaat

AmpR

5,680 5,700 5,720 5,740 5,760

FspI

atagtttgcgcaacgttgttgccattgctacaggcatcgtggtgtcacgctcgtcgtttggtatggcttcattcagctccggttcccaacgatcaaggcgagttaca
tatcaaacgcgttgcaacaacggtaacgatgtccgtagcaccacagtgcgagcagcaaaccataccgaagtaagtcgaggccaagggttgctagttccgctcaatgt

AmpR

5,780 5,800 5,820 5,840 5,860 5,880

PvuI

tgatcccccatgttgtgcaaaaaagcggttagctccttcggtcctccgatcgttgtcagaagtaagttggccgcagtgttatcactcatggttatggcagcactgca
actagggggtacaacacgttttttcgccaatcgaggaagccaggaggctagcaacagtcttcattcaaccggcgtcacaatagtgagtaccaataccgtcgtgacgt

AmpR

5,900 5,920 5,940 5,960 5,980

taattctcttactgtcatgccatccgtaagatgcttttctgtgactggtgagtactcaaccaagtcattctgagaatagtgtatgcggcgaccgagttgctcttgcc
attaagagaatgacagtacggtaggcattctacgaaaagacactgaccactcatgagttggttcagtaagactcttatcacatacgccgctggctcaacgagaacgg

AmpR

6,000 6,020 6,040 6,060 6,080

cggcgtcaatacgggataataccgcgccacatagcagaactttaaaagtgctcatcattggaaaacgttcttcggggcgaaaactctcaaggatcttaccgctgttg
gccgcagttatgccctattatggcgcggtgtatcgtcttgaaattttcacgagtagtaaccttttgcaagaagccccgcttttgagagttcctagaatggcgacaac

AmpR

6,100 6,120 6,140 6,160 6,180 6,200

BssSαI
BssSI

agatccagttcgatgtaacccactcgtgcacccaactgatcttcagcatcttttactttcaccagcgtttctgggtgagcaaaaacaggaaggcaaaatgccgcaaa
tctaggtcaagctacattgggtgagcacgtgggttgactagaagtcgtagaaaatgaaagtggtcgcaaagacccactcgtttttgtccttccgttttacggcgttt

AmpR AmpR

6,220 6,240 6,260 6,280 6,300

SspI

aaagggaataagggcgacacggaaatgttgaatactcatactcttcctttttcaatattattgaagcatttatcagggttattgtctcatgagcggatacatatttg
tttcccttattcccgctgtgcctttacaacttatgagtatgagaaggaaaaagttataataacttcgtaaatagtcccaataacagagtactcgcctatgtataaac

AmpR AmpR promoter

6,320 6,340 6,360 6,380 6,400 6,420

SgrDI

aatgtatttagaaaaataaacaaataggggttccgcgcacatttccccgaaaagtgccacctgacgtc
ttacataaatctttttatttgtttatccccaaggcgcgtgtaaaggggcttttcacggtggactgcag

AmpR promoter

6,430 6,440 6,450 6,460 6,470 6,480

pCRE-NLuc (partial sequence) (1419 bp)

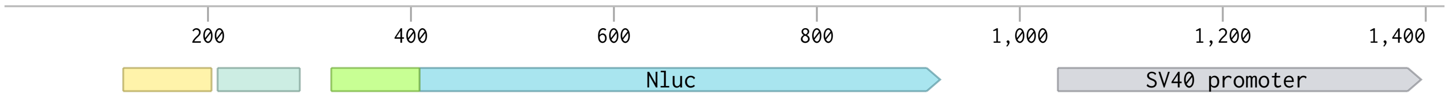

pCRE-NLuc (partial sequence) (1419 bp)

```
BsaWI
AgeI                    BstYI
TACGGTAGCATACCGGTACNNNNACCAGACANNNACGTCAGCTGCCAGATCCCATGGCCGTCATACTGTGACGTCTTTCAGACACCCCATTGACGTCAATGGGAGAA
ATGCCATCGTATGGCCATGNNNNTGGTCTGTNNNTGCAGTCGACGGTCTAGGGTACCGGCAGTATGACACTGCAGAAAGTCTGTGGGGTAACTGCAGTTACCCTCTT
20 40 60 80 100
```

```
Bsp1286I
BsiHKAI
BaeGI                                                BstBI
ApaLI                                                EcoRI
NciI BsaAI          BstYI                            ApoI
CCCGGTACGTGCACCAGACAGTGACGTCAGCTGCCAGATCCCATGGCCGTCATACTGTGACGTCTTTCAGACACCCCATTGACGTCAATGGGAGAACGAATTCGAAC
GGGCCATGCACGTGGTCTGTCACTGCAGTCGACGGTCTAGGGTACCGGCAGTATGACACTGCAGAAAGTCTGTGGGGTAACTGCAGTTACCCTCTTGCTTAGCTTG
CRE
120 140 160 180 200
```

```
PaeR7I
PspXI
BsoBI
XhoI                                                 AloI
TliI                                                 BsaXI
SmlI                                                 KpnI
BstUI                                                Acc65I
RsrII    AflIII     PleI     AvaI                    BanI
MslI BstUI    MluI HgaI MlyI HinfI
ACGCAGATGCAGTCGGGGCGGCGCGGTCCGAGGTCCACTTCGCATATTAAGGTGACGCGTGTGGCCTCGAACACCGACTCGAGCCGCTTAAAAGTTGGTACCGCCAC
TGCGTCTACGTCAGCCCCGCCGCGCCAGGCTCCAGGTGAAGCGTATAATTCCACTGCGCACACCGGAGCTTGTGGCTGAGCTCGGCGAATTTTCAACCATGGCGGTG
TK Minimal promoter
220 240 260 280 300 320
```

```
BbsI
HaeII   PasI   BmrI   AjuI
CATGAACTCCTTCTCCACAAGCGCCTTCGGTCCAGTTGCCTTCTCCCTGGGCCTGCTCCTGGTGTTGCCTGCTGCCTTCCCTGCCCCAGTCTTCACACTCGAAGATT
GTACTTGAGGAAGAGGTGTTCGCGGAAGCCAGGTCAACGGAAGAGGGACCCGGACGAGGACCACAACGGACGACGGAAGGGACGGGGTCAGAAGTGTGAGCTTCTAA
IL6-ss Nluc
340 360 380 400 420
```

```
BsoBI
AvaI
NaeI   Tth111I   HinfI
NgoMIV PflFI EcoNI TfiI
TCGTTGGGGACTGGCGACAGACAGCCGGCTACAACCTGGACCAAGTCCTTGAACAGGGAGGTGTGTCCAGTTTGTTTCAGAATCTCGGGGTGTCCGTAACTCCGATC
AGCAACCCCTGACCGCTGTCTGTCGGCCGATGTTGGACCTGGTTCAGGAACTTGTCCCTCCACACAGGTCAAACAAAGTCTTAGAGCCCCACAGGCATTGAGGCTAG
Nluc
440 460 480 500 520
```

BsrBI
Bpu10I
Hpy188III BsaBI AcuI BsrBI ApoI

CAAAGGATTGTCCTGAGCGGTGAAAATGGGCTGAAGATCGACATCCATGTCATCATCCCGTATGAAGGTCTGAGCGGCGACCAAATGGGCCAGATCGAAAAAATTTT
GTTTCCTAACAGGACTCGCCACTTTTACCCGACTTCTAGCTGTAGGTACAGTAGTAGGGCATACTTCCAGACTCGCCGCTGGTTTACCCGGTCTAGCTTTTTTAAAA

Nluc

540 560 580 600 620 640

BsaBI EagI
BclI BsgI BtgZI

TAAGGTGGTGTACCCTGTGGATGATCATCACTTTAAGGTGATCCTGCACTATGGCACACTGGTAATCGACGGGGTTACGCCGAACATGATCGACTATTTCGGACGGC
ATTCCACCACATGGGACACCTACTAGTAGTGAAATTCCACTAGGACGTGATACCGTGTGACCATTAGCTGCCCCAATGCGGCTTGTACTAGCTGATAAAGCCTGCCG

Nluc

660 680 700 720 740

EcoO109I
PpuMI BclI
BsiEI MslI KflI HaeII

CGTATGAAGGCATCGCCGTGTTCGACGGCAAAAAGATCACTGTAACAGGGACCCTGTGGAACGGCAACAAAATTATCGACGAGCGCCTGATCAACCCCGACGGCTCC
GCATACTTCCGTAGCGGCACAAGCTGCCGTTTTTCTAGTGACATTGTCCCTGGGACACCTTGCCGTTGTTTTAATAGCTGCTCGCGGACTAGTTGGGGCTGCCGAGG

Nluc

760 780 800 820 840

FspAI
FspI
BsmBI
BsmAI BfaI
BccI BsmI BcoDI BsiWI

CTGCTGTTCCGAGTAACCATCAACGGAGTGACCGGCTGGCGGCTGTGCGAACGCATTCTGGCGTAATGCGCATGAGACGTACGCTAGAAATAATTCTTACTGTCATG
GACGACAAGGCTCATTGGTAGTTGCCTCACTGGCCGACCGCCGACACGCTTGCGTAAGACCGCATTACGCGTACTCTGCATGCGATCTTTATTAAGAATGACAGTAC

Nluc

860 880 900 920 940 960

CCAAGTAAGATGCTTTTCTGGCGCAGCACCATGGCCTGAAATAACCTCTGAAAGAGGAACTTGGTTAGCTACCTTCTGAGGCGGAAAGAACCAGCTGTGGAATGTGT
GGTTCATTCTACGAAAAGACCGCGTCGTGGTACCGGACTTTATTGGAGACTTTCTCCTTGAACCAATCGATGGAAGACTCCGCCTTTCTTGGTCGACACCTTACACA

SV40 promoter

980 1,000 1,020 1,040 1,060

NsiI
SphI
NspI
BseYI BstAPI SexAI BseYI

```
GTCAGTTAGGGTGTGGAAAGTCCCCAGGCTCCCCAGCAGGCAGAAGTATGCAAAGCATGCATCTCAATTAGTCAGCAACCAGGTGTGGAAAGTCCCCAGGCTCCCCA
CAGTCAATCCCACACCTTTCAGGGGTCCGAGGGGTCGTCCGTCTTCATACGTTTCGTACGTAGAGTTAATCAGTCGTTGGTCCACACCTTTCAGGGGTCCGAGGGGT
```

SV40 promoter

1,080 1,100 1,120 1,140 1,160

NsiI
SphI
NspI FauI
BstAPI FauI BccI BmrI

```
GCAGGCAGAAGTATGCAAAGCATGCATCTCAATTAGTCAGCAACCATAGTCCCGCCCCTAACTCCGCCCATCCCGCCCCTAACTCCGCCCAGTTCCGCCCATTCTCC
CGTCCGTCTTCATACGTTTCGTACGTAGAGTTAATCAGTCGTTGGTATCAGGGCGGGGATTGAGGCGGGTAGGGCGGGGATTGAGGCGGGTCAAGGCGGGTAAGAGG
```

SV40 promoter

1,180 1,200 1,220 1,240 1,260 1,280

BfaI
NmeAIII AvrII
BplI BplI BseRI

```
GCCCCATGGCTGACTAATTTTTTTTATTTATGCAGAGGCCGAGGCCGCCTCTGCCTCTGAGCTATTCNNNAAGTAGTGAGGAGGCTTTTTNGGAGNCCTAGGCTTTT
CGGGGTACCGACTGATTAAAAAAAATAAATACGTCTCCGGCTCCGGCGGAGACGGAGACTCGATAAGNNNTTCATCACTCCTCCGAAAAANCCTCNGGATCCGAAAA
```

SV40 promoter

1,300 1,320 1,340 1,360 1,380

Hpy188III

```
GCAAAAGCTCTTGACATNAATCATCGGC
CGTTTTCGAGAACTGTANTTAGTAGCCG
```

1,400 1,410

pCRE-LacZ (partial sequence) (3978 bp)

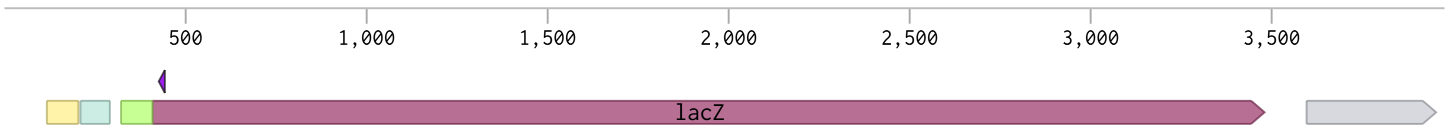

pCRE-LacZ (partial sequence) (3978 bp)

BsaWI
AgeI

```
TACGGTAGCATACCGGTACNNNNACCAGACANNNACGTCAGCTGCCAGATCCCATGGCCGTCATACTGTGACGTCTTTCAGACACCCCATTGACGTCAATGGGAGAA
ATGCCATCGTATGGCCATGNNNNTGGTCTGTNNNTGCAGTCGACGGTCTAGGGTACCGGCAGTATGACACTGCAGAAAGTCTGTGGGGTAACTGCAGTTACCCTCTT
```

20 40 60 80 100

BstBI
EcoRI
BsaAI ApoI

```
CCCGGTACGTGCACCAGACAGTGACGTCAGCTGCCAGATCCCATGGCCGTCATACTGTGACGTCTTTCAGACACCCCATTGACGTCAATGGGAGAACGAATTCGAAC
GGGCCATGCACGTGGTCTGTCACTGCAGTCGACGGTCTAGGGTACCGGCAGTATGACACTGCAGAAAGTCTGTGGGGTAACTGCAGTTACCCTCTTGCTTAAGCTTG
```

CRE

120 140 160 180 200

PaeR7I
PspXI AloI
XhoI BsaXI
PleI TliI KpnI
RsrII MlyI SmlI Acc65I

```
ACGCAGATGCAGTCGGGGCGGCGCGGTCCGAGGTCCACTTCGCATATTAAGGTGACGCGTGTGGCCTCGAACACCGACTCGAGCCGCTTAAAAGTTGGTACCGCCAC
TGCGTCTACGTCAGCCCCGCCGCGCCAGGCTCCAGGTGAAGCGTATAATTCCACTGCGCACACCGGAGCTTGTGGCTGAGCTCGGCGAATTTTCAACCATGGCGGTG
```

TK Minimal promoter

220 240 260 280 300 320

PasI

```
CATGAACTCCTTCTCCACAAGCGCCTTCGGTCCAGTTGCCTTCTCCCTGGGCCTGCTCCTGGTGTTGCCTGCTGCCTTCCCTGCCCCAACCATGATTACGGATTCAC
GTACTTGAGGAAGAGGTGTTCGCGGAAGCCAGGTCAACGGAAGAGGGACCCGGACGAGGACCACAACGGACGACGGAAGGGACGGGGTTGGTACTAATGCCTAAGTG
```

IL6-ss lacZ

340 360 380 400 420

EarI

```
TGGCCGTCGTTTTACAACGTCGTGACTGGGAAAACCCTGGCGTTACCCAACTTAATCGCCTTGCAGCACATCCCCCTTTCGCCAGCTGGCGTAATAGCGAAGAGGCC
ACCGGCAGCAAAATGTTGCAGCACTGACCCTTTTGGGACCGCAATGGGTTGAATTAGCGGAACGTCGTGTAGGGGGAAAGCGGTCGACCGCATTATCGCTTCTCCGG
```

M13 fwd
lacZ

440 460 480 500 520

FspI BglI

```
CGCACCGATCGCCCTTCCCAACAGTTGCGCAGCCTGAATGGCGAATGGCGCTTTGCCTGGTTTCCGGCACCAGAAGCGGTGCCGGAAAGCTGGCTGGAGTGCGATCT
GCGTGGCTAGCGGGAAGGGTTGTCAACGCGTCGGACTTACCGCTTACCGCGAAACGGACCAAAGGCCGTGGTCTTCGCCACGGCCTTTCGACCGACCTCACGCTAGA
```

lacZ

540 560 580 600 620 640

Bsu36I BaeI

TCCTGAGGCCGATACTGTCGTCGTCCCCTCAAACTGGCAGATGCACGGTTACGATGCGCCCATCTACACCAACGTAACCTATCCCATTACGGTCAATCCGCCGTTTG
AGGACTCCGGCTATGACAGCAGCAGGGGAGTTTGACCGTCTACGTGCCAATGCTACGCGGGTAGATGTGGTTGCATTGGATAGGGTAATGCCAGTTAGGCGGCAAAC

lacZ

660 680 700 720 740

MmeI SfcI NmeAIII HpaI

TTCCCACGGAGAATCCGACGGGTTGTTACTCGCTCACATTTAATGTTGATGAAAGCTGGCTACAGGAAGGCCAGACGCGAATTATTTTTGATGGCGTTAACTCGGCG
AAGGGTGCCTCTTAGGCTGCCCAACAATGAGCGAGTGTAAATTACAACTACTTTCGACCGATGTCCTTCCGGTCTGCGCTTAATAAAAACTACCGCAATTGAGCCGC

lacZ

760 780 800 820 840

ApoI Bpu10I MmeI

TTTCATCTGTGGTGCAACGGGCGCTGGGTCGGTTACGGCCAGGACAGTCGTTTGCCGTCTGAATTTGACCTGAGCGCATTTTTACGCGCCGGAGAAAACCGCCTCGC
AAAGTAGACACCACGTTGCCCGCGACCCAGCCAATGCCGGTCCTGTCAGCAAACGGCAGACTTAAACTGGACTCGCGTAAAAATGCGCGGCCTCTTTTGGCGGAGCG

lacZ

860 880 900 920 940 960

GGTGATGGTGCTGCGTTGGAGTGACGGCAGTTATCTGGAAGATCAGGATATGTGGCGGATGAGCGGCATTTTCCGTGACGTCTCGTTGCTGCATAAACCGACTACAC
CCACTACCACGACGCAACCTCACTGCCGTCAATAGACCTTCTAGTCCTATACACCGCCTACTCGCCGTAAAAGGCACTGCAGAGCAACGACGTATTTGGCTGATGTG

lacZ

980 1,000 1,020 1,040 1,060

TatI AcuI

AAATCAGCGATTTCCATGTTGCCACTCGCTTTAATGATGATTTCAGCCGCGCTGTACTGGAGGCTGAAGTTCAGATGTGCGGCGAGTTGCGTGACTACCTACGGGTA
TTTAGTCGCTAAAGGTACAACGGTGAGCGAAATTACTACTAAAGTCGGCGCGACATGACCTCCGACTTCAAGTCTACACGCCGCTCAACGCACTGATGGATGCCCAT

lacZ

1,080 1,100 1,120 1,140 1,160

BspMI BspDI
BfuAI ClaI

ACAGTTTCTTTATGGCAGGGTGAAACGCAGGTCGCCAGCGGCACCGCGCCTTTCGGCGGTGAAATTATCGATGAGCGTGGTGGTTATGCCGATCGCGTCACACTACG
TGTCAAAGAAATACCGTCCCACTTTGCGTCCAGCGGTCGCCGTGGCGCGGAAAGCCGCCACTTTAATAGCTACTCGCACCACCAATACGGCTAGCGCAGTGTGATGC

lacZ

1,180 1,200 1,220 1,240 1,260 1,280

TCTGAACGTCGAAAACCCGAAACTGTGGAGCGCCGAAATCCCGAATCTCTATCGTGCGGTGGTTGAACTGCACACCGCCGACGGCACGCTGATTGAAGCAGAAGCCT
AGACTTGCAGCTTTTGGGCTTTGACACCTCGCGGCTTTAGGGCTTAGAGATAGCACGCCACCAACTTGACGTGTGGCGGCTGCCGTGCGACTAACTTCGTCTTCGGA

lacZ

1,300 1,320 1,340 1,360 1,380

BssSαI
HpaI BssSI

GCGATGTCGGTTTCCGCGAGGTGCGGATTGAAAATGGTCTGCTGCTGCTGAACGGCAAGCCGTTGCTGATTCGAGGCGTTAACCGTCACGAGCATCATCCTCTGCAT
CGCTACAGCCAAAGGCGCTCCACGCCTAACTTTTACCAGACGACGACGACTTGCCGTTCGGCAACGACTAAGCTCCGCAATTGGCAGTGCTCGTAGTAGGAGACGTA

lacZ

1,400 1,420 1,440 1,460 1,480

EcoRV

GGTCAGGTCATGGATGAGCAGACGATGGTGCAGGATATCCTGCTGATGAAGCAGAACAACTTTAACGCCGTGCGCTGTTCGCATTATCCGAACCATCCGCTGTGGTA
CCAGTCCAGTACCTACTCGTCTGCTACCACGTCCTATAGGACGACTACTTCGTCTTGTTGAAATTGCGGCACGCGACAAGCGTAATAGGCTTGGTAGGCGACACCAT

lacZ

1,500 1,520 1,540 1,560 1,580 1,600

DraIII SspI PflMI

CACGCTGTGCGACCGCTACGGCCTGTATGTGGTGGATGAAGCCAATATTGAAACCCACGGCATGGTGCCAATGAATCGTCTGACCGATGATCCGCGCTGGCTACCGG
GTGCGACACGCTGGCGATGCCGGACATACACCACCTACTTCGGTTATAACTTTGGGTGCCGTACCACGGTTACTTAGCAGACTGGCTACTAGGCGCGACCGATGGCC

lacZ

1,620 1,640 1,660 1,680 1,700

BclI
BsaBI BcgI

CGATGAGCGAACGCGTAACGCGAATGGTGCAGCGCGATCGTAATCACCCGAGTGTGATCATCTGGTCGCTGGGGAATGAATCAGGCCACGGCGCTAATCACGACGCG
GCTACTCGCTTGCGCATTGCGCTTACCACGTCGCGCTAGCATTAGTGGGCTCACACTAGTAGACCAGCGACCCCTTACTTAGTCCGGTGCCGCGATTAGTGCTGCGC

lacZ

1,720 1,740 1,760 1,780 1,800

BssHII
MauBI

CTGTATCGCTGGATCAAATCTGTCGATCCTTCCCGCCCGGTGCAGTATGAAGGCGGCGGAGCCGACACCACGGCCACCGATATTATTTGCCCGATGTACGCGCGCGT
GACATAGCGACCTAGTTTAGACAGCTAGGAAGGGCGGGCCACGTCATACTTCCGCCGCCTCGGCTGTGGTGCCGGTGGCTATAATAAACGGGCTACATGCGCGCGCA

lacZ

1,820 1,840 1,860 1,880 1,900 1,920

BbsI PflMI

GGATGAAGACCAGCCCTTCCCGGCTGTGCCGAAATGGTCCATCAAAAAATGGCTTTCGCTACCTGGAGAGACGCGCCCGCTGATCCTTTGCGAATACGCCCACGCGA
CCTACTTCTGGTCGGGAAGGGCCGACACGGCTTTACCAGGTAGTTTTTTACCGAAAGCGATGGACCTCTCTGCGCGGGCGACTAGGAAACGCTTATGCGGGTGCGCT

lacZ

1,940 1,960 1,980 2,000 2,020

BciVI
BcgI

TGGGTAACAGTCTTGGCGGTTTCGCTAAATACTGGCAGGCGTTTCGTCAGTATCCCCGTTTACAGGGCGGCTTCGTCTGGGACTGGGTGGATCAGTCGCTGATTAAA
ACCCATTGTCAGAACCGCCAAAGCGATTTATGACCGTCCGCAAAGCAGTCATAGGGGCAAATGTCCCGCCGAAGCAGACCCTGACCCACCTAGTCAGCGACTAATTT

lacZ

2,040 2,060 2,080 2,100 2,120 2,140

TATGATGAAAACGGCAACCCGTGGTCGGCTTACGGCGGTGATTTTGGCGATACGCCGAACGATCGCCAGTTCTGTATGAACGGTCTGGTCTTTGCCGACCGCACGCC
ATACTACTTTTGCCGTTGGGCACCAGCCGAATGCCGCCACTAAAACCGCTATGCGGCTTGCTAGCGGTCAAGACATACTTGCCAGACCAGAAACGGCTGGCGTGCGG

lacZ

2,160 2,180 2,200 2,220 2,240

AfeI

GCATCCAGCGCTGACGGAAGCAAAACACCAGCAGCAGTTTTTCCAGTTCCGTTTATCCGGGCAAACCATCGAAGTGACCAGCGAATACCTGTTCCGTCATAGCGATA
CGTAGGTCGCGACTGCCTTCGTTTTGTGGTCGTCGTCAAAAAGGTCAAGGCAAATAGGCCCGTTTGGTAGCTTCACTGGTCGCTTATGGACAAGGCAGTATCGCTAT

lacZ

2,260 2,280 2,300 2,320 2,340

BanII
SacI
Eco53kI

ACGAGCTCCTGCACTGGATGGTGGCGCTGGATGGTAAGCCGCTGGCAAGCGGTGAAGTGCCTCTGGATGTCGCTCCACAAGGTAAACAGTTGATTGAACTGCCTGAA
TGCTCGAGGACGTGACCTACCACCGCGACCTACCATTCGGCGACCGTTCGCCACTTCACGGAGACCTACAGCGAGGTGTTCCATTTGTCAACTAACTTGACGGACTT

lacZ

2,360 2,380 2,400 2,420 2,440 2,460

CTACCGCAGCCGGAGAGCGCCGGGCAACTCTGGCTCACAGTACGCGTAGTGCAACCGAACGCGACCGCATGGTCAGAAGCCGGGCACATCAGCGCCTGGCAGCAGTG
GATGGCGTCGGCCTCTCGCGGCCCGTTGAGACCGAGTGTCATGCGCATCACGTTGGCTTGCGCTGGCGTACCAGTCTTCGGCCCGTGTAGTCGCGGACCGTCGTCAC

lacZ

2,480 2,500 2,520 2,540 2,560

BtsαI
BtsI
BstXI
XcmI

GCGTCTGGCGGAAAACCTCAGTGTGACGCTCCCCGCCGCGTCCCACGCCATCCCGCATCTGACCACCAGCGAAATGGATTTTTGCATCGAGCTGGGTAATAAGCGTT
CGCAGACCGCCTTTTGGAGTCACACTGCGAGGGGCGGCGCAGGGTGCGGTAGGGCGTAGACTGGTGGTCGCTTTACCTAAAAACGTAGCTCGACCCATTATTCGCAA

lacZ

2,580 2,600 2,620 2,640 2,660

BglI

GGCAATTTAACCGCCAGTCAGGCTTTCTTTCACAGATGTGGATTGGCGATAAAAAACAACTGCTGACGCCGCTGCGCGATCAGTTCACCCGTGCACCGCTGGATAAC
CCGTTAAATTGGCGGTCAGTCCGAAAGAAAGTGTCTACACCTAACCGCTATTTTTTGTTGACGACTGCGGCGACGCGCTAGTCAAGTGGGCACGTGGCGACCTATTG

lacZ

2,680 2,700 2,720 2,740 2,760 2,780

BtsI
BtsαI
GACATTGGCGTAAGTGAAGCGACCCGCATTGACCCTAACGCCTGGGTCGAACGCTGGAAGGCGGCGGGCCATTACCAGGCCGAAGCAGCGTTGTTGCAGTGCACGGC
CTGTAACCGCATTCACTTCGCTGGGCGTAACTGGGATTGCGGACCCAGCTTGCGACCTTCCGCCGCCCGGTAATGGTCCGGCTTCGTCGCAACAACGTCACGTGCCG
lacZ
2,800 2,820 2,840 2,860 2,880

BsaWI
AGATACACTTGCTGATGCGGTGCTGATTACGACCGCTCACGCGTGGCAGCATCAGGGGAAAACCTTATTTATCAGCCGGAAAACCTACCGGATTGATGGTAGTGGTC
TCTATGTGAACGACTACGCCACGACTAATGCTGGCGAGTGCGCACCGTCGTAGTCCCCTTTTGGAATAAATAGTCGGCCTTTTGGATGGCCTAACTACCATCACCAG
lacZ
2,900 2,920 2,940 2,960 2,980

BspMI
BfuAI
AAATGGCGATTACCGTTGATGTTGAAGTGGCGAGCGATACACCGCATCCGGCGCGGATTGGCCTGAACTGCCAGCTGGCGCAGGTAGCAGAGCGGGTAAACTGGCTC
TTTACCGCTAATGGCAACTACAACTTCACCGCTCGCTATGTGGCGTAGGCCGCGCCTAACCGGACTTGACGGTCGACCGCGTCCATCGTCTCGCCCATTTGACCGAG
lacZ
3,000 3,020 3,040 3,060 3,080 3,100

BbsI
AccI BsiWI
PciI BstZ17I
GGATTAGGGCCGCAAGAAAACTATCCCGACCGCCTTACTGCCGCCTGTTTTGACCGCTGGGATCTGCCATTGTCAGACATGTATACCCCGTACGTCTTCCCGAGCGA
CCTAATCCCGGCGTTCTTTTGATAGGGCTGGCGGAATGACGGCGGACAAAACTGGCGACCCTAGACGGTAACAGTCTGTACATATGGGGCATGCAGAAGGGCTCGCT
lacZ
3,120 3,140 3,160 3,180 3,200

BstXI SfcI
AAACGGTCTGCGCTGCGGGACGCGCGAATTGAATTATGGCCCACACCAGTGGCGCGGCGACTTCCAGTTCAACATCAGCCGCTACAGTCAACAGCAACTGATGGAAA
TTTGCCAGACGCGACGCCCTGCGCGCTTAACTTAATACCGGGTGTGGTCACCGCGCCGCTGAAGGTCAAGTTGTAGTCGGCGATGTCAGTTGTCGTTGACTACCTTT
lacZ
3,220 3,240 3,260 3,280 3,300

XcmI PleI
NdeI MlyI PfoI BanII
CCAGCCATCGCCATCTGCTGCACGCGGAAGAAGGCACATGGCTGAATATCGACGGTTTCCATATGGGGATTGGTGGCGACGACTCCTGGAGCCCGTCAGTATCGGCG
GGTCGGTAGCGGTAGACGACGTGCGCCTTCTTCCGTGTACCGACTTATAGCTGCCAAAGGTATACCCCTAACCACCGCTGCTGAGGACCTCGGGCAGTCATAGCCGC
lacZ
3,320 3,340 3,360 3,380 3,400 3,420

FspAI BfaI
BlpI FspI BsiWI

GAGTTCCAGCTGAGCGCCGGTCGCTACCATTACCAGTTGGTCTGGTGTCAAAAATAATGCGCATGAGACGTACGCTAGAAATAATTCTTACTGTCATGCCAAGTAAG
CTCAAGGTCGACTCGCGGCCAGCGATGGTAATGGTCAACCAGACCACAGTTTTTATTACGCGTACTCTGCATGCGATCTTTATTAAGAATGACAGTACGGTTCATTC

lacZ

3,440 3,460 3,480 3,500 3,520

ATGCTTTTCTGGCGCAGCACCATGGCCTGAAATAACCTCTGAAAGAGGAACTTGGTTAGCTACCTTCTGAGGCGGAAAGAACCAGCTGTGGAATGTGTGTCAGTTAG
TACGAAAAGACCGCGTCGTGGTACCGGACTTTATTGGAGACTTTCTCCTTGAACCAATCGATGGAAGACTCCGCCTTTCTTGGTCGACACCTTACACACAGTCAATC

SV40 promoter

3,540 3,560 3,580 3,600 3,620

NsiI
SphI
BstAPI SexAI

GGTGTGGAAAGTCCCCAGGCTCCCCAGCAGGCAGAAGTATGCAAAGCATGCATCTCAATTAGTCAGCAACCAGGTGTGGAAAGTCCCCAGGCTCCCCAGCAGGCAGA
CCACACCTTTCAGGGGTCCGAGGGGTCGTCCGTCTTCATACGTTTCGTACGTAGAGTTAATCAGTCGTTGGTCCACACCTTTCAGGGGTCCGAGGGGTCGTCCGTCT

SV40 promoter

3,640 3,660 3,680 3,700 3,720 3,740

NsiI
SphI
BstAPI

AGTATGCAAAGCATGCATCTCAATTAGTCAGCAACCATAGTCCCGCCCCTAACTCCGCCCATCCCGCCCCTAACTCCGCCCAGTTCCGCCCATTCTCCGCCCCATGG
TCATACGTTTCGTACGTAGAGTTAATCAGTCGTTGGTATCAGGGCGGGGATTGAGGCGGGTAGGGCGGGGATTGAGGCGGGTCAAGGCGGGTAAGAGGCGGGGTACC

SV40 promoter

3,760 3,780 3,800 3,820 3,840

BfaI
NmeAIII AvrII
BplI BplI BseRI

CTGACTAATTTTTTTTATTTATGCAGAGGCCGAGGCCGCCTCTGCCTCTGAGCTATTCNNNAAGTAGTGAGGAGGCTTTTTNGGAGNCCTAGGCTTTTGCAAAAGCT
GACTGATTAAAAAAAATAAATACGTCTCCGGCTCCGGCGGAGACGGAGACTCGATAAGNNNTTCATCACTCCTCCGAAAAANCCTCNGGATCCGAAAACGTTTTCGA

SV40 promoter

3,860 3,880 3,900 3,920 3,940

CTTGACATNAATCATCGGC
GAACTGTANTTAGTAGCCG

3,960 3,970

pULS-Ex4 (7954 bp)

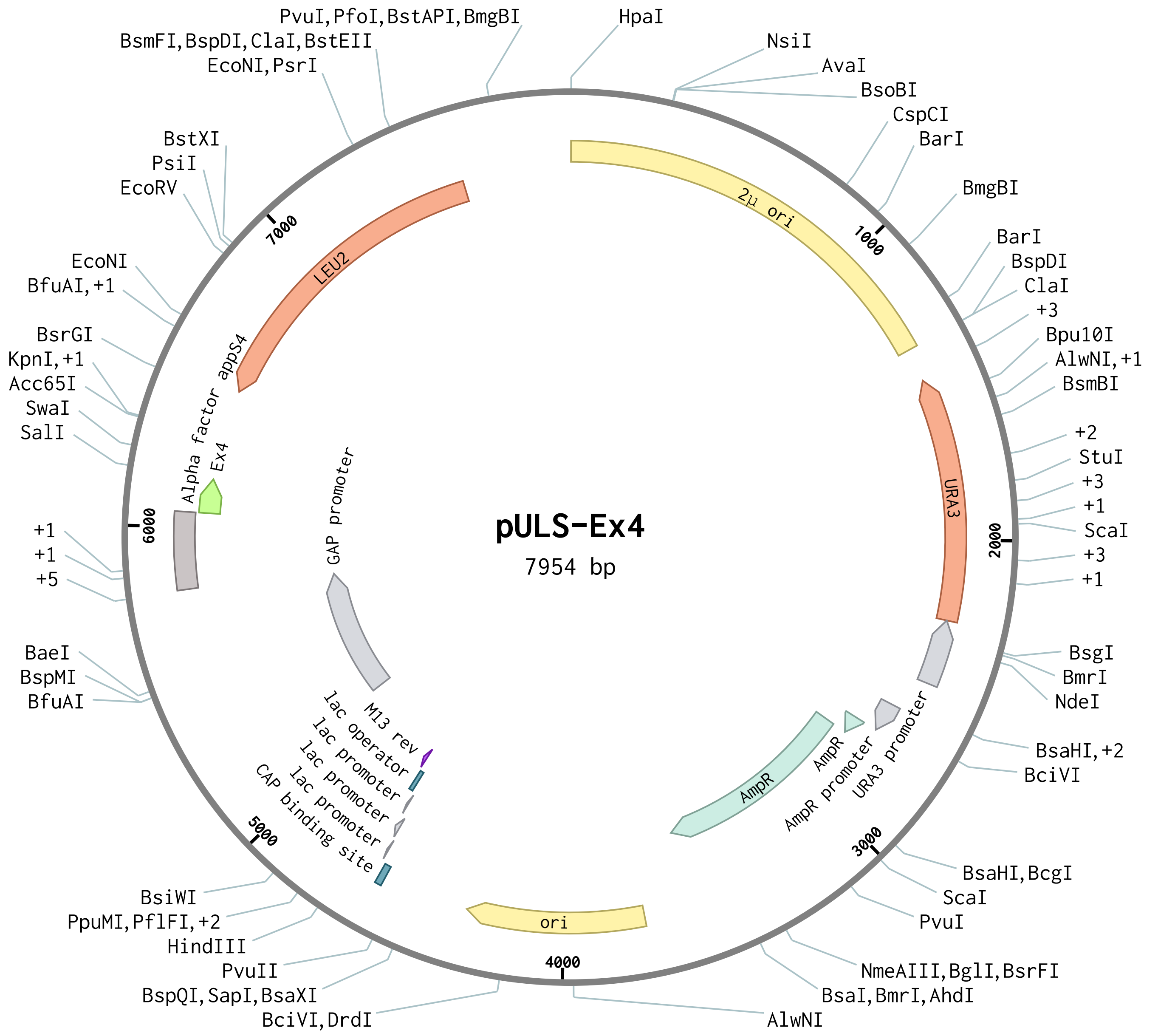

pULS-Ex4 (7954 bp)

```
HpaI
GTTAACGAAGCATCTGTGCTTCATTTTGTAGAACAAAAATGCAACGCGAGAGCGCTAATTTTTCAAACAAAGAATCTGAGCTG
CAATTGCTTCGTAGACACGAAGTAAAACATCTTGTTTTTACGTTGCGCTCTCGCGATTAAAAAGTTTGTTTCTTAGACTCGAC
2µ ori
10 20 30 40 50 60 70 80

CATTTTTACAGAACAGAAATGCAACGCGAAAGCGCTATTTTACCAACGAAGAATCTGTGCTTCATTTTTGTAAAACAAAAATG
GTAAAAATGTCTTGTCTTTACGTTGCGCTTTCGCGATAAAATGGTTGCTTCTTAGACACGAAGTAAAAACATTTTGTTTTTAC
2µ ori
90 100 110 120 130 140 150 160

CAACGCGAGAGCGCTAATTTTTCAAACAAAGAATCTGAGCTGCATTTTTACAGAACAGAAATGCAACGCGAGAGCGCTATTTT
GTTGCGCTCTCGCGATTAAAAAGTTTGTTTCTTAGACTCGACGTAAAAATGTCTTGTCTTTACGTTGCGCTCTCGCGATAAAA
2µ ori
170 180 190 200 210 220 230 240

BsoBI
AvaI
NsiI
ACCAACAAAGAATCTATACTTCTTTTTTGTTCTACAAAAATGCATCCCGAGAGCGCTATTTTTCTAACAAAGCATCTTAGATT
TGGTTGTTTCTTAGATATGAAGAAAAAACAAGATGTTTTTACGTAGGGCTCTCGCGATAAAAAGATTGTTTCGTAGAATCTAA
2µ ori
250 260 270 280 290 300 310 320 330

ACTTTTTTTCTCCTTTGTGCGCTCTATAATGCAGTCTCTTGATAACTTTTTGCACTGTAGGTCCGTTAAGGTTAGAAGAAGGC
TGAAAAAAAGAGGAAACACGCGAGATATTACGTCAGAGAACTATTGAAAAACGTGACATCCAGGCAATTCCAATCTTCTTCCG
2µ ori
340 350 360 370 380 390 400 410

TACTTTGGTGTCTATTTTCTCTTCCATAAAAAAAGCCTGACTCCACTTCCCGCGTTTACTGATTACTAGCGAAGCTGCGGGTG
ATGAAACCACAGATAAAAGAGAAGGTATTTTTTTCGGACTGAGGTGAAGGGCGCAAATGACTAATGATCGCTTCGACGCCCAC
2µ ori
420 430 440 450 460 470 480 490

CATTTTTTCAAGATAAAGGCATCCCCGATTATATTCTATACCGATGTGGATTGCGCATACTTTGTGAACAGAAAGTGATAGCG
GTAAAAAAGTTCTATTTCCGTAGGGGCTAATATAAGATATGGCTACACCTAACGCGTATGAAACACTTGTCTTTCACTATCGC
2µ ori
500 510 520 530 540 550 560 570 580
```

TTGATGATTCTTCATTGGTCAGAAAATTATGAACGGTTTCTTCTATTTTGTCTCTATATACTACGTATAGGAAATGTTTACAT
AACTACTAAGAAGTAACCAGTCTTTTAATACTTGCCAAAGAAGATAAAACAGAGATATATGATGCATATCCTTTACAAATGTA
2μ ori
590 600 610 620 630 640 650 660

TTTCGTATTGTTTTCGATTCACTCTATGAATAGTTCTTACTACAATTTTTTTGTCTAAAGAGTAATACTAGAGATAAACATAA
AAAGCATAACAAAAGCTAAGTGAGATACTTATCAAGAATGATGTTAAAAAAACAGATTTCTCATTATGATCTCTATTTGTATT
2μ ori
670 680 690 700 710 720 730 740

AAAATGTAGAGGTCGAGTTTAGATGCAAGTTCAAGGAGCGAAAGGTGGATGGGTAGGTTATATAGGGATATAGCACAGAGATA
TTTTACATCTCCAGCTCAAATCTACGTTCAAGTTCCTCGCTTTCCACCTACCCATCCAATATATCCCTATATCGTGTCTCTAT
2μ ori
750 760 770 780 790 800 810 820 830

CspCI
TATAGCAAAGAGATACTTTTGAGCAATGTTTGTGGAAGCGGTATTCGCAATATTTTAGTAGCTCGTTACAGTCCGGTGCGTTT
ATATCGTTTCTCTATGAAAACTCGTTACAAACACCTTCGCCATAAGCGTTATAAAATCATCGAGCAATGTCAGGCCACGCAAA
2μ ori
840 850 860 870 880 890 900 910

BarI
TTGGTTTTTTGAAAGTGCGTCTTCAGAGCGCTTTTGGTTTTCAAAAGCGCTCTGAAGTTCCTATACTTTCTAGCTAGAGAATA
AACCAAAAAACTTTCACGCAGAAGTCTCGCGAAAACCAAAAGTTTTCGCGAGACTTCAAGGATATGAAAGATCGATCTCTTAT
2μ ori
920 930 940 950 960 970 980 990

GGAACTTCGGAATAGGAACTTCAAAGCGTTTCCGAAAACGAGCGCTTCCGAAAATGCAACGCGAGCTGCGCACATACAGCTCA
CCTTGAAGCCTTATCCTTGAAGTTTCGCAAAGGCTTTTGCTCGCGAAGGCTTTTACGTTGCGCTCGACGCGTGTATGTCGAGT
2μ ori
1,000 1,010 1,020 1,030 1,040 1,050 1,060 1,070

BmgBI
CTGTTCACGTCGCACCTATATCTGCGTGTTGCCTGTATATATATATACATGAGAAGAACGGCATAGTGCGTGTTTATGCTTAA
GACAAGTGCAGCGTGGATATAGACGCACAACGGACATATATATATATGTACTCTTCTTGCCGTATCACGCACAAATACGAATT
2μ ori
1,080 1,090 1,100 1,110 1,120 1,130 1,140 1,150 1,160

```
ATGCGTACTTATATGCGTCTATTTATGTAGGATGAAAGGTAGTCTAGTACCTCCTGTGATATTATCCCATTCCATGCGGGGTA
TACGCATGAATATACGCAGATAAATACATCCTACTTTCCATCAGATCATGGAGGACACTATAATAGGGTAAGGTACGCCCCAT
2µ ori
1,170 1,180 1,190 1,200 1,210 1,220 1,230 1,240

BarI
TCGTATGCTTCCTTCAGCACTACCCTTTAGCTGTTCTATATGCTGCCACTCCTCAATTGGATTAGTCTCATCCTTCAATGCTA
AGCATACGAAGGAAGTCGTGATGGGAAATCGACAAGATATACGACGGTGAGGAGTTAACCTAATCAGAGTAGGAAGTTACGAT
2µ ori
1,250 1,260 1,270 1,280 1,290 1,300 1,310 1,320

BspDI
ClaI
TCATTTCCTTTGATATTGGATCGATCCGATGATAAGCTGTCAAACATGAGAATTGGGTAATAACTGATATAATTAAATTGAAG
AGTAAAGGAAACTATAACCTAGCTAGGCTACTATTCGACAGTTTGTACTCTTAACCCATTATTGACTATATTAATTTAACTTC
2µ ori
1,330 1,340 1,350 1,360 1,370 1,380 1,390 1,400 1,410

BstZ17I NsiI PsiI
CTCTAATTTGTGAGTTTAGTATACATGCATTTACTTATAATACAGTTTTTTAGTTTTGCTGGCCGCATCTTCTCAAATATGCT
GAGATTAAACACTCAAATCATATGTACGTAAATGAATATTATGTCAAAAAATCAAAACGACCGGCGTAGAAGAGTTTATACGA
URA3
1,420 1,430 1,440 1,450 1,460 1,470 1,480 1,490

Bpu10I
TCCCAGCCTGCTTTTCTGTAACGTTCACCCTCTACCTTAGCATCCCTTCCCTTTGCAAATAGTCCTCTTCCAACAATAATAAT
AGGGTCGGACGAAAAGACATTGCAAGTGGGAGATGGAATCGTAGGGAAGGGAAACGTTTATCAGGAGAAGGTTGTTATTATTA
URA3
1,500 1,510 1,520 1,530 1,540 1,550 1,560 1,570

AlwNI
BsaI BsmBI
GTCAGATCCTGTAGAGACCACATCATCCACGGTTCTATACTGTTGACCCAATGCGTCTCCCTTGTCATCTAAACCCACACCGG
CAGTCTAGGACATCTCTGGTGTAGTAGGTGCCAAGATATGACAACTGGGTTACGCAGAGGGAACAGTAGATTTGGGTGTGGCC
URA3
1,580 1,590 1,600 1,610 1,620 1,630 1,640 1,650 1,660

GTGTCATAATCAACCAATCGTAACCTTCATCTCTTCCACCCATGTCTCTTTGAGCAATAAAGCCGATAACAAAATCTTTGTCG
CACAGTATTAGTTGGTTAGCATTGGAAGTAGAGAAGGTGGGTACAGAGAAACTCGTTATTTCGGCTATTGTTTTAGAAACAGC
URA3
1,670 1,680 1,690 1,700 1,710 1,720 1,730 1,740
```

BspQI
SapI

CTCTTCGCAATGTCAACAGTACCCTTAGTATATTCTCCAGTAGATAGGGAGCCCTTGCATGACAATTCTGCTAACATCAAAAG
GAGAAGCGTTACAGTTGTCATGGGAATCATATAAGAGGTCATCTATCCCTCGGGAACGTACTGTTAAGACGATTGTAGTTTTC

URA3

1,750 1,760 1,770 1,780 1,790 1,800 1,810 1,820

ApaI
StuI PspOMI BsmI

GCCTCTAGGTTCCTTTGTTACTTCTTCTGCCGCCTGCTTCAAACCGCTAACAATACCTGGGCCCACCACACCGTGTGCATTCG
CGGAGATCCAAGGAAACAATGAAGAAGACGGCGGACGAAGTTTGGCGATTGTTATGGACCCGGGTGGTGTGGCACACGTAAGC

URA3

1,830 1,840 1,850 1,860 1,870 1,880 1,890 1,900

BstZ17I ScaI

TAATGTCTGCCCATTCTGCTATTCTGTATACACCCGCAGAGTACTGCAATTTGACTGTATTACCAATGTCAGCAAATTTTCTG
ATTACAGACGGGTAAGACGATAAGACATATGTGGGCGTCTCATGACGTTAAACTGACATAATGGTTACAGTCGTTTAAAAGAC

URA3

1,910 1,920 1,930 1,940 1,950 1,960 1,970 1,980 1,990

NcoI

TCTTCGAAGAGTAAAAAATTGTACTTGGCGGATAATGCCTTTAGCGGCTTAACTGTGCCCTCCATGGAAAAATCAGTCAAGAT
AGAAGCTTCTCATTTTTTAACATGAACCGCCTATTACGGAAATCGCCGAATTGACACGGGAGGTACCTTTTTAGTCAGTTCTA

URA3

2,000 2,010 2,020 2,030 2,040 2,050 2,060 2,070

EcoRV PpuMI BsmFI

ATCCACATGTGTTTTTAGTAAACAAATTTTGGGACCTAATGCTTCAACTAACTCCAGTAATTCCTTGGTGGTACGAACATCCA
TAGGTGTACACAAAAATCATTTGTTTAAAACCCTGGATTACGAAGTTGATTGAGGTCATTAAGGAACCACCATGCTTGTAGGT

URA3

2,080 2,090 2,100 2,110 2,120 2,130 2,140 2,150

ATGAAGCACACAAGTTTGTTTGCTTTTCGTGCATGATATTAAATAGCTTGGCAGCAACAGGACTAGGATGAGTAGCAGCACGT
TACTTCGTGTGTTCAAACAAACGAAAAGCACGTACTATAATTTATCGAACCGTCGTTGTCCTGATCCTACTCATCGTCGTGCA

URA3

2,160 2,170 2,180 2,190 2,200 2,210 2,220 2,230 2,240

BsgI

TCCTTATATGTAGCTTTCGACATGATTTATCTTCGTTTCCTGCATGTTTTTGTTCTGTGCAGTTGGGTTAAGAATACTGGGCA
AGGAATATACATCGAAAGCTGTACTAAATAGAAGCAAAGGACGTACAAAAACAAGACACGTCAACCCAATTCTTATGACCCGT

URA3 | URA3 promoter

2,250 2,260 2,270 2,280 2,290 2,300 2,310 2,320

BmrI NdeI

ATTTCATGTTTCTTCAACACTACATATGCGTATATATACCAATCTAAGTCTGTGCTCCTTCCTTCGTTCTTCCTTCTGTTCGG
TAAAGTACAAAGAAGTTGTGATGTATACGCATATATATGGTTAGATTCAGACACGAGGAAGGAAGCAAGAAGGAAGACAAGCC

URA3 promoter

2,330 2,340 2,350 2,360 2,370 2,380 2,390 2,400

AGATTACCGAATCAAAAAAATTTCAAAGAAACCGAAATCAAAAAAAAGAATAAAAAAAAAATGATGAATTGAATTGAAAAGCT
TCTAATGGCTTAGTTTTTTTAAAGTTTCTTTGGCTTTAGTTTTTTTTCTTATTTTTTTTTTACTACTTAACTTAACTTTTCGA

URA3 promoter

2,410 2,420 2,430 2,440 2,450 2,460 2,470 2,480 2,490

AatII
ZraI
BsaHI

AATTCTTGAAGACGAAAGGGCCTCGTGATACGCCTATTTTTATAGGTTAATGTCATGATAATAATGGTTTCTTAGACGTCAGG
TTAAGAACTTCTGCTTTCCCGGAGCACTATGCGGATAAAAATATCCAATTACAGTACTATTATTACCAAAGAATCTGCAGTCC

2,500 2,510 2,520 2,530 2,540 2,550 2,560 2,570

BciVI

TGGCACTTTTCGGGGAAATGTGCGCGGAACCCCTATTTGTTTATTTTTCTAAATACATTCAAATATGTATCCGCTCATGAGAC
ACCGTGAAAAGCCCCTTTACACGCGCCTTGGGGATAAACAAATAAAAAGATTTATGTAAGTTTATACATAGGCGAGTACTCTG

AmpR promoter

2,580 2,590 2,600 2,610 2,620 2,630 2,640 2,650

AATAACCCTGATAAATGCTTCAATAATATTGAAAAAGGAAGAGTATGAGTATTCAACATTTCCGTGTCGCCCTTATTCCCTTT
TTATTGGGACTATTTACGAAGTTATTATAACTTTTTCCTTCTCATACTCATAAGTTGTAAAGGCACAGCGGGAATAAGGGAAA

AmpR promoter | AmpR

2,660 2,670 2,680 2,690 2,700 2,710 2,720 2,730

TTTGCGGCATTTTGCCTTCCTGTTTTTGCTCACCCAGAAACGCTGGTGAAAGTAAAAGATGCTGAAGATCAGTTGGGTGCACG
AAACGCCGTAAAACGGAAGGACAAAAACGAGTGGGTCTTTGCGACCACTTTCATTTTCTACGACTTCTAGTCAACCCACGTGC

AmpR | AmpR

2,740 2,750 2,760 2,770 2,780 2,790 2,800 2,810 2,820

AGTGGGTTACATCGAACTGGATCTCAACAGCGGTAAGATCCTTGAGAGTTTTCGCCCCGAAGAACGTTTTCCAATGATGAGCA
TCACCCAATGTAGCTTGACCTAGAGTTGTCGCCATTCTAGGAACTCTCAAAAGCGGGGCTTCTTGCAAAAGGTTACTACTCGT
AmpR
2,830 2,840 2,850 2,860 2,870 2,880 2,890 2,900

BsaHI BcgI
CTTTTAAAGTTCTGCTATGTGGCGCGGTATTATCCCGTATTGACGCCGGGCAAGAGCAACTCGGTCGCCGCATACACTATTCT
GAAAATTTCAAGACGATACACCGCGCCATAATAGGGCATAACTGCGGCCCGTTCTCGTTGAGCCAGCGGCGTATGTGATAAGA
AmpR
2,910 2,920 2,930 2,940 2,950 2,960 2,970 2,980

ScaI
CAGAATGACTTGGTTGAGTACTCACCAGTCACAGAAAAGCATCTTACGGATGGCATGACAGTAAGAGAATTATGCAGTGCTGC
GTCTTACTGAACCAACTCATGAGTGGTCAGTGTCTTTTCGTAGAATGCCTACCGTACTGTCATTCTCTTAATACGTCACGACG
AmpR
2,990 3,000 3,010 3,020 3,030 3,040 3,050 3,060 3,070

PvuI
CATAACCATGAGTGATAACACTGCGGCCAACTTACTTCTGACAACGATCGGAGGACCGAAGGAGCTAACCGCTTTTTTGCACA
GTATTGGTACTCACTATTGTGACGCCGGTTGAATGAAGACTGTTGCTAGCCTCCTGGCTTCCTCGATTGGCGAAAAAACGTGT
AmpR
3,080 3,090 3,100 3,110 3,120 3,130 3,140 3,150

ACATGGGGGATCATGTAACTCGCCTTGATCGTTGGGAACCGGAGCTGAATGAAGCCATACCAAACGACGAGCGTGACACCACG
TGTACCCCCTAGTACATTGAGCGGAACTAGCAACCCTTGGCCTCGACTTACTTCGGTATGGTTTGCTGCTCGCACTGTGGTGC
AmpR
3,160 3,170 3,180 3,190 3,200 3,210 3,220 3,230

ATGCCTGTAGCAATGGCAACAACGTTGCGCAAACTATTAACTGGCGAACTACTTACTCTAGCTTCCCGGCAACAATTAATAGA
TACGGACATCGTTACCGTTGTTGCAACGCGTTTGATAATTGACCGCTTGATGAATGAGATCGAAGGGCCGTTGTTAATTATCT
AmpR
3,240 3,250 3,260 3,270 3,280 3,290 3,300 3,310 3,320

NmeAIII BglI
CTGGATGGAGGCGGATAAAGTTGCAGGACCACTTCTGCGCTCGGCCCTTCCGGCTGGCTGGTTTATTGCTGATAAATCTGGAG
GACCTACCTCCGCCTATTTCAACGTCCTGGTGAAGACGCGAGCCGGGAAGGCCGACCGACCAAATAACGACTATTTAGACCTC
AmpR
3,330 3,340 3,350 3,360 3,370 3,380 3,390 3,400

BsrFI BsaI BmrI

CCGGTGAGCGTGGGTCTCGCGGTATCATTGCAGCACTGGGGCCAGATGGTAAGCCCTCCCGTATCGTAGTTATCTACACGACG
GGCCACTCGCACCCAGAGCGCCATAGTAACGTCGTGACCCCGGTCTACCATTCGGGAGGGCATAGCATCAATAGATGTGCTGC

AmpR

3,410 3,420 3,430 3,440 3,450 3,460 3,470 3,480

AhdI

GGGAGTCAGGCAACTATGGATGAACGAAATAGACAGATCGCTGAGATAGGTGCCTCACTGATTAAGCATTGGTAACTGTCAGA
CCCTCAGTCCGTTGATACCTACTTGCTTTATCTGTCTAGCGACTCTATCCACGGAGTGACTAATTCGTAACCATTGACAGTCT

AmpR

3,490 3,500 3,510 3,520 3,530 3,540 3,550 3,560

CCAAGTTTACTCATATATACTTTAGATTGATTTAAAACTTCATTTTTAATTTAAAAGGATCTAGGTGAAGATCCTTTTTGATA
GGTTCAAATGAGTATATATGAAATCTAACTAAATTTTGAAGTAAAAATTAAATTTTCCTAGATCCACTTCTAGGAAAAACTAT

3,570 3,580 3,590 3,600 3,610 3,620 3,630 3,640 3,650

ATCTCATGACCAAAATCCCTTAACGTGAGTTTTCGTTCCACTGAGCGTCAGACCCCGTAGAAAAGATCAAAGGATCTTCTTGA
TAGAGTACTGGTTTTAGGGAATTGCACTCAAAAGCAAGGTGACTCGCAGTCTGGGGCATCTTTTCTAGTTTCCTAGAAGAACT

3,660 3,670 3,680 3,690 3,700 3,710 3,720 3,730

GATCCTTTTTTTCTGCGCGTAATCTGCTGCTTGCAAACAAAAAAACCACCGCTACCAGCGGTGGTTTGTTTGCCGGATCAAGA
CTAGGAAAAAAAGACGCGCATTAGACGACGAACGTTTGTTTTTTTGGTGGCGATGGTCGCCACCAAACAAACGGCCTAGTTCT

ori

3,740 3,750 3,760 3,770 3,780 3,790 3,800 3,810

GCTACCAACTCTTTTTCCGAAGGTAACTGGCTTCAGCAGAGCGCAGATACCAAATACTGTCCTTCTAGTGTAGCCGTAGTTAG
CGATGGTTGAGAAAAAGGCTTCCATTGACCGAAGTCGTCTCGCGTCTATGGTTTATGACAGGAAGATCACATCGGCATCAATC

ori

3,820 3,830 3,840 3,850 3,860 3,870 3,880 3,890 3,900

AlwNI

GCCACCACTTCAAGAACTCTGTAGCACCGCCTACATACCTCGCTCTGCTAATCCTGTTACCAGTGGCTGCTGCCAGTGGCGAT
CGGTGGTGAAGTTCTTGAGACATCGTGGCGGATGTATGGAGCGAGACGATTAGGACAATGGTCACCGACGACGGTCACCGCTA

ori

3,910 3,920 3,930 3,940 3,950 3,960 3,970 3,980

AAGTCGTGTCTTACCGGGTTGGACTCAAGACGATAGTTACCGGATAAGGCGCAGCGGTCGGGCTGAACGGGGGGTTCGTGCAC
TTCAGCACAGAATGGCCCAACCTGAGTTCTGCTATCAATGGCCTATTCCGCGTCGCCAGCCCGACTTGCCCCCCAAGCACGTG

ori

3,990 4,000 4,010 4,020 4,030 4,040 4,050 4,060

ACAGCCCAGCTTGGAGCGAACGACCTACACCGAACTGAGATACCTACAGCGTGAGCTATGAGAAAGCGCCACGCTTCCCGAAG
TGTCGGGTCGAACCTCGCTTGCTGGATGTGGCTTGACTCTATGGATGTCGCACTCGATACTCTTTCGCGGTGCGAAGGGCTTC
ori
4,070 4,080 4,090 4,100 4,110 4,120 4,130 4,140 4,150

BciVI
GGAGAAAGGCGGACAGGTATCCGGTAAGCGGCAGGGTCGGAACAGGAGAGCGCACGAGGGAGCTTCCAGGGGGAAACGCCTGG
CCTCTTTCCGCCTGTCCATAGGCCATTCGCCGTCCCAGCCTTGTCCTCTCGCGTGCTCCCTCGAAGGTCCCCCTTTGCGGACC
ori
4,160 4,170 4,180 4,190 4,200 4,210 4,220 4,230

DrdI
TATCTTTATAGTCCTGTCGGGTTTCGCCACCTCTGACTTGAGCGTCGATTTTTGTGATGCTCGTCAGGGGGGCGGAGCCTATG
ATAGAAATATCAGGACAGCCCAAAGCGGTGGAGACTGAACTCGCAGCTAAAAACACTACGAGCAGTCCCCCCGCCTCGGATAC
ori
4,240 4,250 4,260 4,270 4,280 4,290 4,300 4,310

GAAAAACGCCAGCAACGCGGCCTTTTTACGGTTCCTGGCCTTTTGCTGGCCTTTTGCTCACATGTTCTTTCCTGCGTTATCCC
CTTTTTGCGGTCGTTGCGCCGGAAAAATGCCAAGGACCGGAAAACGACCGGAAAACGAGTGTACAAGAAAGGACGCAATAGGG
4,320 4,330 4,340 4,350 4,360 4,370 4,380 4,390

CTGATTCTGTGGATAACCGTATTACCGCCTTTGAGTGAGCTGATACCGCTCGCCGCAGCCGAACGACCGAGCGCAGCGAGTCA
GACTAAGACACCTATTGGCATAATGGCGGAAACTCACTCGACTATGGCGAGCGGCGTCGGCTTGCTGGCTCGCGTCGCTCAGT
4,400 4,410 4,420 4,430 4,440 4,450 4,460 4,470 4,480

BspQI
SapI BsaXI PvuII
GTGAGCGAGGAAGCGGAAGAGCGCCCAATACGCAAACCGCCTCTCCCCGCGCGTTGGCCGATTCATTAATGCAGCTGGCACGA
CACTCGCTCCTTCGCCTTCTCGCGGGTTATGCGTTTGGCGGAGAGGGGCGCGCAACCGGCTAAGTAATTACGTCGACCGTGCT
4,490 4,500 4,510 4,520 4,530 4,540 4,550 4,560

CAGGTTTCCCGACTGGAAAGCGGGCAGTGAGCGCAACGCAATTAATGTGAGTTAGCTCACTCATTAGGCACCCCAGGCTTTAC
GTCCAAAGGGCTGACCTTTCGCCCGTCACTCGCGTTGCGTTAATTACACTCAATCGAGTGAGTAATCCGTGGGGTCCGAAATG
CAP binding site
4,570 4,580 4,590 4,600 4,610 4,620 4,630 4,640

ACTTTATGCTTCCGGCTCGTATGTTGTGTGGAATTGTGAGCGGATAACAATTTCACACAGGAAACAGCTATGACCATGATTAC
TGAAATACGAAGGCCGAGCATACAACACACCTTAACACTCGCCTATTGTTAAAGTGTGTCCTTTGTCGATACTGGTACTAATG
lac promoter lac operator M13 rev
4,650 4,660 4,670 4,680 4,690 4,700 4,710 4,720 4,730

PflFI
Tth111I
HindIII PpuMI

GCCAAGCTTACCAGTTCTCACACGGAACACCACTAATGGACACAAATTCGAAATACTTTGACCCTATTTTCGAGGACCTTGTC
CGGTTCGAATGGTCAAGAGTGTGCCTTGTGGTGATTACCTGTGTTTAAGCTTTATGAAACTGGGATAAAAGCTCCTGGAACAG

4,740 4,750 4,760 4,770 4,780 4,790 4,800 4,810

SwaI BsiWI

ACCTTGAGCCCAAGAGAGCCAAGATTTAAATTTTCCTATGACTTGATGCAAATTCCCAAAGCTAATAACATGCAAGACACGTA
TGGAACTCGGGTTCTCTCGGTTCTAAATTTAAAAGGATACTGAACTACGTTTAAGGGTTTCGATTATTGTACGTTCTGTGCAT

4,820 4,830 4,840 4,850 4,860 4,870 4,880 4,890

CGGTCAAGAAGACATATTTGACCTCTTAACAGGTTCAGACGCGACTGCCTCATCAGTAAGACCCGTTGAAAAGAACTTACCTG
GCCAGTTCTTCTGTATAAACTGGAGAATTGTCCAAGTCTGCGCTGACGGAGTAGTCATTCTGGGCAACTTTTCTTGAATGGAC

4,900 4,910 4,920 4,930 4,940 4,950 4,960 4,970 4,980

AAAAAAACGAATATATACTAGCGTTGAATGTTAGCGTCAACAACAAGAAGTTTAATGACGCGGAGGCCAAGGCAAAAAGATTC
TTTTTTTGCTTATATATGATCGCAACTTACAATCGCAGTTGTTGTTCTTCAAATTACTGCGCCTCCGGTTCCGTTTTTCTAAG

4,990 5,000 5,010 5,020 5,030 5,040 5,050 5,060

CTTGATTACGTAAGGGAGTTAGAATCATTTTGAATAAAAAACACGCTTTTTCAGTTCGAGTTTATCATTATCAATACTGCCAT
GAACTAATGCATTCCCTCAATCTTAGTAAAACTTATTTTTTGTGCGAAAAAGTCAAGCTCAAATAGTAATAGTTATGACGGTA

GAP promoter

5,070 5,080 5,090 5,100 5,110 5,120 5,130 5,140

TTCAAAGAATACGTAAATAATTAATAGTAGTGATTTTCCTAACTTTATTTAGTCAAAAAATTAGCCTTTTAATTCTGCTGTAA
AAGTTTCTTATGCATTTATTAATTATCATCACTAAAAGGATTGAAATAAATCAGTTTTTTAATCGGAAAATTAAGACGACATT

GAP promoter

5,150 5,160 5,170 5,180 5,190 5,200 5,210 5,220

CCCGTACATGCCCAAAATAGGGGGCGGGTTACACAGAATATATAACATCGTAGGTGTCTGGGTGAACAGTTTATTCCTGGCAT
GGGCATGTACGGGTTTTATCCCCCGCCCAATGTGTCTTATATATTGTAGCATCCACAGACCCACTTGTCAAATAAGGACCGTA

GAP promoter

5,230 5,240 5,250 5,260 5,270 5,280 5,290 5,300 5,310

CCACTAAATATAATGGAGCCCGCTTTTTAAGCTGGCATCCAGAAAAAAAAAGAATCCCAGCACCAAAATATTGTTTTCTTCAC
GGTGATTTATATTACCTCGGGCGAAAAATTCGACCGTAGGTCTTTTTTTTTCTTAGGGTCGTGGTTTTATAACAAAAGAAGTG

GAP promoter

5,320 5,330 5,340 5,350 5,360 5,370 5,380 5,390

```
CAACCATCAGTTCATAGGTCCATTCTCTTAGCGCAACTACAGAGAACAGGGGCACAAACAGGCAAAAAACGGGCACAACCTCA
GTTGGTAGTCAAGTATCCAGGTAAGAGAATCGCGTTGATGTCTCTTGTCCCCGTGTTTGTCCGTTTTTTGCCCGTGTTGGAGT
GAP promoter
5,400 5,410 5,420 5,430 5,440 5,450 5,460 5,470

BspMI
BfuAI                  BaeI
ATGGAGTGATGCAACCTGCCTGGAGTAAATGATGACACAAGGCAATTGACCCACGCATGTATCTATCTCATTTTCTTACACCT
TACCTCACTACGTTGGACGGACCTCATTTACTACTGTGTTCCGTTAACTGGGTGCGTACATAGATAGAGTAAAAGAATGTGGA
GAP promoter
5,480 5,490 5,500 5,510 5,520 5,530 5,540 5,550 5,560

TCTATTACCTTCTGCTCTCTCTGATTTGGAAAAAGCTGAAAAAAAAGGTTGAAACCAGTTCCCTGAAATTATTCCCCTACTTG
AGATAATGGAAGACGAGAGAGACTAAACCTTTTTCGACTTTTTTTTCCAACTTTGGTCAAGGGACTTTAATAAGGGGATGAAC
GAP promoter
5,570 5,580 5,590 5,600 5,610 5,620 5,630 5,640

ACTAATAAGTATATAAAGACGGTAGGTATTGATTGTAATTCTGTAAATCTATTTCTTAAACTTCTTAAATTCTACTTTTATAG
TGATTATTCATATATTTCTGCCATCCATAACTAACATTAAGACATTTAGATAAAGAATTTGAAGAATTTAAGATGAAAATATC
GAP promoter
5,650 5,660 5,670 5,680 5,690 5,700 5,710 5,720

SmaI
TspMI
BsoBI
XmaI
AvaI
TTAGTCTTTTTTTTAGTTTTAAAACACCAAGAACTTAGTTTCGAATAAACACACATAAACACCCGGGATGAGATTTCCTTCAA
AATCAGAAAAAAAATCAAAATTTTGTGGTTCTTGAATCAAAGCTTATTTGTGTGTATTTGTGGGCCCTACTCTAAAGGAAGTT
GAP promoter
5,730 5,740 5,750 5,760 5,770 5,780 5,790 5,800 5,810

BstAPI PvuII
TTTTTACTGCTGTTGTTTTCGCAGCATCCTCCGCATTAGCTGCTCCAGCTAACACTACAGCTGAAGATGAAACGGCACAAATT
AAAAATGACGACAACAAAAGCGTCGTAGGAGGCGTAATCGACGAGGTCGATTGTGATGTCGACTTCTACTTTGCCGTGTTTAA
Alpha factor appS4
5,820 5,830 5,840 5,850 5,860 5,870 5,880 5,890

CCGGCTGAAGCTGTCATCGGTTACTTAGGTTTAGAAGGGGATTCTGATGTTGCTGCTTTGCCATTGTCCGATAGCACAAATAA
GGCCGACTTCGACAGTAGCCAATGAATCCAAATCTTCCCCTAAGACTACAACGACGAAACGGTAACAGGCTATCGTGTTTATT
Alpha factor appS4
5,900 5,910 5,920 5,930 5,940 5,950 5,960 5,970
```

CGGGTCATTGTCTACTAATACTACTATTGCCAGCATTGCTGCTAAAGAAGAAGGGGTATCTTTGGATAAAAGACATGGTGAAG
GCCCAGTAACAGATGATTATGATGATAACGGTCGTAACGACGATTTCTTCTTCCCCATAGAAACCTATTTTCTGTACCACTTC

Alpha factor appS4 | Ex4

5,980 5,990 6,000 6,010 6,020 6,030 6,040 6,050

GAACATTTACCAGTGACTTGTCAAAACAGATGGAAGAGGAGGCAGTGCGGTTATTTATTGAGTGGCTTAAGAACGGAGGACCA
CTTGTAAATGGTCACTGAACAGTTTTGTCTACCTTCTCCTCCGTCACGCCAATAAATAACTCACCGAATTCTTGCCTCCTGGT

Ex4

6,060 6,070 6,080 6,090 6,100 6,110 6,120 6,130 6,140

SalI

AGTAGCGGTGCACCTCCACCATCGTAAGTCGACTTGGTTGAACACGTTGCCAAGGCTTAAGTGAATTTACTTTAAATCTTGCA
TCATCGCCACGTGGAGGTGGTAGCATTCAGCTGAACCAACTTGTGCAACGGTTCCGAATTCACTTAAATGAAATTTAGAACGT

Ex4

6,150 6,160 6,170 6,180 6,190 6,200 6,210 6,220

SwaI

TTTAAATAAATTTTCTTTTTATAGCTTTATGACTTAGTTTCAATTTATATACTATTTTAATGACATTTTCGATTCATTGATTG
AAATTTATTTAAAAGAAAAATATCGAAATACTGAATCAAAGTTAAATATATGATAAAATTACTGTAAAAGCTAAGTAACTAAC

6,230 6,240 6,250 6,260 6,270 6,280 6,290 6,300

KpnI
Acc65I BtgZI

AGGTACCGCGATGTAGTAAAACTAGCTAGACCGAGAAAGAGACTAGAAATGCAAAAGGCACTTCTACAATGGCTGCCATCATT
TCCATGGCGCTACATCATTTTGATCGATCTGGCTCTTTCTCTGATCTTTACGTTTTCCGTGAAGATGTTACCGACGGTAGTAA

6,310 6,320 6,330 6,340 6,350 6,360 6,370 6,380 6,390

BsrGI

ATTATCCGATGTGACGCTGCATTTTTTTTTTTTTTTTTTTTTTTTTTTTTTTTTTTTTTTTTTTTTTTTTTGTACAAATATCATAA
TAATAGGCTACACTGCGACGTAAAAAAAAAAAAAAAAAAAAAAAAAAAAAAAAAAAAAAAAAAAAAAAAACATGTTTATAGTATT

6,400 6,410 6,420 6,430 6,440 6,450 6,460 6,470

AAAAAGAGAATCTTTTTAAGCAAGGATTTTCTTAACTTCTTCGGCGACAGCATCACCGACTTCGGTGGTACTGTTGGAACCAC
TTTTTCTCTTAGAAAAATTCGTTCCTAAAAGAATTGAAGAAGCCGCTGTCGTAGTGGCTGAAGCCACCATGACAACCTTGGTG

LEU2

6,480 6,490 6,500 6,510 6,520 6,530 6,540 6,550

BspMI
BfuAI
EcoNI

CTAAATCACCAGTTCTGATACCTGCATCCAAAACCTTTTTAACTGCATCTTCAATGGCCTTACCTTCTTCAGGCAAGTTCAAT
GATTTAGTGGTCAAGACTATGGACGTAGGTTTTGGAAAAATTGACGTAGAAGTTACCGGAATGGAAGAAGTCCGTTCAAGTTA

LEU2

6,560 6,570 6,580 6,590 6,600 6,610 6,620 6,630 6,640

GACAATTTCAACATCATTGCAGCAGACAAGATAGTGGCGATAGGGTCAACCTTATTCTTTGGCAAATCTGGAGCAGAACCGTG
CTGTTAAAGTTGTAGTAACGTCGTCTGTTCTATCACCGCTATCCCAGTTGGAATAAGAAACCGTTTAGACCTCGTCTTGGCAC

LEU2

6,650 6,660 6,670 6,680 6,690 6,700 6,710 6,720

GCATGGTTCGTACAAACCAAATGCGGTGTTCTTGTCTGGCAAAGAGGCCAAGGACGCAGATGGCAACAAACCCAAGGAACCTG
CGTACCAAGCATGTTTGGTTTACGCCACAAGAACAGACCGTTTCTCCGGTTCCTGCGTCTACCGTTGTTTGGGTTCCTTGGAC

LEU2

6,730 6,740 6,750 6,760 6,770 6,780 6,790 6,800

EcoRV
PsiI
BstXI

GGATAACGGAGGCTTCATCGGAGATGATATCACCAAACATGTTGCTGGTGATTATAATACCATTTAGGTGGGTTGGGTTCTTA
CCTATTGCCTCCGAAGTAGCCTCTACTATAGTGGTTTGTACAACGACCACTAATATTATGGTAAATCCACCCAACCCAAGAAT

LEU2

6,810 6,820 6,830 6,840 6,850 6,860 6,870 6,880

ACTAGGATCATGGCGGCAGAATCAATCAATTGATGTTGAACCTTCAATGTAGGAAATTCGTTCTTGATGGTTTCCTCCACAGT
TGATCCTAGTACCGCCGTCTTAGTTAGTTAACTACAACTTGGAAGTTACATCCTTTAAGCAAGAACTACCAAAGGAGGTGTCA

LEU2

6,890 6,900 6,910 6,920 6,930 6,940 6,950 6,960 6,970

TTTTCTCCATAATCTTGAAGAGGCCAAAACATTAGCTTTATCCAAGGACCAAATAGGCAATGGTGGCTCATGTTGTAGGGCCA
AAAAGAGGTATTAGAACTTCTCCGGTTTTGTAATCGAAATAGGTTCCTGGTTTATCCGTTACCACCGAGTACAACATCCCGGT

LEU2

6,980 6,990 7,000 7,010 7,020 7,030 7,040 7,050

TGAAAGCGGCCATTCTTGTGATTCTTTGCACTTCTGGAACGGTGTATTGTTCACTATCCCAAGCGACACCATCACCATCGTCT
ACTTTCGCCGGTAAGAACACTAAGAAACGTGAAGACCTTGCCACATAACAAGTGATAGGGTTCGCTGTGGTAGTGGTAGCAGA

LEU2

7,060 7,070 7,080 7,090 7,100 7,110 7,120 7,130

```
TCCTTTCTCTTACCAAAGTAAATACCTCCCACTAATTCTCTGACAACAACGAAGTCAGTACCTTTAGCAAATTGTGGCTTGAT
AGGAAAGAGAATGGTTTCATTTATGGAGGGTGATTAAGAGACTGTTGTTGCTTCAGTCATGGAAATCGTTTAACACCGAACTA
LEU2
7,140 7,150 7,160 7,170 7,180 7,190 7,200 7,210 7,220

TGGAGATAAGTCTAAAAGAGAGTCGGATGCAAAGTTACATGGTCTTAAGTTGGCGTACAATTGAAGTTCTTTACGGATTTTTA
ACCTCTATTCAGATTTTCTCTCAGCCTACGTTTCAATGTACCAGAATTCAACCGCATGTTAACTTCAAGAAATGCCTAAAAAT
LEU2
7,230 7,240 7,250 7,260 7,270 7,280 7,290 7,300

EcoNI PsrI
GTAAACCTTGTTCAGGTCTAACACTACCTGTACCCCATTTAGGACCACCCACAGCACCTAACAAAACGGCATCAACCTTCTTG
CATTTGGAACAAGTCCAGATTGTGATGGACATGGGGTAAATCCTGGTGGGTGTCGTGGATTGTTTTGCCGTAGTTGGAAGAAC
LEU2
7,310 7,320 7,330 7,340 7,350 7,360 7,370 7,380

BspDI
ClaI
BsmFI
GAGGCTTCCAGCGCCTCATCTGGAAGTGGGACACCTGTAGCATCGATAGCAGCACCACCAATTAAATGATTTTCGAAATCGAA
CTCCGAAGGTCGCGGAGTAGACCTTCACCCTGTGGACATCGTAGCTATCGTCGTGGTGGTTAATTTACTAAAAGCTTTAGCTT
LEU2
7,390 7,400 7,410 7,420 7,430 7,440 7,450 7,460 7,470

BstEII
CTTGACATTGGAACGAACATCAGAAATAGCTTTAAGAACCTTAATGGCTTCGGCTGTGATTTCTTGACCAACGTGGTCACCTG
GAACTGTAACCTTGCTTGTAGTCTTTATCGAAATTCTTGGAATTACCGAAGCCGACACTAAAGAACTGGTTGCACCAGTGGAC
LEU2
7,480 7,490 7,500 7,510 7,520 7,530 7,540 7,550

GCAAAACGACGATCTTCTTAGGGGCAGACATTACAATGGTATATCCTTGAAATATATATAAAAAAAAAAAAAAAAAAAAAAAA
CGTTTTGCTGCTAGAAGAATCCCCGTCTGTAATGTTACCATATAGGAACTTTATATATATTTTTTTTTTTTTTTTTTTTTTTT
LEU2
7,560 7,570 7,580 7,590 7,600 7,610 7,620 7,630

AAAAAAATGCAGCTTCTCAATGATATTCGAATACGCTTTGAGGAGATACAGCCTAATATCCGACAAACTGTTTTACAGATTTA
TTTTTTTACGTCGAAGAGTTACTATAAGCTTATGCGAAACTCCTCTATGTCGGATTATAGGCTGTTTGACAAAATGTCTAAAT
7,640 7,650 7,660 7,670 7,680 7,690 7,700 7,710
```

```
     PvuI
CGATCGTACTTGTTACCCATCATTGAATTTTGAACATCCGAACCTGGGAGTTTTCCCTGAAACAGATAGTATATTTGAACCTG
GCTAGCATGAACAATGGGTAGTAACTTAAAACTTGTAGGCTTGGACCCTCAAAAGGGACTTTGTCTATCATATAAACTTGGAC
7,720    7,730     7,740     7,750     7,760     7,770     7,780     7,790     7,800

                                                     PfoI                   BstAPI
TATAATAATATATAGTCTAGCGCTTTACGGAAGACAATGTATGTATTTCGGTTCCTGGAGAAACTATTGCATCTATTGCATAG
ATATTATTATATATCAGATCGCGAAATGCCTTCTGTTACATACATAAAGCCAAGGACCTCTTTGATAACGTAGATAACGTATC
      7,810     7,820     7,830     7,840     7,850     7,860     7,870     7,880

           BmgBI
GTAATCTTGCACGTCGCATCCCCGGTTCATTTTCTGCGTTTCCATCTTGCACTTCAATAGCATATCTTT
CATTAGAACGTGCAGCGTAGGGGCCAAGTAAAAGACGCAAAGGTAGAACGTGAAGTTATCGTATAGAAA
   7,890     7,900     7,910     7,920     7,930     7,940     7,950
```

pULS (7906 bp)

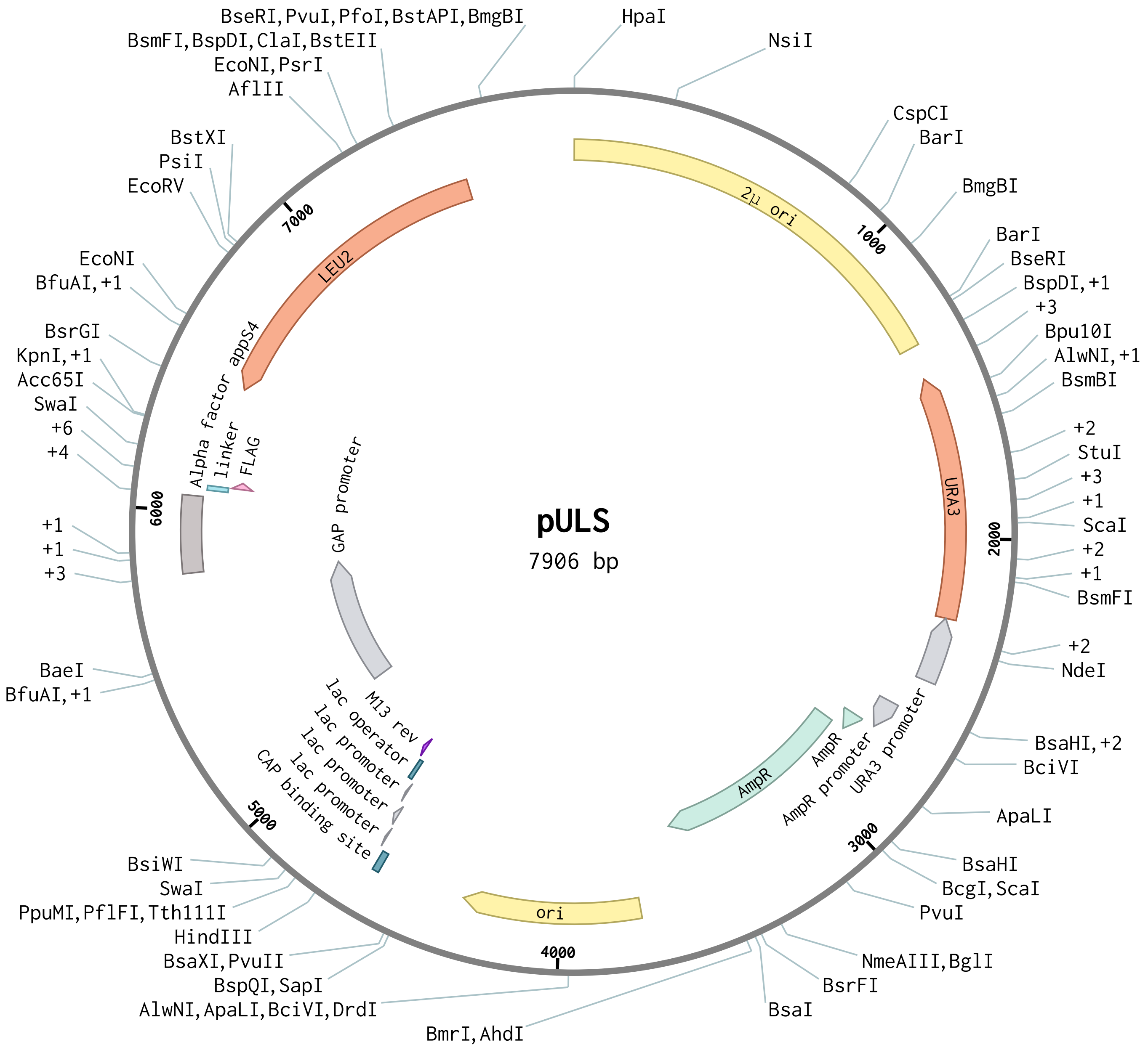
pULS
7906 bp
1000
2000
3000
4000
5000
6000
7000
2μ ori
URA3
URA3 promoter
AmpR promoter
AmpR
ori
CAP binding site
lac promoter
lac promoter
lac promoter
lac operator
M13 rev
GAP promoter
FLAG
linker
Alpha factor appS4
LEU2
HpaI
NsiI
CspCI
BarI
BmgBI
BarI
BseRI
BspDI,+1
+3
Bpu10I
AlwNI,+1
BsmBI
+2
StuI
+3
+1
ScaI
+2
+1
BsmFI
+2
NdeI
BsaHI,+2
BciVI
ApaLI
BsaHI
BcgI,ScaI
PvuI
NmeAIII,BglI
BsrFI
BsaI
BmrI,AhdI
AlwNI,ApaLI,BciVI,DrdI
BspQI,SapI
BsaXI,PvuII
HindIII
PpuMI,PflFI,Tth111I
SwaI
BsiWI
BfuAI,+1
BaeI
+3
+1
+1
+4
+6
SwaI
Acc65I
KpnI,+1
BsrGI
BfuAI,+1
EcoNI
EcoRV
PsiI
BstXI
AflII
EcoNI,PsrI
BsmFI,BspDI,ClaI,BstEII
BseRI,PvuI,PfoI,BstAPI,BmgBI

pULS (7906 bp)

HpaI

GTTAACGAAGCATCTGTGCTTCATTTTGTAGAACAAAAATGCAACGCGAGAGCGCTAATTTTTCAAACAAAGAATCTGAGCTGCATTTTTACAGAACAGAAATGCAA
CAATTGCTTCGTAGACACGAAGTAAAACATCTTGTTTTTACGTTGCGCTCTCGCGATTAAAAAGTTTGTTTCTTAGACTCGACGTAAAAATGTCTTGTCTTTACGTT

2µ ori

20 40 60 80 100

CGCGAAAGCGCTATTTTACCAACGAAGAATCTGTGCTTCATTTTTGTAAAACAAAAATGCAACGCGAGAGCGCTAATTTTTCAAACAAAGAATCTGAGCTGCATTTT
GCGCTTTCGCGATAAAATGGTTGCTTCTTAGACACGAAGTAAAAACATTTTGTTTTTACGTTGCGCTCTCGCGATTAAAAAGTTTGTTTCTTAGACTCGACGTAAAA

2µ ori

120 140 160 180 200

NsiI

TACAGAACAGAAATGCAACGCGAGAGCGCTATTTTACCAACAAAGAATCTATACTTCTTTTTTGTTCTACAAAAATGCATCCCGAGAGCGCTATTTTTCTAACAAAG
ATGTCTTGTCTTTACGTTGCGCTCTCGCGATAAAATGGTTGTTTCTTAGATATGAAGAAAAAACAAGATGTTTTTACGTAGGGCTCTCGCGATAAAAAGATTGTTTC

2µ ori

220 240 260 280 300 320

CATCTTAGATTACTTTTTTTCTCCTTTGTGCGCTCTATAATGCAGTCTCTTGATAACTTTTTGCACTGTAGGTCCGTTAAGGTTAGAAGAAGGCTACTTTGGTGTCT
GTAGAATCTAATGAAAAAAAGAGGAAACACGCGAGATATTACGTCAGAGAACTATTGAAAAACGTGACATCCAGGCAATTCCAATCTTCTTCCGATGAAACCACAGA

2µ ori

340 360 380 400 420

ATTTTCTCTTCCATAAAAAAAGCCTGACTCCACTTCCCGCGTTTACTGATTACTAGCGAAGCTGCGGGTGCATTTTTTCAAGATAAAGGCATCCCCGATTATATTCT
TAAAAGAGAAGGTATTTTTTTCGGACTGAGGTGAAGGGCGCAAATGACTAATGATCGCTTCGACGCCCACGTAAAAAAGTTCTATTTCCGTAGGGGCTAATATAAGA

2µ ori

440 460 480 500 520

ATACCGATGTGGATTGCGCATACTTTGTGAACAGAAAGTGATAGCGTTGATGATTCTTCATTGGTCAGAAAATTATGAACGGTTTCTTCTATTTTGTCTCTATATAC
TATGGCTACACCTAACGCGTATGAAACACTTGTCTTTCACTATCGCAACTACTAAGAAGTAACCAGTCTTTTAATACTTGCCAAAGAAGATAAAACAGAGATATATG

2µ ori

540 560 580 600 620 640

TACGTATAGGAAATGTTTACATTTTCGTATTGTTTTCGATTCACTCTATGAATAGTTCTTACTACAATTTTTTTGTCTAAAGAGTAATACTAGAGATAAACATAAAA
ATGCATATCCTTTACAAATGTAAAAGCATAACAAAAGCTAAGTGAGATACTTATCAAGAATGATGTTAAAAAAACAGATTTCTCATTATGATCTCTATTTGTATTTT

2µ ori

660 680 700 720 740

```
                                                                                                 CspCI
AATGTAGAGGTCGAGTTTAGATGCAAGTTCAAGGAGCGAAAGGTGGATGGGTAGGTTATATAGGGATATAGCACAGAGATATATAGCAAAGAGATACTTTTGAGCAA
TTACATCTCCAGCTCAAATCTACGTTCAAGTTCCTCGCTTTCCACCTACCCATCCAATATATCCCTATATCGTGTCTCTATATATCGTTTCTCTATGAAAACTCGTT
2µ ori
760 780 800 820 840

                                                                                                          BarI
TGTTTGTGGAAGCGGTATTCGCAATATTTTAGTAGCTCGTTACAGTCCGGTGCGTTTTTGGTTTTTTGAAAGTGCGTCTTCAGAGCGCTTTTGGTTTTCAAAAGCGC
ACAAACACCTTCGCCATAAGCGTTATAAAATCATCGAGCAATGTCAGGCCACGCAAAAACCAAAAAACTTTCACGCAGAAGTCTCGCGAAAACCAAAAGTTTTCGCG
2µ ori
860 880 900 920 940 960

TCTGAAGTTCCTATACTTTCTAGCTAGAGAATAGGAACTTCGGAATAGGAACTTCAAAGCGTTTCCGAAAACGAGCGCTTCCGAAAATGCAACGCGAGCTGCGCACA
AGACTTCAAGGATATGAAAGATCGATCTCTTATCCTTGAAGCCTTATCCTTGAAGTTTCGCAAAGGCTTTTGCTCGCGAAGGCTTTTACGTTGCGCTCGACGCGTGT
2µ ori
980 1,000 1,020 1,040 1,060

                BmgBI
TACAGCTCACTGTTCACGTCGCACCTATATCTGCGTGTTGCCTGTATATATATATACATGAGAAGAACGGCATAGTGCGTGTTTATGCTTAAATGCGTACTTATATG
ATGTCGAGTGACAAGTGCAGCGTGGATATAGACGCACAACGGACATATATATATATGTACTCTTCTTGCCGTATCACGCACAAATACGAATTTACGCATGAATATAC
2µ ori
1,080 1,100 1,120 1,140 1,160

                                                                                                   BarI
CGTCTATTTATGTAGGATGAAAGGTAGTCTAGTACCTCCTGTGATATTATCCCATTCCATGCGGGGTATCGTATGCTTCCTTCAGCACTACCCTTTAGCTGTTCTAT
GCAGATAAATACATCCTACTTTCCATCAGATCATGGAGGACACTATAATAGGGTAAGGTACGCCCCATAGCATACGAAGGAAGTCGTGATGGGAAATCGACAAGATA
2µ ori
1,180 1,200 1,220 1,240 1,260 1,280

                                                              BspDI
BseRI                                                         ClaI
ATGCTGCCACTCCTCAATTGGATTAGTCTCATCCTTCAATGCTATCATTTCCTTTGATATTGGATCGATCCGATGATAAGCTGTCAAACATGAGAATTGGGTAATAA
TACGACGGTGAGGAGTTAACCTAATCAGAGTAGGAAGTTACGATAGTAAAGGAAACTATAACCTAGCTAGGCTACTATTCGACAGTTTGTACTCTTAACCCATTATT
2µ ori
1,300 1,320 1,340 1,360 1,380

                                                NsiI
                                        BstZ17I        PsiI
CTGATATAATTAAATTGAAGCTCTAATTTGTGAGTTTAGTATACATGCATTTACTTATAATACAGTTTTTTAGTTTTGCTGGCCGCATCTTCTCAAATATGCTTCCC
GACTATATTAATTTAACTTCGAGATTAAACACTCAAATCATATGTACGTAAATGAATATTATGTCAAAAAATCAAAACGACCGGCGTAGAAGAGTTTATACGAAGGG
URA3
1,400 1,420 1,440 1,460 1,480
```

AlwNI
Bpu10I BsaI
AGCCTGCTTTTCTGTAACGTTCACCCTCTACCTTAGCATCCCTTCCCTTTGCAAATAGTCCTCTTCCAACAATAATAATGTCAGATCCTGTAGAGACCACATCATCC
TCGGACGAAAAGACATTGCAAGTGGGAGATGGAATCGTAGGGAAGGGAAACGTTTATCAGGAGAAGGTTGTTATTATTACAGTCTAGGACATCTCTGGTGTAGTAGG
URA3
1,500 1,520 1,540 1,560 1,580 1,600

BsmBI
ACGGTTCTATACTGTTGACCCAATGCGTCTCCCTTGTCATCTAAACCCACACCGGGTGTCATAATCAACCAATCGTAACCTTCATCTCTTCCACCCATGTCTCTTTG
TGCCAAGATATGACAACTGGGTTACGCAGAGGGAACAGTAGATTTGGGTGTGGCCCACAGTATTAGTTGGTTAGCATTGGAAGTAGAGAAGGTGGGTACAGAGAAAC
URA3
1,620 1,640 1,660 1,680 1,700

BspQI
SapI
AGCAATAAAGCCGATAACAAAATCTTTGTCGCTCTTCGCAATGTCAACAGTACCCTTAGTATATTCTCCAGTAGATAGGGAGCCCTTGCATGACAATTCTGCTAACA
TCGTTATTTCGGCTATTGTTTTAGAAACAGCGAGAAGCGTTACAGTTGTCATGGGAATCATATAAGAGGTCATCTATCCCTCGGGAACGTACTGTTAAGACGATTGT
URA3
1,720 1,740 1,760 1,780 1,800

ApaI
StuI PspOMI BsmI
TCAAAAGGCCTCTAGGTTCCTTTGTTACTTCTTCTGCCGCCTGCTTCAAACCGCTAACAATACCTGGGCCCACCACACCGTGTGCATTCGTAATGTCTGCCCATTCT
AGTTTTCCGGAGATCCAAGGAAACAATGAAGAAGACGGCGGACGAAGTTTGGCGATTGTTATGGACCCGGGTGGTGTGGCACACGTAAGCATTACAGACGGGTAAGA
URA3
1,820 1,840 1,860 1,880 1,900 1,920

BstZ17I ScaI
GCTATTCTGTATACACCCGCAGAGTACTGCAATTTGACTGTATTACCAATGTCAGCAAATTTTCTGTCTTCGAAGAGTAAAAAATTGTACTTGGCGGATAATGCCTT
CGATAAGACATATGTGGGCGTCTCATGACGTTAAACTGACATAATGGTTACAGTCGTTTAAAAGACAGAAGCTTCTCATTTTTTAACATGAACCGCCTATTACGGAA
URA3
1,940 1,960 1,980 2,000 2,020

NcoI EcoRV PpuMI BsmFI
TAGCGGCTTAACTGTGCCCTCCATGGAAAAATCAGTCAAGATATCCACATGTGTTTTTAGTAAACAAATTTTGGGACCTAATGCTTCAACTAACTCCAGTAATTCCT
ATCGCCGAATTGACACGGGAGGTACCTTTTTAGTCAGTTCTATAGGTGTACACAAAAATCATTTGTTTAAAACCCTGGATTACGAAGTTGATTGAGGTCATTAAGGA
URA3
2,040 2,060 2,080 2,100 2,120 2,140

TGGTGGTACGAACATCCAATGAAGCACACAAGTTTGTTTGCTTTTCGTGCATGATATTAAATAGCTTGGCAGCAACAGGACTAGGATGAGTAGCAGCACGTTCCTTA
ACCACCATGCTTGTAGGTTACTTCGTGTGTTCAAACAAACGAAAAGCACGTACTATAATTTATCGAACCGTCGTTGTCCTGATCCTACTCATCGTCGTGCAAGGAAT

URA3

2,160 2,180 2,200 2,220 2,240

BsgI BmrI NdeI

TATGTAGCTTTCGACATGATTTATCTTCGTTTCCTGCATGTTTTTGTTCTGTGCAGTTGGGTTAAGAATACTGGGCAATTTCATGTTTCTTCAACACTACATATGCG
ATACATCGAAAGCTGTACTAAATAGAAGCAAAGGACGTACAAAAACAAGACACGTCAACCCAATTCTTATGACCCGTTAAAGTACAAAGAAGTTGTGATGTATACGC

URA3 | URA3 promoter

2,260 2,280 2,300 2,320 2,340

TATATATACCAATCTAAGTCTGTGCTCCTTCCTTCGTTCTTCCTTCTGTTCGGAGATTACCGAATCAAAAAAATTTCAAAGAAACCGAAATCAAAAAAAAGAATAAA
ATATATATGGTTAGATTCAGACACGAGGAAGGAAGCAAGAAGGAAGACAAGCCTCTAATGGCTTAGTTTTTTTAAAGTTTCTTTGGCTTTAGTTTTTTTTCTTATTT

URA3 promoter

2,360 2,380 2,400 2,420 2,440 2,460

ZraI
BsaHI

AAAAAAATGATGAATTGAATTGAAAAGCTAATTCTTGAAGACGAAAGGGCCTCGTGATACGCCTATTTTTATAGGTTAATGTCATGATAATAATGGTTTCTTAGACG
TTTTTTTACTACTTAACTTAACTTTTCGATTAAGAACTTCTGCTTTCCCGGAGCACTATGCGGATAAAAATATCCAATTACAGTACTATTATTACCAAAGAATCTGC

URA3 promoter

2,480 2,500 2,520 2,540 2,560

AatII BciVI

TCAGGTGGCACTTTTCGGGGAAATGTGCGCGGAACCCCTATTTGTTTATTTTTCTAAATACATTCAAATATGTATCCGCTCATGAGACAATAACCCTGATAAATGCT
AGTCCACCGTGAAAAGCCCCTTTACACGCGCCTTGGGGATAAACAAATAAAAAGATTTATGTAAGTTTATACATAGGCGAGTACTCTGTTATTGGGACTATTTACGA

AmpR promoter

2,580 2,600 2,620 2,640 2,660

TCAATAATATTGAAAAAGGAAGAGTATGAGTATTCAACATTTCCGTGTCGCCCTTATTCCCTTTTTTGCGGCATTTTGCCTTCCTGTTTTTGCTCACCCAGAAACGC
AGTTATTATAACTTTTTCCTTCTCATACTCATAAGTTGTAAAGGCACAGCGGGAATAAGGGAAAAAACGCCGTAAAACGGAAGGACAAAAACGAGTGGGTCTTTGCG

AmpR promoter | AmpR | AmpR

2,680 2,700 2,720 2,740 2,760 2,780

ApaLI

TGGTGAAAGTAAAAGATGCTGAAGATCAGTTGGGTGCACGAGTGGGTTACATCGAACTGGATCTCAACAGCGGTAAGATCCTTGAGAGTTTTCGCCCCGAAGAACGT
ACCACTTTCATTTTCTACGACTTCTAGTCAACCCACGTGCTCACCCAATGTAGCTTGACCTAGAGTTGTCGCCATTCTAGGAACTCTCAAAAGCGGGGCTTCTTGCA

AmpR

2,800 2,820 2,840 2,860 2,880

BsaHI BcgI

TTTCCAATGATGAGCACTTTTAAAGTTCTGCTATGTGGCGCGGTATTATCCCGTATTGACGCCGGGCAAGAGCAACTCGGTCGCCGCATACACTATTCTCAGAATGA
AAAGGTTACTACTCGTGAAAATTTCAAGACGATACACCGCGCCATAATAGGGCATAACTGCGGCCCGTTCTCGTTGAGCCAGCGGCGTATGTGATAAGAGTCTTACT

AmpR

2,900 2,920 2,940 2,960 2,980

ScaI

CTTGGTTGAGTACTCACCAGTCACAGAAAAGCATCTTACGGATGGCATGACAGTAAGAGAATTATGCAGTGCTGCCATAACCATGAGTGATAACACTGCGGCCAACT
GAACCAACTCATGAGTGGTCAGTGTCTTTTCGTAGAATGCCTACCGTACTGTCATTCTCTTAATACGTCACGACGGTATTGGTACTCACTATTGTGACGCCGGTTGA

AmpR

3,000 3,020 3,040 3,060 3,080 3,100

PvuI

TACTTCTGACAACGATCGGAGGACCGAAGGAGCTAACCGCTTTTTTGCACAACATGGGGGATCATGTAACTCGCCTTGATCGTTGGGAACCGGAGCTGAATGAAGCC
ATGAAGACTGTTGCTAGCCTCCTGGCTTCCTCGATTGGCGAAAAAACGTGTTGTACCCCCTAGTACATTGAGCGGAACTAGCAACCCTTGGCCTCGACTTACTTCGG

AmpR

3,120 3,140 3,160 3,180 3,200

ATACCAAACGACGAGCGTGACACCACGATGCCTGTAGCAATGGCAACAACGTTGCGCAAACTATTAACTGGCGAACTACTTACTCTAGCTTCCCGGCAACAATTAAT
TATGGTTTGCTGCTCGCACTGTGGTGCTACGGACATCGTTACCGTTGTTGCAACGCGTTTGATAATTGACCGCTTGATGAATGAGATCGAAGGGCCGTTGTTAATTA

AmpR

3,220 3,240 3,260 3,280 3,300

NmeAIII BglI BsrFI BsaI

AGACTGGATGGAGGCGGATAAAGTTGCAGGACCACTTCTGCGCTCGGCCCTTCCGGCTGGCTGGTTTATTGCTGATAAATCTGGAGCCGGTGAGCGTGGGTCTCGCG
TCTGACCTACCTCCGCCTATTTCAACGTCCTGGTGAAGACGCGAGCCGGGAAGGCCGACCGACCAAATAACGACTATTTAGACCTCGGCCACTCGCACCCAGAGCGC

AmpR

3,320 3,340 3,360 3,380 3,400 3,420

BmrI AhdI

GTATCATTGCAGCACTGGGGCCAGATGGTAAGCCCTCCCGTATCGTAGTTATCTACACGACGGGGAGTCAGGCAACTATGGATGAACGAAATAGACAGATCGCTGAG
CATAGTAACGTCGTGACCCCGGTCTACCATTCGGGAGGGCATAGCATCAATAGATGTGCTGCCCCTCAGTCCGTTGATACCTACTTGCTTTATCTGTCTAGCGACTC

AmpR

3,440 3,460 3,480 3,500 3,520

ATAGGTGCCTCACTGATTAAGCATTGGTAACTGTCAGACCAAGTTTACTCATATATACTTTAGATTGATTTAAAACTTCATTTTTAATTTAAAAGGATCTAGGTGAA
TATCCACGGAGTGACTAATTCGTAACCATTGACAGTCTGGTTCAAATGAGTATATATGAAATCTAACTAAATTTTGAAGTAAAAATTAAATTTTCCTAGATCCACTT

AmpR

3,540 3,560 3,580 3,600 3,620

GATCCTTTTTGATAATCTCATGACCAAAATCCCTTAACGTGAGTTTTCGTTCCACTGAGCGTCAGACCCCGTAGAAAAGATCAAAGGATCTTCTTGAGATCCTTTTT
CTAGGAAAAACTATTAGAGTACTGGTTTTAGGGAATTGCACTCAAAAGCAAGGTGACTCGCAGTCTGGGGCATCTTTTCTAGTTTCCTAGAAGAACTCTAGGAAAAA
ori
3,640 3,660 3,680 3,700 3,720 3,740

TTCTGCGCGTAATCTGCTGCTTGCAAACAAAAAAACCACCGCTACCAGCGGTGGTTTGTTTGCCGGATCAAGAGCTACCAACTCTTTTTCCGAAGGTAACTGGCTTC
AAGACGCGCATTAGACGACGAACGTTTGTTTTTTTGGTGGCGATGGTCGCCACCAAACAAACGGCCTAGTTCTCGATGGTTGAGAAAAAGGCTTCCATTGACCGAAG
ori
3,760 3,780 3,800 3,820 3,840

AGCAGAGCGCAGATACCAAATACTGTCCTTCTAGTGTAGCCGTAGTTAGGCCACCACTTCAAGAACTCTGTAGCACCGCCTACATACCTCGCTCTGCTAATCCTGTT
TCGTCTCGCGTCTATGGTTTATGACAGGAAGATCACATCGGCATCAATCCGGTGGTGAAGTTCTTGAGACATCGTGGCGGATGTATGGAGCGAGACGATTAGGACAA
ori
3,860 3,880 3,900 3,920 3,940

AlwNI ApaLI
ACCAGTGGCTGCTGCCAGTGGCGATAAGTCGTGTCTTACCGGGTTGGACTCAAGACGATAGTTACCGGATAAGGCGCAGCGGTCGGGCTGAACGGGGGGTTCGTGCA
TGGTCACCGACGACGGTCACCGCTATTCAGCACAGAATGGCCCAACCTGAGTTCTGCTATCAATGGCCTATTCCGCGTCGCCAGCCCGACTTGCCCCCCAAGCACGT
ori
3,960 3,980 4,000 4,020 4,040 4,060

CACAGCCCAGCTTGGAGCGAACGACCTACACCGAACTGAGATACCTACAGCGTGAGCTATGAGAAAGCGCCACGCTTCCCGAAGGGAGAAAGGCGGACAGGTATCCG
GTGTCGGGTCGAACCTCGCTTGCTGGATGTGGCTTGACTCTATGGATGTCGCACTCGATACTCTTTCGCGGTGCGAAGGGCTTCCCTCTTTCCGCCTGTCCATAGGC
ori
4,080 4,100 4,120 4,140 4,160

BciVI DrdI
GTAAGCGGCAGGGTCGGAACAGGAGAGCGCACGAGGGAGCTTCCAGGGGGAAACGCCTGGTATCTTTATAGTCCTGTCGGGTTTCGCCACCTCTGACTTGAGCGTCG
CATTCGCCGTCCCAGCCTTGTCCTCTCGCGTGCTCCCTCGAAGGTCCCCCTTTGCGGACCATAGAAATATCAGGACAGCCCAAAGCGGTGGAGACTGAACTCGCAGC
ori
4,180 4,200 4,220 4,240 4,260 4,280

ATTTTTGTGATGCTCGTCAGGGGGGCGGAGCCTATGGAAAAACGCCAGCAACGCGGCCTTTTTACGGTTCCTGGCCTTTTGCTGGCCTTTTGCTCACATGTTCTTTC
TAAAAACACTACGAGCAGTCCCCCCGCCTCGGATACCTTTTTGCGGTCGTTGCGCCGGAAAAATGCCAAGGACCGGAAAACGACCGGAAAACGAGTGTACAAGAAAG
ori
4,300 4,320 4,340 4,360 4,380

SapI
BspQI
CTGCGTTATCCCCTGATTCTGTGGATAACCGTATTACCGCCTTTGAGTGAGCTGATACCGCTCGCCGCAGCCGAACGACCGAGCGCAGCGAGTCAGTGAGCGAGGAA
GACGCAATAGGGGACTAAGACACCTATTGGCATAATGGCGGAAACTCACTCGACTATGGCGAGCGGCGTCGGCTTGCTGGCTCGCGTCGCTCAGTCACTCGCTCCTT
4,400 4,420 4,440 4,460 4,480

BsaXI PvuII

GCGGAAGAGCGCCCAATACGCAAACCGCCTCTCCCCGCGCGTTGGCCGATTCATTAATGCAGCTGGCACGACAGGTTTCCCGACTGGAAAGCGGGCAGTGAGCGCAA
CGCCTTCTCGCGGGTTATGCGTTTGGCGGAGAGGGGCGCGCAACCGGCTAAGTAATTACGTCGACCGTGCTGTCCAAAGGGCTGACCTTTCGCCCGTCACTCGCGTT

4,500 4,520 4,540 4,560 4,580 4,600

CGCAATTAATGTGAGTTAGCTCACTCATTAGGCACCCCAGGCTTTACACTTTATGCTTCCGGCTCGTATGTTGTGTGGAATTGTGAGCGGATAACAATTTCACACAG
GCGTTAATTACACTCAATCGAGTGAGTAATCCGTGGGGTCCGAAATGTGAAATACGAAGGCCGAGCATACAACACACCTTAACACTCGCCTATTGTTAAAGTGTGTC

CAP binding site lac promoter lac operator

4,620 4,640 4,660 4,680 4,700

PflFI
Tth111I
HindIII PpuMI

GAAACAGCTATGACCATGATTACGCCAAGCTTACCAGTTCTCACACGGAACACCACTAATGGACACAAATTCGAAATACTTTGACCCTATTTTCGAGGACCTTGTCA
CTTTGTCGATACTGGTACTAATGCGGTTCGAATGGTCAAGAGTGTGCCTTGTGGTGATTACCTGTGTTTAAGCTTTATGAAACTGGGATAAAAGCTCCTGGAACAGT

M13 rev

4,720 4,740 4,760 4,780 4,800

SwaI BsiWI

CCTTGAGCCCAAGAGAGCCAAGATTTAAATTTTCCTATGACTTGATGCAAATTCCCAAAGCTAATAACATGCAAGACACGTACGGTCAAGAAGACATATTTGACCTC
GGAACTCGGGTTCTCTCGGTTCTAAATTTAAAAGGATACTGAACTACGTTTAAGGGTTTCGATTATTGTACGTTCTGTGCATGCCAGTTCTTCTGTATAAACTGGAG

4,820 4,840 4,860 4,880 4,900 4,920

TTAACAGGTTCAGACGCGACTGCCTCATCAGTAAGACCCGTTGAAAAGAACTTACCTGAAAAAAACGAATATATACTAGCGTTGAATGTTAGCGTCAACAACAAGAA
AATTGTCCAAGTCTGCGCTGACGGAGTAGTCATTCTGGGCAACTTTTCTTGAATGGACTTTTTTTGCTTATATATGATCGCAACTTACAATCGCAGTTGTTGTTCTT

4,940 4,960 4,980 5,000 5,020

GTTTAATGACGCGGAGGCCAAGGCAAAAAGATTCCTTGATTACGTAAGGGAGTTAGAATCATTTTGAATAAAAAACACGCTTTTTCAGTTCGAGTTTATCATTATCA
CAAATTACTGCGCCTCCGGTTCCGTTTTTCTAAGGAACTAATGCATTCCCTCAATCTTAGTAAAACTTATTTTTTGTGCGAAAAAGTCAAGCTCAAATAGTAATAGT

5,040 5,060 5,080 5,100 5,120

ATACTGCCATTTCAAAGAATACGTAAATAATTAATAGTAGTGATTTTCCTAACTTTATTTAGTCAAAAAATTAGCCTTTTAATTCTGCTGTAACCCGTACATGCCCA
TATGACGGTAAAGTTTCTTATGCATTTATTAATTATCATCACTAAAAGGATTGAAATAAATCAGTTTTTTAATCGGAAAATTAAGACGACATTGGGCATGTACGGGT

GAP promoter

5,140 5,160 5,180 5,200 5,220 5,240

AAATAGGGGGCGGGTTACACAGAATATATAACATCGTAGGTGTCTGGGTGAACAGTTTATTCCTGGCATCCACTAAATATAATGGAGCCCGCTTTTTAAGCTGGCAT
TTTATCCCCCGCCCAATGTGTCTTATATATTGTAGCATCCACAGACCCACTTGTCAAATAAGGACCGTAGGTGATTTATATTACCTCGGGCGAAAAATTCGACCGTA

GAP promoter

5,260 5,280 5,300 5,320 5,340

CCAGAAAAAAAAAGAATCCCAGCACCAAAATATTGTTTTCTTCACCAACCATCAGTTCATAGGTCCATTCTCTTAGCGCAACTACAGAGAACAGGGGCACAAACAGG
GGTCTTTTTTTTTCTTAGGGTCGTGGTTTTATAACAAAAGAAGTGGTTGGTAGTCAAGTATCCAGGTAAGAGAATCGCGTTGATGTCTCTTGTCCCCGTGTTTGTCC

GAP promoter

5,360 5,380 5,400 5,420 5,440

BspMI BfuAI BaeI

CAAAAAACGGGCACAACCTCAATGGAGTGATGCAACCTGCCTGGAGTAAATGATGACACAAGGCAATTGACCCACGCATGTATCTATCTCATTTTCTTACACCTTCT
GTTTTTTGCCCGTGTTGGAGTTACCTCACTACGTTGGACGGACCTCATTTACTACTGTGTTCCGTTAACTGGGTGCGTACATAGATAGAGTAAAAGAATGTGGAAGA

GAP promoter

5,460 5,480 5,500 5,520 5,540 5,560

ATTACCTTCTGCTCTCTCTGATTTGGAAAAAGCTGAAAAAAAAGGTTGAAACCAGTTCCCTGAAATTATTCCCCTACTTGACTAATAAGTATATAAAGACGGTAGGT
TAATGGAAGACGAGAGAGACTAAACCTTTTTCGACTTTTTTTTCCAACTTTGGTCAAGGGACTTTAATAAGGGGATGAACTGATTATTCATATATTTCTGCCATCCA

GAP promoter

5,580 5,600 5,620 5,640 5,660

ATTGATTGTAATTCTGTAAATCTATTTCTTAAACTTCTTAAATTCTACTTTTATAGTTAGTCTTTTTTTTAGTTTTAAAACACCAAGAACTTAGTTTCGAATAAACA
TAACTAACATTAAGACATTTAGATAAAGAATTTGAAGAATTTAAGATGAAAATATCAATCAGAAAAAAAATCAAAATTTTGTGGTTCTTGAATCAAAGCTTATTTGT

GAP promoter

5,680 5,700 5,720 5,740 5,760

SmaI TspMI XmaI BstAPI PvuII

CACATAAACACCCGGGATGAGATTTCCTTCAATTTTTACTGCTGTTGTTTTCGCAGCATCCTCCGCATTAGCTGCTCCAGCTAACACTACAGCTGAAGATGAAACGG
GTGTATTTGTGGGCCCTACTCTAAAGGAAGTTAAAAATGACGACAACAAAAGCGTCGTAGGAGGCGTAATCGACGAGGTCGATTGTGATGTCGACTTCTACTTTGCC

Alpha factor appS4

5,780 5,800 5,820 5,840 5,860 5,880

CACAAATTCCGGCTGAAGCTGTCATCGGTTACTTAGGTTTAGAAGGGGATTCTGATGTTGCTGCTTTGCCATTGTCCGATAGCACAAATAACGGGTCATTGTCTACT
GTGTTTAAGGCCGACTTCGACAGTAGCCAATGAATCCAAATCTTCCCCTAAGACTACAACGACGAAACGGTAACAGGCTATCGTGTTTATTGCCCAGTAACAGATGA

Alpha factor appS4

5,900 5,920 5,940 5,960 5,980

BamHI EcoRI XbaI PstI

AATACTACTATTGCCAGCATTGCTGCTAAAGAAGAAGGGGTATCTTTGGATAAAAGAGAATTCGGATCCTCTAGACTGCAGGGTGGTGGAGGATCTGATTACAAGGA
TTATGATGATAACGGTCGTAACGACGATTTCTTCTTCCCCATAGAAACCTATTTTCTCTTAAGCCTAGGAGATCTGACGTCCCACCACCTCCTAGACTAATGTTCCT

Alpha factor appS4 linker FLAG

6,000 6,020 6,040 6,060 6,080

PaeR7I
PspXI
XhoI
TliI SalI AflII SwaI

TGACGATGACAAGTAACTCGAGGTCGACTTGGTTGAACACGTTGCCAAGGCTTAAGTGAATTTACTTTAAATCTTGCATTTAAATAAATTTTCTTTTTATAGCTTTA
ACTGCTACTGTTCATTGAGCTCCAGCTGAACCAACTTGTGCAACGGTTCCGAATTCACTTAAATGAAATTTAGAACGTAAATTTATTTAAAAGAAAAATATCGAAAT

FLAG

6,100 6,120 6,140 6,160 6,180 6,200

KpnI
Acc65I BtgZI

TGACTTAGTTTCAATTTATATACTATTTTAATGACATTTTCGATTCATTGATTGAGGTACCGCGATGTAGTAAAACTAGCTAGACCGAGAAAGAGACTAGAAATGCA
ACTGAATCAAAGTTAAATATATGATAAAATTACTGTAAAAGCTAAGTAACTAACTCCATGGCGCTACATCATTTTGATCGATCTGGCTCTTTCTCTGATCTTTACGT

6,220 6,240 6,260 6,280 6,300

BsrGI

AAAGGCACTTCTACAATGGCTGCCATCATTATTATCCGATGTGACGCTGCATTTTTTTTTTTTTTTTTTTTTTTTTTTTTTTTTTTTTTTTTTTTTTTTTGTACAAATA
TTTCCGTGAAGATGTTACCGACGGTAGTAATAATAGGCTACACTGCGACGTAAAAAAAAAAAAAAAAAAAAAAAAAAAAAAAAAAAAAAAAAAAAAAAAACATGTTTAT

6,320 6,340 6,360 6,380 6,400 6,420

TCATAAAAAAAGAGAATCTTTTTAAGCAAGGATTTTCTTAACTTCTTCGGCGACAGCATCACCGACTTCGGTGGTACTGTTGGAACCACCTAAATCACCAGTTCTGA
AGTATTTTTTTCTCTTAGAAAAATTCGTTCCTAAAAGAATTGAAGAAGCCGCTGTCGTAGTGGCTGAAGCCACCATGACAACCTTGGTGGATTTAGTGGTCAAGACT

LEU2

6,440 6,460 6,480 6,500 6,520

BspMI
BfuAI EcoNI

TACCTGCATCCAAAACCTTTTTAACTGCATCTTCAATGGCCTTACCTTCTTCAGGCAAGTTCAATGACAATTTCAACATCATTGCAGCAGACAAGATAGTGGCGATA
ATGGACGTAGGTTTTGGAAAAATTGACGTAGAAGTTACCGGAATGGAAGAAGTCCGTTCAAGTTACTGTTAAAGTTGTAGTAACGTCGTCTGTTCTATCACCGCTAT

LEU2

6,540 6,560 6,580 6,600 6,620

GGGTCAACCTTATTCTTTGGCAAATCTGGAGCAGAACCGTGGCATGGTTCGTACAAACCAAATGCGGTGTTCTTGTCTGGCAAAGAGGCCAAGGACGCAGATGGCAA
CCCAGTTGGAATAAGAAACCGTTTAGACCTCGTCTTGGCACCGTACCAAGCATGTTTGGTTTACGCCACAAGAACAGACCGTTTCTCCGGTTCCTGCGTCTACCGTT

LEU2

6,640 6,660 6,680 6,700 6,720 6,740

EcoRV PsiI BstXI

CAAACCCAAGGAACCTGGGATAACGGAGGCTTCATCGGAGATGATATCACCAAACATGTTGCTGGTGATTATAATACCATTTAGGTGGGTTGGGTTCTTAACTAGGA
GTTTGGGTTCCTTGGACCCTATTGCCTCCGAAGTAGCCTCTACTATAGTGGTTTGTACAACGACCACTAATATTATGGTAAATCCACCCAACCCAAGAATTGATCCT

LEU2

6,760 6,780 6,800 6,820 6,840

TCATGGCGGCAGAATCAATCAATTGATGTTGAACCTTCAATGTAGGAAATTCGTTCTTGATGGTTTCCTCCACAGTTTTTCTCCATAATCTTGAAGAGGCCAAAACA
AGTACCGCCGTCTTAGTTAGTTAACTACAACTTGGAAGTTACATCCTTTAAGCAAGAACTACCAAAGGAGGTGTCAAAAAGAGGTATTAGAACTTCTCCGGTTTTGT
LEU2
6,860 6,880 6,900 6,920 6,940

TTAGCTTTATCCAAGGACCAAATAGGCAATGGTGGCTCATGTTGTAGGGCCATGAAAGCGGCCATTCTTGTGATTCTTTGCACTTCTGGAACGGTGTATTGTTCACT
AATCGAAATAGGTTCCTGGTTTATCCGTTACCACCGAGTACAACATCCCGGTACTTTCGCCGGTAAGAACACTAAGAAACGTGAAGACCTTGCCACATAACAAGTGA
LEU2
6,960 6,980 7,000 7,020 7,040 7,060

ATCCCAAGCGACACCATCACCATCGTCTTCCTTTCTCTTACCAAAGTAAATACCTCCCACTAATTCTCTGACAACAACGAAGTCAGTACCTTTAGCAAATTGTGGCT
TAGGGTTCGCTGTGGTAGTGGTAGCAGAAGGAAAGAGAATGGTTTCATTTATGGAGGGTGATTAAGAGACTGTTGTTGCTTCAGTCATGGAAATCGTTTAACACCGA
LEU2
7,080 7,100 7,120 7,140 7,160

AflII EcoNI
TGATTGGAGATAAGTCTAAAAGAGAGTCGGATGCAAAGTTACATGGTCTTAAGTTGGCGTACAATTGAAGTTCTTTACGGATTTTTAGTAAACCTTGTTCAGGTCTA
ACTAACCTCTATTCAGATTTTCTCTCAGCCTACGTTTCAATGTACCAGAATTCAACCGCATGTTAACTTCAAGAAATGCCTAAAAATCATTTGGAACAAGTCCAGAT
LEU2
7,180 7,200 7,220 7,240 7,260

ClaI
BspDI
PsrI BsmFI
ACACTACCTGTACCCCATTTAGGACCACCCACAGCACCTAACAAAACGGCATCAACCTTCTTGGAGGCTTCCAGCGCCTCATCTGGAAGTGGGACACCTGTAGCATC
TGTGATGGACATGGGGTAAATCCTGGTGGGTGTCGTGGATTGTTTTGCCGTAGTTGGAAGAACCTCCGAAGGTCGCGGAGTAGACCTTCACCCTGTGGACATCGTAG
LEU2
7,280 7,300 7,320 7,340 7,360 7,380

GATAGCAGCACCACCAATTAAATGATTTTCGAAATCGAACTTGACATTGGAACGAACATCAGAAATAGCTTTAAGAACCTTAATGGCTTCGGCTGTGATTTCTTGAC
CTATCGTCGTGGTGGTTAATTTACTAAAAGCTTTAGCTTGAACTGTAACCTTGCTTGTAGTCTTTATCGAAATTCTTGGAATTACCGAAGCCGACACTAAAGAACTG
LEU2
7,400 7,420 7,440 7,460 7,480

BstEII
CAACGTGGTCACCTGGCAAAACGACGATCTTCTTAGGGGCAGACATTACAATGGTATATCCTTGAAATATATATAAAAAAAAAAAAAAAAAAAAAAAAAAAAAAAATG
GTTGCACCAGTGGACCGTTTTGCTGCTAGAAGAATCCCCGTCTGTAATGTTACCATATAGGAACTTTATATATATTTTTTTTTTTTTTTTTTTTTTTTTTTTTTTTTAC
LEU2
7,500 7,520 7,540 7,560 7,580

BseRI PvuI

CAGCTTCTCAATGATATTCGAATACGCTTTGAGGAGATACAGCCTAATATCCGACAAACTGTTTTACAGATTTACGATCGTACTTGTTACCCATCATTGAATTTTGA
GTCGAAGAGTTACTATAAGCTTATGCGAAACTCCTCTATGTCGGATTATAGGCTGTTTGACAAAATGTCTAAATGCTAGCATGAACAATGGGTAGTAACTTAAAACT

7,600 7,620 7,640 7,660 7,680 7,700

PfoI

ACATCCGAACCTGGGAGTTTTCCCTGAAACAGATAGTATATTTGAACCTGTATAATAATATATAGTCTAGCGCTTTACGGAAGACAATGTATGTATTTCGGTTCCTG
TGTAGGCTTGGACCCTCAAAAGGGACTTTGTCTATCATATAAACTTGGACATATTATTATATATCAGATCGCGAAATGCCTTCTGTTACATACATAAAGCCAAGGAC

7,720 7,740 7,760 7,780 7,800

BstAPI BmgBI

GAGAAACTATTGCATCTATTGCATAGGTAATCTTGCACGTCGCATCCCCGGTTCATTTTCTGCGTTTCCATCTTGCACTTCAATAGCATATCTTT
CTCTTTGATAACGTAGATAACGTATCCATTAGAACGTGCAGCGTAGGGGCCAAGTAAAAGACGCAAAGGTAGAACGTGAAGTTATCGTATAGAAA

7,820 7,830 7,840 7,850 7,860 7,870 7,880 7,890 7,900

pKPY514 (7897 bp)

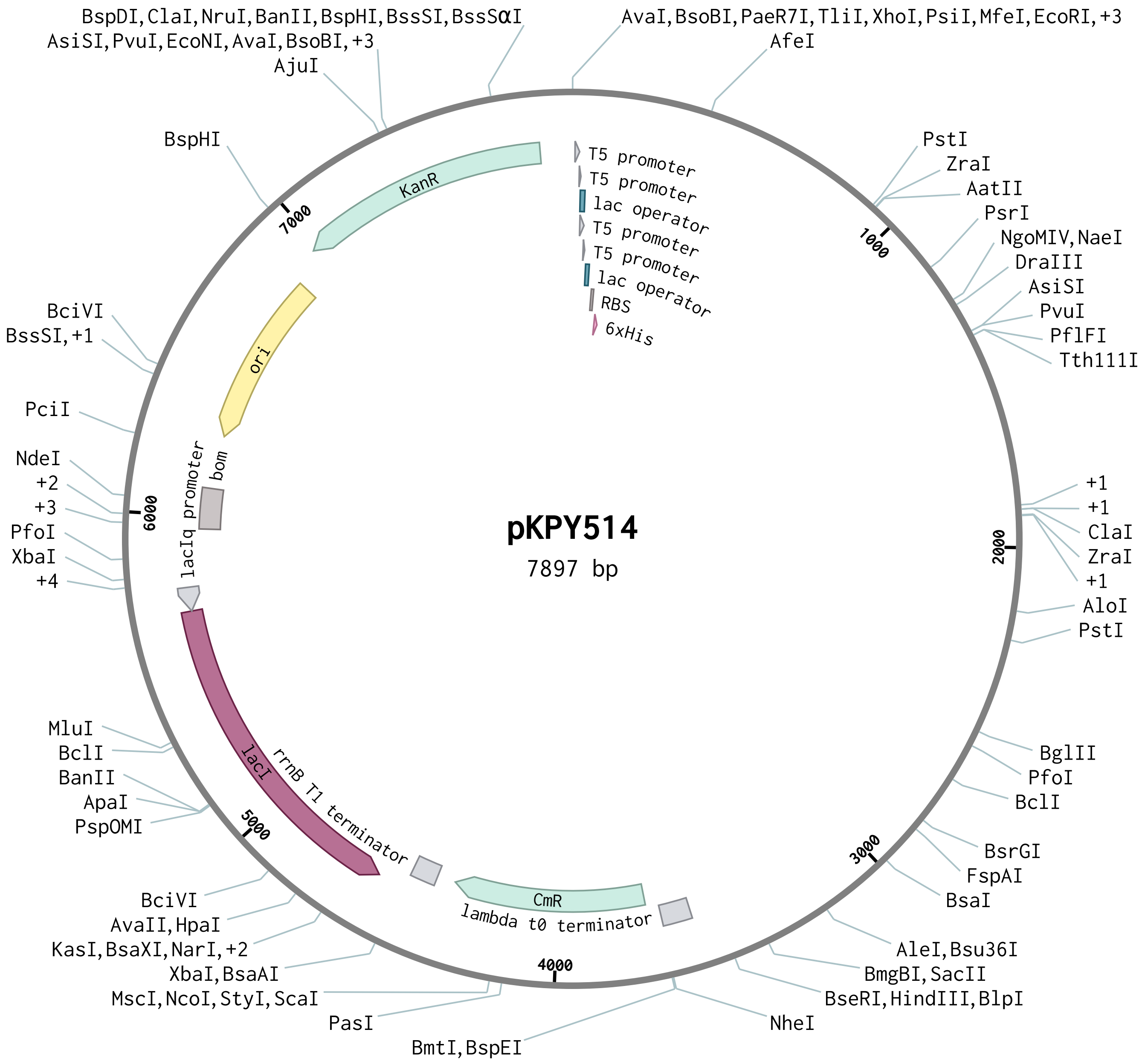

pKPY514 (7897 bp)

PaeR7I
BsoBI
XhoI
TliI
AvaI
PsiI
MfeI
EcoRI

ctcgagaaatcataaaaaatttatttgctttgtgagcggataacaattataatagattcaattgtgagcggataacaatttcacacagaattcattaaagaggagaa
gagctctttagtattttttaaataaacgaaacactcgcctattgttaatattatctaagttaacactcgcctattgttaaagtgtgtcttaagtaatttctcctctt

lac operator
T5 promoter
lac operator
RBS

20 40 60 80 100

BseRI
BamHI
SphI

attaactatgagaggatcgcatcaccatcaccatcacggatccgcatgcatgtcacatctcgcagaactggttgccagtgcgaaggcggccattagccaggcgtcag
taattgatactctcctagcgtagtggtagtggtagtgcctaggcgtacgtacagtgtagagcgtcttgaccaacggtcacgcttccgccggtaatcggtccgcagtc

6xHis

120 140 160 180 200

atgttgccgcgttagataatgtgcgcgtcgaatatttgggtaaaaaagggcacttaacccttcagatgacgaccctgcgtgagctgccgccagaagagcgtccggca
tacaacggcgcaatctattacacgcgcagcttataaacccattttttcccgtgaattgggaagtctactgctgggacgcactcgacggcggtcttctcgcaggccgt

220 240 260 280 300 320

AfeI

gctggtgcggttatcaacgaagcgaaagagcaggttcagcaggcgctgaatgcgcgtaaagcggaactggaaagcgctgcactgaatgcgcgtctggcggcggaaac
cgaccacgccaatagttgcttcgctttctcgtccaagtcgtccgcgacttacgcgcatttcgccttgacctttcgcgacgtgacttacgcgcagaccgccgcctttg

340 360 380 400 420

gattgatgtctctctgccaggtcgtcgcattgaaaacggcggtctgcatccggttacccgtaccatcgaccgtatcgaaagtttcttcggtgagcttggctttaccg
ctaactacagagagacggtccagcagcgtaacttttgccgccagacgtaggccaatgggcatggtagctggcatagctttcaaagaagccactcgaaccgaaatggc

440 460 480 500 520

tggcaaccgggccggaaatcgaagacgattatcataacttcgatgctctgaacattcctggtcaccacccggcgcgcgctgaccacgacactttctggtttgacact
accgttggcccggcctttagcttctgctaatagtattgaagctacgagacttgtaaggaccagtggtgggccgcgcgcgactggtgctgtgaaagaccaaactgtga

540 560 580 600 620 640

acccgcctgctgcgtacccagacctctggcgtacagatccgcaccatgaaagcccagcagccaccgattcgtatcatcgcgcctggccgtgtttatcgtaacgacta
tgggcggacgacgcatgggtctggagaccgcatgtctaggcgtggtactttcgggtcgtcggtggctaagcatagtagcgcggaccggcacaaatagcattgctgat

660 680 700 720 740

cgaccagactcacacgccgatgttccatcagatggaaggtctgattgttgataccaacatcagctttaccaacctgaaaggcacgctgcacgacttcctgcgtaact
gctggtctgagtgtgcggctacaaggtagtctaccttccagactaacaactatggttgtagtcgaaatggttggactttccgtgcgacgtgctgaaggacgcattga

760 780 800 820 840

AatII
PstI ZraI
tctttgaggaagatttgcagattcgcttccgtccttcctacttcccgtttggagaaccttctgcagaagtggacgtcatgggtaaaaacggtaaatggctggaagtg
agaaactccttctaaacgtctaagcgaaggcaggaaggatgaagggcaaacctcttggaagacgtcttcacctgcagtacccatttttgccatttaccgaccttcac
860 880 900 920 940 960

ctgggctgcgggatggtgcatccgaacgtgttgcgtaacgttggcatcgacccggaagtttactctggtttcgccttcgggatggggatggagcgtctgactatgtt
gacccgacgccctaccacgtaggcttgcacaacgcattgcaaccgtagctgggccttcaaatgagaccaaagcggaagccctacccctacctcgcagactgatacaa
980 1,000 1,020 1,040 1,060

PsrI
gcgttacggcgtcaccgacctgcgttcattcttcgaaaacgatctgcgtttcctcaaacagtttaaataaggcaggaatagattatgaaattcagtgaactgtggtt
cgcaatgccgcagtggctggacgcaagtaagaagcttttgctagacgcaaaggagtttgtcaaatttattccgtccttatctaatactttaagtcacttgacaccaa
1,080 1,100 1,120 1,140 1,160

NaeI
NgoMIV
acgcgaatgggtgaacccggcgattgatagcgatgcgctggcaaatcaaatcactatggcgggcctggaagttgacggtgtagaaccggttgccggcagcttccacg
tgcgcttacccacttgggccgctaactatcgctacgcgaccgtttagtttagtgataccgcccggaccttcaactgccacatcttggccaacggccgtcgaaggtgc
1,180 1,200 1,220 1,240 1,260 1,280

AsiSI Tth111I
DraIII PvuI PflFI
gcgtggtcgttggtgaagtggttgagtgtgcgcagcatccgaacgctgacaaactgcgtgtgacaaaagtgaatgtcggcggcgatcgcctgctggacatcgtctgc
cgcaccagcaaccacttcaccaactcacacgcgtcgtaggcttgcgactgtttgacgcacactgttttcacttacagccgccgctagcggacgacctgtagcagacg
1,300 1,320 1,340 1,360 1,380

ggtgcgccaaactgccgtcagggcctgcgtgtagcggtagcgaccattggtgctgttctgccgggtgatttcaaaattaaagcggcgaaactgcgtggcgaaccgtc
ccacgcggtttgacggcagtcccggacgcacatcgccatcgctggtaaccacgacaagacggcccactaaagttttaatttcgccgctttgacgcaccgcttggcag
1,400 1,420 1,440 1,460 1,480

tgaagggatgctgtgctccttctctgaactgggcatttctgacgatcacagcggcattatcgaactgcctgcggatgcgccgattggcaccgatatccgtgaatacc
acttccctacgacacgaggaagagacttgacccgtaaagactgctagtgtcgccgtaatagcttgacggacgcctacgcggctaaccgtggctataggcacttatgg
1,500 1,520 1,540 1,560 1,580 1,600

tgaaacttgatgacaacaccatcgaaatcagcgtgacgccaaaccgtgccgactgcttaggcatcattggtgttgcgcgtgacgttgccgtgctgaaccagctgccg
actttgaactactgttgtggtagctttagtcgcactgcggtttggcacggctgacgaatccgtagtaaccacaacgcgcactgcaacggcacgacttggtcgacggc
1,620 1,640 1,660 1,680 1,700

ctggttcaaccggaaatcgttccggttggtgcgaccatcgacgacacgctgccgattacagtcgaagcgccggaagcctgcccgcgttatcttggccgtgtggtaaa
gaccaagttggcctttagcaaggccaaccacgctggtagctgctgtgcgacggctaatgtcagcttcgcggccttcggacgggcgcaatagaaccggcacaccattt
1,720 1,740 1,760 1,780 1,800

BspDI AatII
BamHI ClaI ZraI
aggcattaacgttaaagcgccaactccgctgtggatgaaagaaaaactgcgtcgttgcgggatccgttctatcgatgcagttgttgacgtcaccaactatgtgctgc
tccgtaattgcaatttcgcggttgaggcgacacctactttctttttgacgcagcaacgccctaggcaagatagctacgtcaacaactgcagtggttgatacacgacg
1,820 1,840 1,860 1,880 1,900 1,920

tcgaactgggccagccgatgcacgctttcgataaagatcgcattgaaggcggcattgtggtgcggatggcgaaagagggcgaaacgctggtgctgctcgacggtact
agcttgacccggtcggctacgtgcgaaagctatttctagcgtaacttccgccgtaacaccacgcctaccgctttctcccgctttgcgaccacgacgagctgccatga
1,940 1,960 1,980 2,000 2,020

gaagcgaagctgaatgctgacactctggtcatcgccgaccacaacaaggcgctggcgatgggcggcatcttcggtggcgaacactctggcgtgaatgacgaaacaca
cttcgcttcgacttacgactgtgagaccagtagcggctggtgttgttccgcgaccgctacccgccgtagaagccaccgcttgtgagaccgcacttactgctttgtgt
2,040 2,060 2,080 2,100 2,120 2,140

AloI
aaacgtgctgctggaatgcgcgttctttagcccgctgtctatcaccggtcgtgctcgtcgtcatggcctgcataccgatgcgtctcaccgttatgagcgtggcgttg
tttgcacgacgaccttacgcgcaagaaatcgggcgacagatagtggccagcacgagcagcagtaccggacgtatggctacgcagagtggcaatactcgcaccgcaac
2,160 2,180 2,200 2,220 2,240

PstI
atccggcactgcagcacaaagcgatggaacgtgcgacccgtctgctgatcgacatctgcggtggtgaggctggcccggtaattgatatcaccaacgaagcaacgctg
taggccgtgacgtcgtgtttcgctaccttgcacgctgggcagacgactagctgtagacgccaccactccgaccgggccattaactatagtggttgcttcgttgcgac
2,260 2,280 2,300 2,320 2,340

ccgaagcgtgcaaccatcactctacgtcgtagcaaactggatcgcctgatcggccatcatattgcggatgagcaggtaactgacattctgcgtcgtctcggctgcga
ggcttcgcacgttggtagtgagatgcagcatcgtttgacctagcggactagccggtagtataacgcctactcgtccattgactgtaagacgcagcagagccgacgct
2,360 2,380 2,400 2,420 2,440 2,460

BglII
agtgaccgaaggcaaagacgaatggcaggcagttgcgccgagctggcgtttcgatatggagattgaagaagatctggttgaagaagtcgcgcgtgtttacggctaca
tcactggcttccgtttctgcttaccgtccgtcaacgcggctcgaccgcaaagctatacctctaacttcttctagaccaacttcttcagcgcgcacaaatgccgatgt
2,480 2,500 2,520 2,540 2,560

PfoI
acaacatcccggatgagccggtacaggcaagcctgattatgggtactcaccgtgaagctgacctgtcgctcaagcgcgtgaaaacgctgctcaacgacaaaggctat
tgttgtagggcctactcggccatgtccgttcggactaatacccatgagtggcacttcgactggacagcgagttcgcgcacttttgcgacgagttgctgtttccgata
2,580 2,600 2,620 2,640 2,660

BclI
caggaagtgatcacctacagcttcgttgatccgaaagtgcagcagatgatccatccaggcgttgaagccttactgctgccaagcccgatctctgttgaaatgtcagc
gtccttcactagtggatgtcgaagcaactaggctttcacgtcgtctactaggtaggtccgcaacttcggaatgacgacggttcgggctagagacaactttacagtcg
2,680 2,700 2,720 2,740 2,760 2,780

BsrGI FspAI

```
aatgcgtctttctctgtggactggcctgctggcaaccgtggtgtacaaccagaaccgtcagcagaaccgtgtgcgcattttcgaaagcggtctgcgtttcgtaccag
ttacgcagaaagagacacctgaccggacgaccgttggcaccacatgttggtcttggcagtcgtcttggcacacgcgtaaaagctttcgccagacgcaaagcatggtc
```

2,800 2,820 2,840 2,860 2,880

BsaI

```
atactcaggcaccgttgggcattcgtcaggatctgatgttagccggtgtgatttgcggtaaccgttacgaagagcactggaacctggcaaaagagaccgttgatttc
tatgagtccgtggcaacccgtaagcagtcctagactacaatcggccacactaaacgccattggcaatgcttctcgtgaccttggaccgttttctctggcaactaaag
```

2,900 2,920 2,940 2,960 2,980

```
tatgatttgaaaggcgatcttgaatccgttctcgacctgaccggtaaactgaatgaggttgagttccgtgcagaagcgaatccggcactgcatccggggcaatccgc
atactaaactttccgctagaacttaggcaagagctggactggccatttgacttactccaactcaaggcacgtcttcgcttaggccgtgacgtaggccccgttaggcg
```

3,000 3,020 3,040 3,060 3,080 3,100

AleI

```
agcgatttatctgaaaggtgaacgtattggttttgttggggttgttcatcctgaactggaacgtaaactggatcttaacggtcgcactctggtgttcgaactggagt
tcgctaaatagactttccacttgcataaccaaaacaaccccaacaagtaggacttgaccttgcatttgacctagaattgccagcgtgagaccacaagcttgacctca
```

3,120 3,140 3,160 3,180 3,200

Bsu36I

```
ggaacaagctcgcagaccgcgtggtgcctcaggcgcgcgagatttctcgcttcccggcgaaccgtcgtgacatcgcggtggtggtcgcagaaaacgttcccgcagcg
ccttgttcgagcgtctggcgcaccacggagtccgcgcgctctaaagagcgaagggccgcttggcagcactgtagcgccaccaccagcgtcttttgcaagggcgtcgc
```

3,220 3,240 3,260 3,280 3,300

BmgBI SacII

```
gatattttatccgaatgtaagaaagttggcgtaaatcaggtagttggcgtaaacttatttgacgtgtaccgcggtaagggtgttgcggaggggtataagagcctcgc
ctataaaataggcttacattctttcaaccgcatttagtccatcaaccgcatttgaataaactgcacatggcgccattcccacaacgcctccccatattctcggagcg
```

3,320 3,340 3,360 3,380 3,400 3,420

BseRI

```
cataagcctgatcctgcaagataccagccgtacactcgaagaagaggagattgccgctaccgtcgccaaatgtgtagaggcattaaaagagcgattccaggcatcat
gtattcggactaggacgttctatggtcggcatgtgagcttcttctcctctaacggcgatggcagcggtttacacatctccgtaattttctcgctaaggtccgtagta
```

3,440 3,460 3,480 3,500 3,520

HindIII BlpI

```
tgagggattgaaagcttaattagctgagcttggactcctgttgatagatccagtaatgacctcagaactccatctggatttgttcagaacgctcggttgccgccggg
actccctaactttcgaattaatcgactcgaacctgaggacaactatctaggtcattactggagtcttgaggtagacctaaacaagtcttgcgagccaacggcggccc
```

lambda t0 terminator

3,540 3,560 3,580 3,600 3,620

BmtI
NheI

cgtttttattggtgagaatccaagctagcttggcgagattttcaggagctaaggaagctaaaatggagaaaaaaatcactggatataccaccgttgatatatccca
gcaaaaaataaccactcttaggttcgatcgaaccgctctaaaagtcctcgattccttcgattttacctctttttttagtgacctatatggtggcaactatatagggt

CmR

3,640 3,660 3,680 3,700 3,720 3,740

atggcatcgtaaagaacattttgaggcatttcagtcagttgctcaatgtacctataaccagaccgttcagctggatattacggcctttttaaagaccgtaaagaaaa
taccgtagcatttcttgtaaaactccgtaaagtcagtcaacgagttacatggatattggtctggcaagtcgacctataatgccggaaaaatttctggcatttctttt

CmR

3,760 3,780 3,800 3,820 3,840

BspEI

ataagcacaagttttatccggcctttattcacattcttgcccgcctgatgaatgctcatccggaatttcgtatggcaatgaaagacggtgagctggtgatatgggat
tattcgtgttcaaaataggccggaaataagtgtaagaacgggcggactacttacgagtaggccttaaagcataccgttactttctgccactcgaccactataccta

CmR

3,860 3,880 3,900 3,920 3,940

agtgttcacccttgttacaccgttttccatgagcaaactgaaacgttttcatcgctctggagtgaataccacgacgatttccggcagtttctacacatatattcgca
tcacaagtgggaacaatgtggcaaaaggtactcgtttgactttgcaaaagtagcgagacctcacttatggtgctgctaaaggccgtcaaagatgtgtatataagcgt

CmR

3,960 3,980 4,000 4,020 4,040 4,060

PasI

agatgtggcgtgttacggtgaaaacctggcctatttccctaaagggtttattgagaatatgtttttcgtctcagccaatccctgggtgagtttcaccagttttgatt
tctacaccgcacaatgccacttttggaccggataaagggatttcccaaataactcttatacaaaaagcagagtcggttagggacccactcaaagtggtcaaaactaa

CmR

4,080 4,100 4,120 4,140 4,160

StyI
MscI NcoI

taaacgtggccaatatggacaacttcttcgcccccgttttcaccatgggcaaatattatacgcaaggcgacaaggtgctgatgccgctggcgattcaggttcatcat
atttgcaccggttatacctgttgaagaagcgggggcaaaagtggtacccgtttataatatgcgttccgctgttccacgactacggcgaccgctaagtccaagtagta

CmR

4,180 4,200 4,220 4,240 4,260 4,280

ScaI

gccgtttgtgatggcttccatgtcggcagaatgcttaatgaattacaacagtactgcgatgagtggcagggcggggcgtaatttttttaaggcagttattggtgccc
cggcaaacactaccgaaggtacagccgtcttacgaattacttaatgttgtcatgacgctactcaccgtcccgccccgcattaaaaaaattccgtcaataaccacggg

CmR

4,300 4,320 4,340 4,360 4,380

ttaaacgcctggggtaatgactctctagcttgaggcatcaaataaaacgaaaggctcagtcgaaagactgggcctttcgttttatctgttgtttgtcggtgaacgct
aatttgcggaccccattactgagagatcgaactccgtagtttattttgctttccgagtcagctttctgacccggaaagcaaaatagacaacaaacagccacttgcga
rrnB T1 terminator
4,400 4,420 4,440 4,460 4,480

XbaI BsaAI
ctcctgagtaggacaaatccgccctctagattacgtgcagtcgatgataagctgtcaaacatgagaattgtgcctaatgagtgagctaacttacattaattgcgttg
gaggactcatcctgtttaggcgggagatctaatgcacgtcagctactattcgacagtttgtactcttaacacggattactcactcgattgaatgtaattaacgcaac
4,500 4,520 4,540 4,560 4,580 4,600

PluTI
SfoI
BsaXI
NarI
KasI
cgctcactgcccgctttccagtcgggaaacctgtcgtgccagctgcattaatgaatcggccaacgcgcggggagaggcggtttgcgtattgggcgccagggtggttt
gcgagtgacgggcgaaaggtcagccctttggacagcacggtcgacgtaattacttagccggttgcgcgcccctctccgccaaacgcataacccgcggtcccaccaaa
lacI
4,620 4,640 4,660 4,680 4,700

AvaII
ttcttttcaccagtgagacgggcaacagctgattgcccttcaccgcctggccctgagagagttgcagcaagcggtccacgctggtttgccccagcaggcgaaaatcc
aagaaaagtggtcactctgcccgttgtcgactaacgggaagtggcggaccgggactctctcaacgtcgttcgccaggtgcgaccaaacggggtcgtccgcttttagg
lacI
4,720 4,740 4,760 4,780 4,800

HpaI BciVI
tgtttgatggtggttaacggcgggatataacatgagctgtcttcggtatcgtcgtatcccactaccgagatatccgcaccaacgcgcagcccggactcggtaatggc
acaaactaccaccaattgccgccctatattgtactcgacagaagccatagcagcatagggtgatggctctataggcgtggttgcgcgtcgggcctgagccattaccg
lacI
4,820 4,840 4,860 4,880 4,900 4,920

gcgcattgcgcccagcgccatctgatcgttggcaaccagcatcgcagtgggaacgatgccctcattcagcatttgcatggtttgttgaaaaccggacatggcactcc
cgcgtaacgcgggtcgcggtagactagcaaccgttggtcgtagcgtcacccttgctacgggagtaagtcgtaaacgtaccaaacaacttttggcctgtaccgtgagg
lacI
4,940 4,960 4,980 5,000 5,020

BanII
ApaI
PspOMI

agtcgccttcccgttccgctatcggctgaatttgattgcgagtgagatatttatgccagccagccagacgcagacgcgccgagacagaacttaatgggcccgctaac
tcagcggaagggcaaggcgatagccgacttaaactaacgctcactctataaatacggtcggtcggtctgcgtctgcgcggctctgtcttgaattacccgggcgattg
lacI
5,040 5,060 5,080 5,100 5,120

agcgcgatttgctggtgacccaatgcgaccagatgctccacgcccagtcgcgtaccgtcttcatgggagaaaataatactgttgatgggtgtctggtcagagacatc
tcgcgctaaacgaccactgggttacgctggtctacgaggtgcgggtcagcgcatggcagaagtaccctcttttattatgacaactacccacagaccagtctctgtag
lacI
5,140 5,160 5,180 5,200 5,220 5,240

BclI MluI

aagaaataacgccggaacattagtgcaggcagcttccacagcaatggcatcctggtcatccagcggatagttaatgatcagcccactgacgcgttgcgcgagaagat
ttctttattgcggccttgtaatcacgtccgtcgaaggtgtcgttaccgtaggaccagtaggtcgcctatcaattactagtcgggtgactgcgcaacgcgctcttcta
lacI
5,260 5,280 5,300 5,320 5,340

tgtgcaccgccgctttacaggcttcgacgccgcttcgttctaccatcgacaccaccacgctggcacccagttgatcggcgcgagatttaatcgccgcgacaatttgc
acacgtggcggcgaaatgtccgaagctgcggcgaagcaagatggtagctgtggtggtgcgaccgtgggtcaactagccgcgctctaaattagcggcgctgttaaacg
lacI
5,360 5,380 5,400 5,420 5,440

gacggcgcgtgcagggccagactggaggtggcaacgccaatcagcaacgactgtttgcccgccagttgttgtgccacgcggttgggaatgtaattcagctccgccat
ctgccgcgcacgtcccggtctgacctccaccgttgcggttagtcgttgctgacaaacgggcggtcaacaacacggtgcgccaacccttacattaagtcgaggcggta
lacI
5,460 5,480 5,500 5,520 5,540 5,560

cgccgcttccactttttcccgcgttttcgcagaaacgtggctggcctggttcaccacgcgggaaacggtctgataagagacaccggcatactctgcgacatcgtata
gcggcgaaggtgaaaaagggcgcaaaagcgtctttgcaccgaccggaccaagtggtgcgccctttgccagactattctctgtggccgtatgagacgctgtagcatat
lacI
5,580 5,600 5,620 5,640 5,660

acgttactggtttcacattcaccaccctgaattgactctcttccgggcgctatcatgccataccgcgaaaggttttgcaccattcgatggtgtcggaatttcgggca
tgcaatgaccaaagtgtaagtggtgggacttaactgagagaaggcccgcgatagtacggtatggcgctttccaaaacgtggtaagctaccacagccttaaagcccgt
lacI lacIq promoter
5,680 5,700 5,720 5,740 5,760

PpuMI AvaII MscI FspAI XbaI PfoI

gcgttgggtcctggccacgggtgcgcatgatctagagctgcctcgcgcgtttcggtgatgacggtgaaaacctctgacacatgcagctcccggagacggtcacagct
cgcaacccaggaccggtgcccacgcgtactagatctcgacggagcgcgcaaagccactactgccacttttggagactgtgtacgtcgagggcctctgccagtgtcga

5,780 5,800 5,820 5,840 5,860 5,880

Tth111I PflFI BsaAI

tgtctgtaagcggatgccgggagcagacaagcccgtcagggcgcgtcagcgggtgttggcgggtgtcggggcgcagccatgacccagtcacgtagcgatagcggagt
acagacattcgcctacggccctcgtctgttcgggcagtcccgcgcagtcgcccacaaccgcccacagccccgcgtcggtactgggtcagtgcatcgctatcgcctca

bom

5,900 5,920 5,940 5,960 5,980

BstZ17I AccI NdeI

gtatactggcttaactatgcggcatcagagcagattgtactgagagtgcaccatatgcggtgtgaaataccgcacagatgcgtaaggagaaaataccgcatcaggcg
catatgaccgaattgatacgccgtagtctcgtctaacatgactctcacgtggtatacgccacactttatggcgtgtctacgcattcctcttttatggcgtagtccgc

bom

6,000 6,020 6,040 6,060 6,080

ctcttccgcttcctcgctcactgactcgctgcgctcggtcgttcggctgcggcgagcggtatcagctcactcaaaggcggtaatacggttatccacagaatcagggg
gagaaggcgaaggagcgagtgactgagcgacgcgagccagcaagccgacgccgctcgccatagtcgagtgagtttccgccattatgccaataggtgtcttagtcccc

6,100 6,120 6,140 6,160 6,180 6,200

PciI

ataacgcaggaaagaacatgtgagcaaaaggccagcaaaaggccaggaaccgtaaaaaggccgcgttgctggcgtttttccataggctccgcccccctgacgagcat
tattgcgtcctttcttgtacactcgttttccggtcgttttccggtccttggcatttttccggcgcaacgaccgcaaaaaggtatccgaggcggggggactgctcgta

ori

6,220 6,240 6,260 6,280 6,300

BssSαI BssSI

cacaaaaatcgacgctcaagtcagaggtggcgaaacccgacaggactataaagataccaggcgtttccccctggaagctccctcgtgcgctctcctgttccgaccct
gtgtttttagctgcgagttcagtctccaccgctttgggctgtcctgatatttctatggtccgcaaagggggaccttcgagggagcacgcgagaggacaaggctggga

ori

6,320 6,340 6,360 6,380 6,400 6,420

BciVI

gccgcttaccggatacctgtccgcctttctcccttcgggaagcgtggcgctttctcatagctcacgctgtaggtatctcagttcggtgtaggtcgttcgctccaagc
cggcgaatggcctatggacaggcggaaagagggaagcccttcgcaccgcgaaagagtatcgagtgcgacatccatagagtcaagccacatccagcaagcgaggttcg

ori

6,440 6,460 6,480 6,500 6,520

tgggctgtgtgcacgaaccccccgttcagcccgaccgctgcgccttatccggtaactatcgtcttgagtccaacccggtaagacacgacttatcgccactggcagca
acccgacacacgtgcttggggggcaagtcgggctggcgacgcggaataggccattgatagcagaactcaggttgggccattctgtgctgaatagcggtgaccgtcgt
ori
6,540 6,560 6,580 6,600 6,620

gccactggtaacaggattagcagagcgaggtatgtaggcggtgctacagagttcttgaagtggtggcctaactacggctacactagaaggacagtatttggtatctg
cggtgaccattgtcctaatcgtctcgctccatacatccgccacgatgtctcaagaacttcaccaccggattgatgccgatgtgatcttcctgtcataaaccatagac
ori
6,640 6,660 6,680 6,700 6,720 6,740

cgctctgctgaagccagttaccttcggaaaaagagttggtagctcttgatccggcaaacaaaccaccgctggtagcggtggtttttttgtttgcaagcagcagatta
gcgagacgacttcggtcaatggaagcctttttctcaaccatcgagaactaggccgtttgtttggtggcgaccatcgccaccaaaaaaacaaacgttcgtcgtctaat
ori
6,760 6,780 6,800 6,820 6,840

cgcgcagaaaaaaaggatctcaagaagatcctttgatcttttctacggggtctgacgctcagtggaacgaaaactcacgttaagggattttggtcatggcgttaagg
gcgcgtctttttttcctagagttcttctaggaaactagaaaagatgccccagactgcgagtcaccttgcttttgagtgcaattccctaaaaccagtaccgcaattcc
ori
6,860 6,880 6,900 6,920 6,940

BspHI
gattttggtcatgaattaattcttagaaaaactcatcgagcatcaaatgaaactgcaatttattcatatcaggattatcaataccatatttttgaaaaagccgtttc
ctaaaaccagtacttaattaagaatctttttgagtagctcgtagtttactttgacgttaaataagtatagtcctaatagttatggtataaaaactttttcggcaaag
KanR
6,960 6,980 7,000 7,020 7,040 7,060

tgtaatgaaggagaaaactcaccgaggcagttccataggatggcaagatcctggtatcggtctgcgattccgactcgtccaacatcaatacaacctattaatttccc
acattacttcctcttttgagtggctccgtcaaggtatcctaccgttctaggaccatagccagacgctaaggctgagcaggttgtagttatgttggataattaaaggg
KanR
7,080 7,100 7,120 7,140 7,160

ctcgtcaaaaataaggttatcaagtgagaaatcaccatgagtgacgactgaatccggtgagaatggcaaaagtttatgcatttctttccagacttgttcaacaggcc
gagcagtttttattccaatagttcactctttagtggtactcactgctgacttaggccactcttaccgttttcaaatacgtaaagaaaggtctgaacaagttgtccgg
KanR
7,180 7,200 7,220 7,240 7,260

AsiSI
AjuI PvuI
agccattacgctcgtcatcaaaatcactcgcatcaaccaaaccgttattcattcgtgattgcgcctgagcgagacgaaatacgcgatcgctgttaaaaggacaatta
tcggtaatgcgagcagtagttttagtgagcgtagttggtttggcaataagtaagcactaacgcggactcgctctgctttatgcgctagcgacaattttcctgttaat
KanR
7,280 7,300 7,320 7,340 7,360 7,380

XmaI
SmaI
AvaI
TspMI
EcoNI BsoBI

caaacaggaatcgaatgcaaccggcgcaggaacactgccagcgcatcaacaatattttcacctgaatcaggatattcttctaatacctggaatgctgttttcccggg
gtttgtccttagcttacgttggccgcgtccttgtgacggtcgcgtagttgttataaaagtggacttagtcctataagaagattatggaccttacgacaaaagggccc

KanR

7,400 7,420 7,440 7,460 7,480

gatcgcagtggtgagtaaccatgcatcatcaggagtacggataaaatgcttgatggtcggaagaggcataaattccgtcagccagtttagtctgaccatctcatctg
ctagcgtcaccactcattggtacgtagtagtcctcatgcctattttacgaactaccagccttctccgtatttaaggcagtcggtcaaatcagactggtagagtagac

KanR

7,500 7,520 7,540 7,560 7,580

BspDI
ClaI

taacatcattggcaacgctacctttgccatgtttcagaaacaactctggcgcatcgggcttcccatacaatcgatagattgtcgcacctgattgcccgacattatcg
attgtagtaaccgttgcgatggaaacggtacaaagtctttgttgagaccgcgtagcccgaagggtatgttagctatctaacagcgtggactaacgggctgtaatagc

KanR

7,600 7,620 7,640 7,660 7,680 7,700

NruI BanII

cgagcccatttatacccatataaatcagcatccatgttggaatttaatcgcggcctagagcaagacgtttcccgttgaatatggctcataacaccccttgtattact
gctcgggtaaatatgggtatatttagtcgtaggtacaaccttaaattagcgccggatctcgttctgcaaagggcaacttataccgagtattgtggggaacataatga

KanR

7,720 7,740 7,760 7,780 7,800

BssSαI
BspHI BssSI

gtttatgtaagcagacagttttattgttcatgaccatgacattaacctataaaaataggcgtatcacgaggccctttcgtcttcac
caaatacattcgtctgtcaaaataacaagtactggtactgtaattggatatttttatccgcatagtgctccgggaaagcagaagtg

7,820 7,830 7,840 7,850 7,860 7,870 7,880 7,890

pKPY-Ex4 (4648 bp)

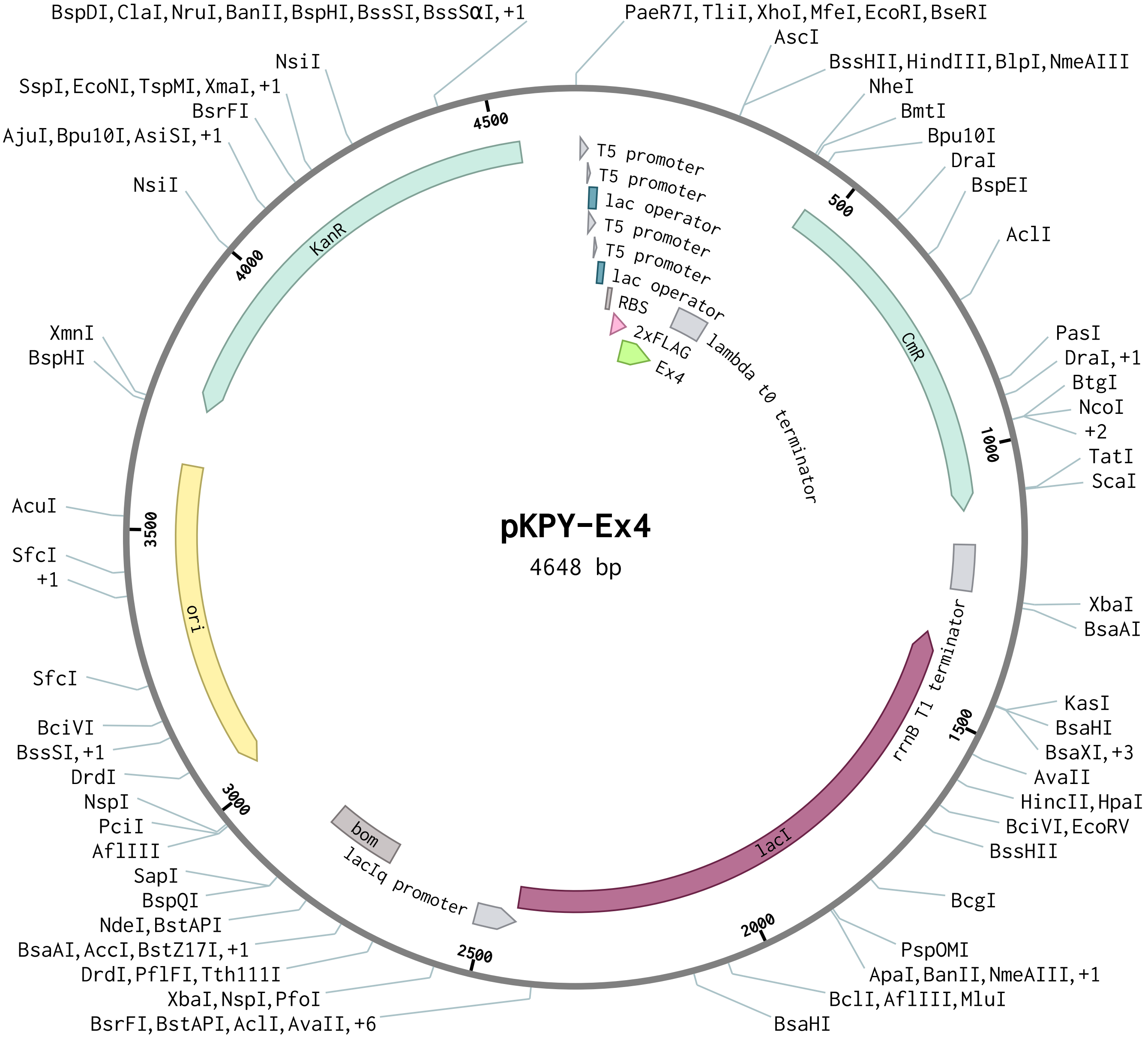

pKPY-Ex4 (4648 bp)

PaeR7I XhoI TliI MfeI EcoRI

```
ctcgagaaatcataaaaaatttatttgctttgtgagcggataacaattataatagattcaattgtgagcggataacaatttcacacagaattcattaaagaggagaa
gagctctttagtattttttaaataaacgaaacactcgcctattgttaatattatctaagttaacactcgcctattgttaaagtgtgtcttaagtaatttctcctctt
```

lac operator, T5 promoter, lac operator, RBS

20 40 60 80 100

BseRI

```
attaactatgagaggatcgGATTATAAAGATGATGATGATAAAGATTATAAAGATGATGATGATAAACATGGCGAAGGCACCTTTACCAGCGATCTGAGCAAACAGA
taattgatactctcctagcCTAATATTTCTACTACTACTATTTCTAATATTTCTACTACTACTATTTGTACCGCTTCCGTGGAAATGGTCGCTAGACTCGTTTGTCT
```

2xFLAG, Ex4

120 140 160 180 200

BssHII AscI HindIII BlpI NmeAIII

```
TGGAAGAAGAAGCGGTGCGCCTGTTTATTGAATGGCTGAAAAACGGCGGCCCGAGCAGCGGCGCGCCGCCGCCGAGCTAAagcttaattagctgagcttggactcct
ACCTTCTTCTTCGCCACGCGGACAAATAACTTACCGACTTTTTGCCGCCGGGCTCGTCGCCGCGCGGCGGCGGCTCGATTtcgaattaatcgactcgaacctgagga
```

Ex4

220 240 260 280 300 320

BmtI NheI

```
gttgatagatccagtaatgacctcagaactccatctggatttgttcagaacgctcggttgccgccgggcgtttttttattggtgagaatccaagctagcttggcgaga
caactatctaggtcattactggagtcttgaggtagacctaaacaagtcttgcgagccaacggcggcccgcaaaaaaataaccactcttaggttcgatcgaaccgctct
```

lambda t0 terminator

340 360 380 400 420

Bpu10I

```
ttttcaggagctaaggaagctaaaatggagaaaaaaatcactggatataccaccgttgatatatcccaatggcatcgtaaagaacattttgaggcatttcagtcagt
aaaagtcctcgattccttcgattttacctctttttttagtgacctatatggtggcaactatatagggttaccgtagcatttcttgtaaaactccgtaaagtcagtca
```

CmR

440 460 480 500 520

DraI

```
tgctcaatgtacctataaccagaccgttcagctggatattacggcctttttaaagaccgtaaagaaaaataagcacaagttttatccggcctttattcacattcttg
acgagttacatggatattggtctggcaagtcgacctataatgccggaaaaatttctggcatttctttttattcgtgttcaaaataggccggaaataagtgtaagaac
```

CmR

540 560 580 600 620 640

BspEI

cccgcctgatgaatgctcatccggaatttcgtatggcaatgaaagacggtgagctggtgatatgggatagtgttcacccttgttacaccgttttccatgagcaaact
gggcggactacttacgagtaggccttaaagcataccgttactttctgccactcgaccactatacccctatcacaagtgggaacaatgtggcaaaaggtactcgtttga

CmR

660 680 700 720 740

AclI

gaaacgttttcatcgctctggagtgaataccacgacgatttccggcagtttctacacatatattcgcaagatgtggcgtgttacggtgaaaacctggcctatttccc
ctttgcaaaagtagcgagacctcacttatggtgctgctaaaggccgtcaaagatgtgtatataagcgttctacaccgcacaatgccacttttggaccggataaaggg

CmR

760 780 800 820 840

PasI DraI MscI

taaagggtttattgagaatatgtttttcgtctcagccaatccctgggtgagtttcaccagttttgatttaaacgtggccaatatggacaacttcttcgcccccgttt
atttcccaaataactcttatacaaaaagcagagtcggttagggacccactcaaagtggtcaaaactaaatttgcaccggttatacctgttgaagaagcgggggcaaa

CmR

860 880 900 920 940 960

StyI
NcoI
BtgI SspI

tcaccatgggcaaatattatacgcaaggcgacaaggtgctgatgccgctggcgattcaggttcatcatgccgtttgtgatggcttccatgtcggcagaatgcttaat
agtggtacccgtttataatatgcgttccgctgttccacgactacggcgaccgctaagtccaagtagtacggcaaacactaccgaaggtacagccgtcttacgaatta

CmR

980 1,000 1,020 1,040 1,060

ScaI
TatI

gaattacaacagtactgcgatgagtggcagggcggggcgtaatttttttaaggcagttattggtgcccttaaacgcctggggtaatgactctctagcttgaggcatc
cttaatgttgtcatgacgctactcaccgtcccgccccgcattaaaaaaattccgtcaataaccacgggaatttgcggaccccattactgagagatcgaactccgtag

CmR

1,080 1,100 1,120 1,140 1,160

XbaI BsaAI

aaataaaacgaaaggctcagtcgaaagactgggcctttcgttttatctgttgtttgtcggtgaacgctctcctgagtaggacaaatccgccctctagattacgtgca
tttattttgctttccgagtcagctttctgacccggaaagcaaaatagacaacaaacagccacttgcgagaggactcatcctgtttaggcgggagatctaatgcacgt

rrnB T1 terminator

1,180 1,200 1,220 1,240 1,260 1,280

```
gtcgatgataagctgtcaaacatgagaattgtgcctaatgagtgagctaacttacattaattgcgttgcgctcactgcccgctttccagtcgggaaacctgtcgtgc
cagctactattcgacagtttgtactcttaacacggattactcactcgattgaatgtaattaacgcaacgcgagtgacgggcgaaaggtcagccctttggacagcacg
```

lacI

1,300 1,320 1,340 1,360 1,380

PluTI SfoI BsaXI BsaHI NarI KasI

```
cagctgcattaatgaatcggccaacgcgcggggagaggcggtttgcgtattgggcgccagggtggtttttcttttcaccagtgagacgggcaacagctgattgccct
gtcgacgtaattacttagccggttgcgcgcccctctccgccaaacgcataacccgcggtcccaccaaaaagaaaagtggtcactctgcccgttgtcgactaacggga
```

lacI

1,400 1,420 1,440 1,460 1,480

AvaII HincII HpaI

```
tcaccgcctggccctgagagagttgcagcaagcggtccacgctggtttgccccagcaggcgaaaatcctgtttgatggtggttaacggcgggatataacatgagctg
agtggcggaccgggactctctcaacgtcgttcgccaggtgcgaccaaacggggtcgtccgcttttaggacaaactaccaccaattgccgccctatattgtactcgac
```

lacI

1,500 1,520 1,540 1,560 1,580 1,600

EcoRV BciVI BssHII

```
tcttcggtatcgtcgtatcccactaccgagatatccgcaccaacgcgcagcccggactcggtaatggcgcgcattgcgcccagcgccatctgatcgttggcaaccag
agaagccatagcagcatagggtgatggctctataggcgtggttgcgcgtcgggcctgagccattaccgcgcgtaacgcgggtcgcggtagactagcaaccgttggtc
```

lacI

1,620 1,640 1,660 1,680 1,700

BcgI

```
catcgcagtgggaacgatgccctcattcagcatttgcatggtttgttgaaaaccggacatggcactccagtcgccttcccgttccgctatcggctgaatttgattgc
gtagcgtcacccttgctacgggagtaagtcgtaaacgtaccaaacaacttttggcctgtaccgtgaggtcagcggaagggcaaggcgatagccgacttaaactaacg
```

lacI

1,720 1,740 1,760 1,780 1,800

BanII ApaI PspOMI NmeAIII BstEII

```
gagtgagatatttatgccagccagccagacgcagacgcgccgagacagaacttaatgggcccgctaacagcgcgatttgctggtgacccaatgcgaccagatgctcc
ctcactctataaatacggtcggtcggtctgcgtctgcgcggctctgtcttgaattacccgggcgattgtcgcgctaaacgaccactgggttacgctggtctacgagg
```

lacI

1,820 1,840 1,860 1,880 1,900 1,920

acgcccagtcgcgtaccgtcttcatgggagaaaataatactgttgatgggtgtctggtcagagacatcaagaaataacgccggaacattagtgcaggcagcttccac
tgcgggtcagcgcatggcagaagtaccctcttttattatgacaactacccacagaccagtctctgtagttctttattgcggccttgtaatcacgtccgtcgaaggtg
lacI
1,940 1,960 1,980 2,000 2,020

AflIII
BclI MluI BsaHI
agcaatggcatcctggtcatccagcggatagttaatgatcagcccactgacgcgttgcgcgagaagattgtgcaccgccgctttacaggcttcgacgccgcttcgtt
tcgttaccgtaggaccagtaggtcgcctatcaattactagtcgggtgactgcgcaacgcgctcttctaacacgtggcggcgaaatgtccgaagctgcggcgaagcaa
lacI
2,040 2,060 2,080 2,100 2,120 2,140

ctaccatcgacaccaccacgctggcacccagttgatcggcgcgagatttaatcgccgcgacaatttgcgacggcgcgtgcagggccagactggaggtggcaacgcca
gatggtagctgtggtggtgcgaccgtgggtcaactagccgcgctctaaattagcggcgctgttaaacgctgccgcgcacgtcccggtctgacctccaccgttgcggt
lacI
2,160 2,180 2,200 2,220 2,240

atcagcaacgactgtttgcccgccagttgttgtgccacgcggttgggaatgtaattcagctccgccatcgccgcttccactttttcccgcgttttcgcagaaacgtg
tagtcgttgctgacaaacgggcggtcaacaacacggtgcgccaacccttacattaagtcgaggcggtagcggcgaaggtgaaaaagggcgcaaaagcgtctttgcac
lacI
2,260 2,280 2,300 2,320 2,340

BsrFI BstAPI AclI
gctggcctggttcaccacgcgggaaacggtctgataagagacaccggcatactctgcgacatcgtataacgttactggtttcacattcaccaccctgaattgactct
cgaccggaccaagtggtgcgccctttgccagactattctctgtggccgtatgagacgctgtagcatattgcaatgaccaaagtgtaagtggtgggacttaactgaga
lacI lacIq promoter
2,360 2,380 2,400 2,420 2,440 2,460

EcoO109I
PpuMI BtgI FspAI
AvaII MscI FspI XbaI
cttccgggcgctatcatgccataccgcgaaaggttttgcaccattcgatggtgtcggaatttcgggcagcgttgggtcctggccacgggtgcgcatgatctagagct
gaaggcccgcgatagtacggtatggcgctttccaaaacgtggtaagctaccacagccttaaagcccgtcgcaacccaggaccggtgcccacgcgtactagatctcga
lacIq promoter
2,480 2,500 2,520 2,540 2,560

PfoI
NspI DrdI
gcctcgcgcgtttcggtgatgacggtgaaaacctctgacacatgcagctcccggagacggtcacagcttgtctgtaagcggatgccgggagcagacaagcccgtcag
cggagcgcgcaaagccactactgccacttttggagactgtgtacgtcgagggcctctgccagtgtcgaacagacattcgcctacggccctcgtctgttcgggcagtc
2,580 2,600 2,620 2,640 2,660

Tth111I
PflFI BsaAI
BstZ17I
AccI
TatI

ggcgcgtcagcgggtgttggcgggtgtcggggcgcagccatgacccagtcacgtagcgatagcggagtgtatactggcttaactatgcggcatcagagcagattgta
ccgcgcagtcgcccacaaccgcccacagccccgcgtcggtactgggtcagtgcatcgctatcgcctcacatatgaccgaattgatacgccgtagtctcgtctaacat

bom

2,680 2,700 2,720 2,740 2,760 2,780

BstAPI
NdeI
BspQI
SapI

ctgagagtgcaccatatgcggtgtgaaataccgcacagatgcgtaaggagaaaataccgcatcaggcgctcttccgcttcctcgctcactgactcgctgcgctcggt
gactctcacgtggtatacgccacactttatggcgtgtctacgcattcctcttttatggcgtagtccgcgagaaggcgaaggagcgagtgactgagcgacgcgagcca

bom

2,800 2,820 2,840 2,860 2,880

NspI
AflIII
PciI

cgttcggctgcggcgagcggtatcagctcactcaaaggcggtaatacggttatccacagaatcaggggataacgcaggaaagaacatgtgagcaaaaggccagcaaa
gcaagccgacgccgctcgccatagtcgagtgagtttccgccattatgccaataggtgtcttagtcccctattgcgtcctttcttgtacactcgttttccggtcgttt

2,900 2,920 2,940 2,960 2,980

DrdI

aggccaggaaccgtaaaaaggccgcgttgctggcgtttttccataggctccgcccccctgacgagcatcacaaaaatcgacgctcaagtcagaggtggcgaaacccg
tccggtccttggcatttttccggcgcaacgaccgcaaaaaggtatccgaggcggggggactgctcgtagtgtttttagctgcgagttcagtctccaccgctttgggc

ori

3,000 3,020 3,040 3,060 3,080 3,100

BssSαI
BssSI
BciVI

acaggactataaagataccaggcgtttccccctggaagctccctcgtgcgctctcctgttccgaccctgccgcttaccggatacctgtccgcctttctcccttcggg
tgtcctgatatttctatggtccgcaaagggggaccttcgagggagcacgcgagaggacaaggctgggacggcgaatggcctatggacaggcggaaagagggaagccc

ori

3,120 3,140 3,160 3,180 3,200

SfcI

aagcgtggcgctttctcatagctcacgctgtaggtatctcagttcggtgtaggtcgttcgctccaagctgggctgtgtgcacgaaccccccgttcagcccgaccgct
ttcgcaccgcgaaagagtatcgagtgcgacatccatagagtcaagccacatccagcaagcgaggttcgacccgacacacgtgcttggggggcaagtcgggctggcga

ori

3,220 3,240 3,260 3,280 3,300

AlwNI

gcgccttatccggtaactatcgtcttgagtccaacccggtaagacacgacttatcgccactggcagcagccactggtaacaggattagcagagcgaggtatgtaggc
cgcggaataggccattgatagcagaactcaggttgggccattctgtgctgaatagcggtgaccgtcgtcggtgaccattgtcctaatcgtctcgctccatacatccg

ori

3,320 3,340 3,360 3,380 3,400 3,420

SfcI AcuI

ggtgctacagagttcttgaagtggtggcctaactacggctacactagaaggacagtatttggtatctgcgctctgctgaagccagttaccttcggaaaaagagttgg
ccacgatgtctcaagaacttcaccaccggattgatgccgatgtgatcttcctgtcataaaccatagacgcgagacgacttcggtcaatggaagcctttttctcaacc

ori

3,440 3,460 3,480 3,500 3,520

tagctcttgatccggcaaacaaaccaccgctggtagcggtggtttttttgtttgcaagcagcagattacgcgcagaaaaaaaggatctcaagaagatcctttgatct
atcgagaactaggccgtttgtttggtggcgaccatcgccaccaaaaaaacaaacgttcgtcgtctaatgcgcgtcttttttttcctagagttcttctaggaaactaga

ori

3,540 3,560 3,580 3,600 3,620

BspHI XmnI

tttctacggggtctgacgctcagtggaacgaaaactcacgttaagggattttggtcatggcgttaagggattttggtcatgaattaattcttagaaaaactcatcga
aaagatgccccagactgcgagtcaccttgcttttgagtgcaattccctaaaaccagtaccgcaattccctaaaaccagtacttaattaagaatctttttgagtagct

KanR

3,640 3,660 3,680 3,700 3,720 3,740

gcatcaaatgaaactgcaatttattcatatcaggattatcaataccatatttttgaaaaagccgtttctgtaatgaaggagaaaactcaccgaggcagttccatagg
cgtagtttactttgacgttaaataagtatagtcctaatagttatggtataaaaactttttcggcaaagacattacttcctcttttgagtggctccgtcaaggtatcc

KanR

3,760 3,780 3,800 3,820 3,840

atggcaagatcctggtatcggtctgcgattccgactcgtccaacatcaatacaacctattaatttcccctcgtcaaaaataaggttatcaagtgagaaatcaccatg
taccgttctaggaccatagccagacgctaaggctgagcaggttgtagttatgttggataattaaaggggagcagtttttattccaatagttcactctttagtggtac

KanR

3,860 3,880 3,900 3,920 3,940

NsiI

agtgacgactgaatccggtgagaatggcaaaagtttatgcatttctttccagacttgttcaacaggccagccattacgctcgtcatcaaaatcactcgcatcaacca
tcactgctgacttaggccactcttaccgttttcaaatacgtaaagaaaggtctgaacaagttgtccggtcggtaatgcgagcagtagttttagtgagcgtagttggt

KanR

3,960 3,980 4,000 4,020 4,040 4,060

Bpu10I AsiSI
AjuI PvuI BsrFI

```
aaccgttattcattcgtgattgcgcctgagcgagacgaaatacgcgatcgctgttaaaaggacaattacaaacaggaatcgaatgcaaccggcgcaggaacactgcc
ttggcaataagtaagcactaacgcggactcgctctgctttatgcgctagcgacaattttcctgttaatgtttgtccttagcttacgttggccgcgtccttgtgacgg
```

KanR

4,080 4,100 4,120 4,140 4,160

SmaI
TspMI
SspI EcoNI XmaI NsiI

```
agcgcatcaacaatattttcacctgaatcaggatattcttctaatacctggaatgctgttttcccggggatcgcagtggtgagtaaccatgcatcatcaggagtacg
tcgcgtagttgttataaaagtggacttagtcctataagaagattatggaccttacgacaaaagggcccctagcgtcaccactcattggtacgtagtagtcctcatgc
```

KanR

4,180 4,200 4,220 4,240 4,260 4,280

```
gataaaatgcttgatggtcggaagaggcataaattccgtcagccagtttagtctgaccatctcatctgtaacatcattggcaacgctacctttgccatgtttcagaa
ctattttacgaactaccagccttctccgtatttaaggcagtcggtcaaatcagactggtagagtagacattgtagtaaccgttgcgatggaaacggtacaaagtctt
```

KanR

4,300 4,320 4,340 4,360 4,380

BspDI
ClaI NruI BanII

```
acaactctggcgcatcgggcttcccatacaatcgatagattgtcgcacctgattgcccgacattatcgcgagcccatttatacccatataaatcagcatccatgttg
tgttgagaccgcgtagcccgaagggtatgttagctatctaacagcgtggactaacgggctgtaatagcgctcgggtaaatatgggtatatttagtcgtaggtacaac
```

KanR

4,400 4,420 4,440 4,460 4,480

BspHI

```
gaatttaatcgcggcctagagcaagacgtttcccgttgaatatggctcataacaccccttgtattactgtttatgtaagcagacagttttattgttcatgaccatga
cttaaattagcgccggatctcgttctgcaaagggcaacttataccgagtattgtggggaacataatgacaaatacattcgtctgtcaaaataacaagtactggtact
```

KanR

4,500 4,520 4,540 4,560 4,580 4,600

EcoO109I
BssSαI
BssSI

```
cattaacctataaaaataggcgtatcacgaggccctttcgtcttcac
gtaattggatatttttatccgcatagtgctccgggaaagcagaagtg
```

4,610 4,620 4,630 4,640

pKPY-FS-Ex4 (partial sequence) (415 bp)

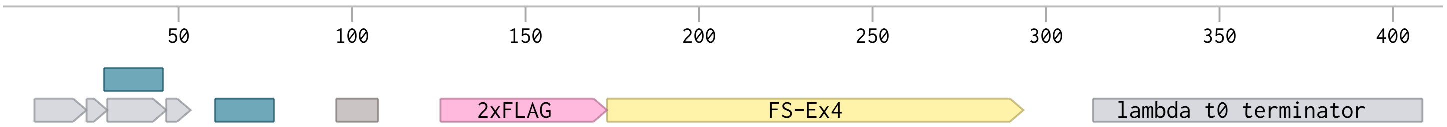

pKPY-FS-Ex4 (partial sequence) (415 bp)

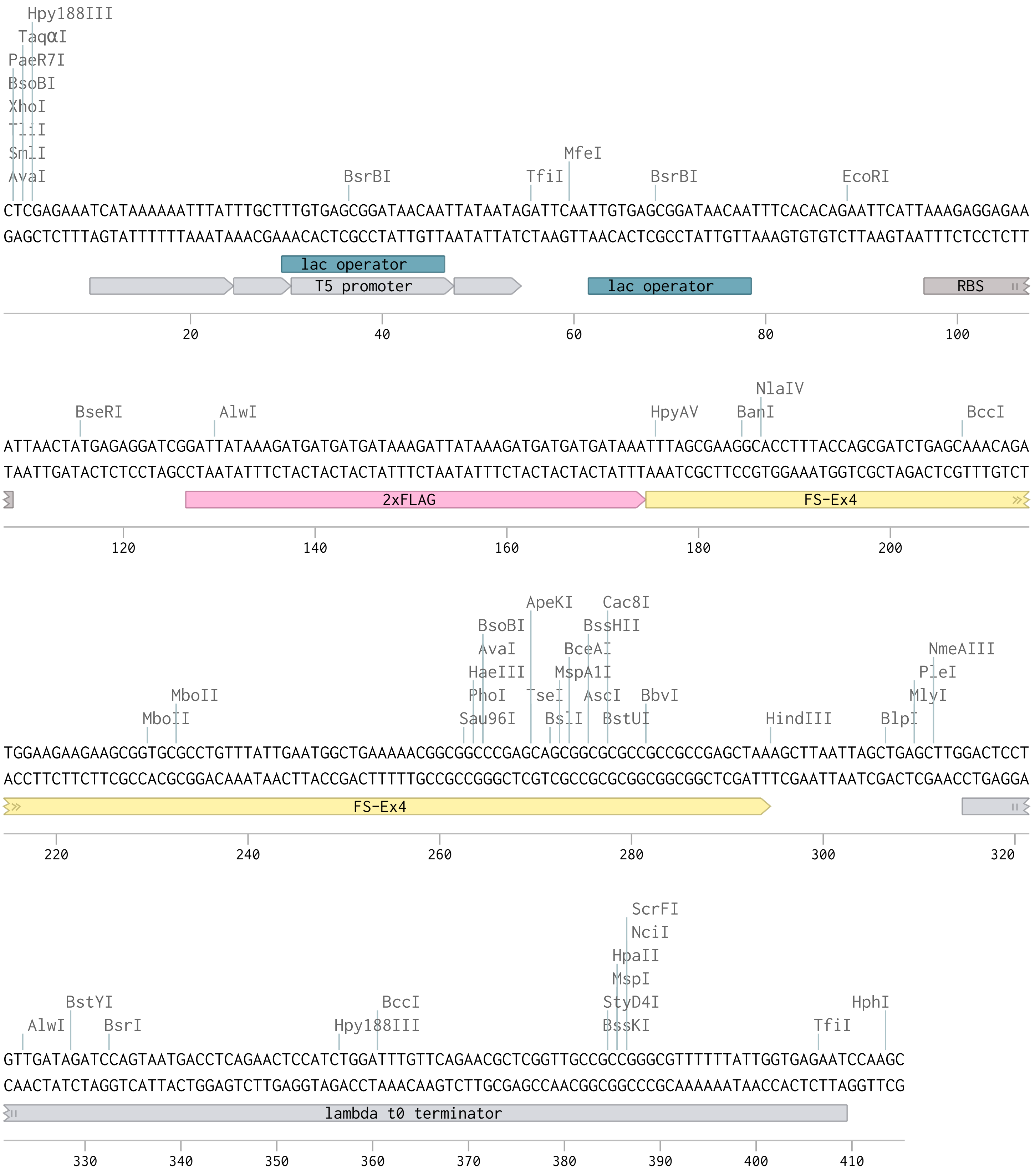

pKPY-HA-Ex4 (partial sequence) (415 bp)

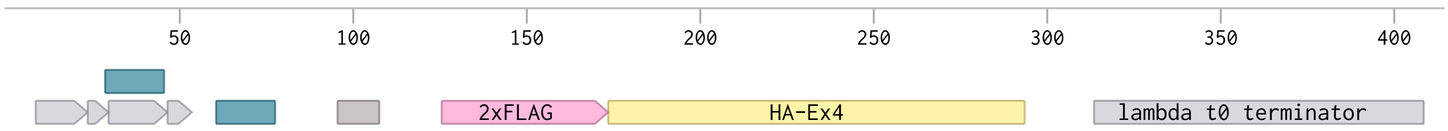

2019/2/18 14:41:33

pKPY-HA-Ex4 (partial sequence) (415 bp)

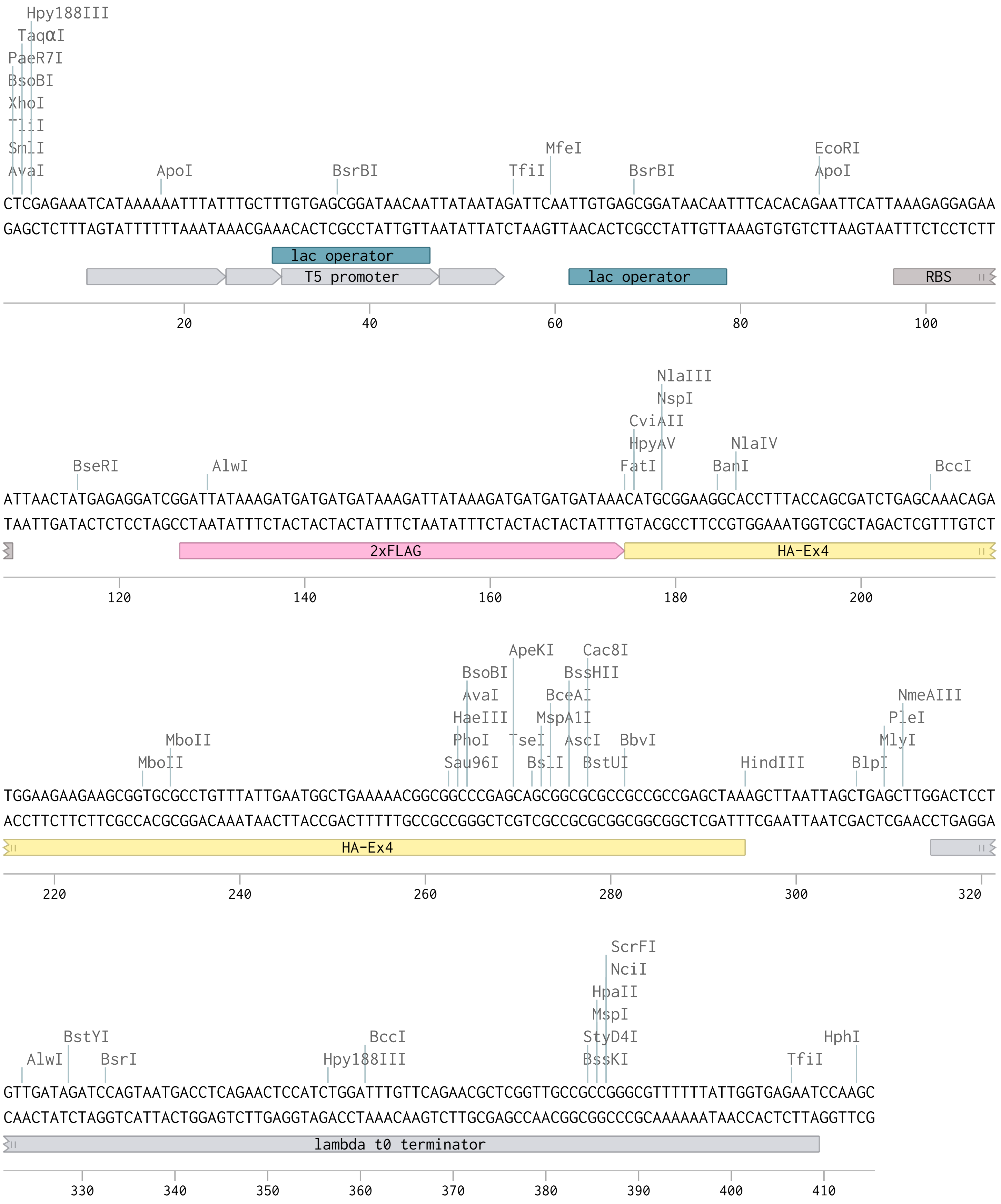

pKPY-AL-Ex4 (partial sequence) (415 bp)

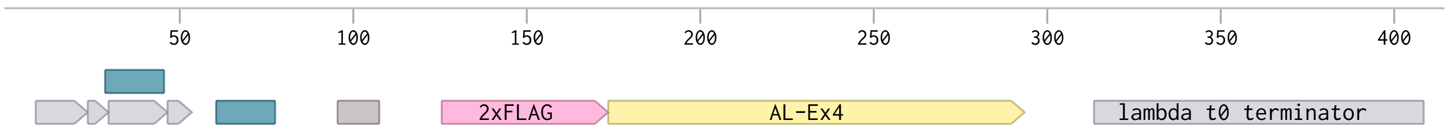

pKPY-AL-Ex4 (partial sequence) (415 bp)

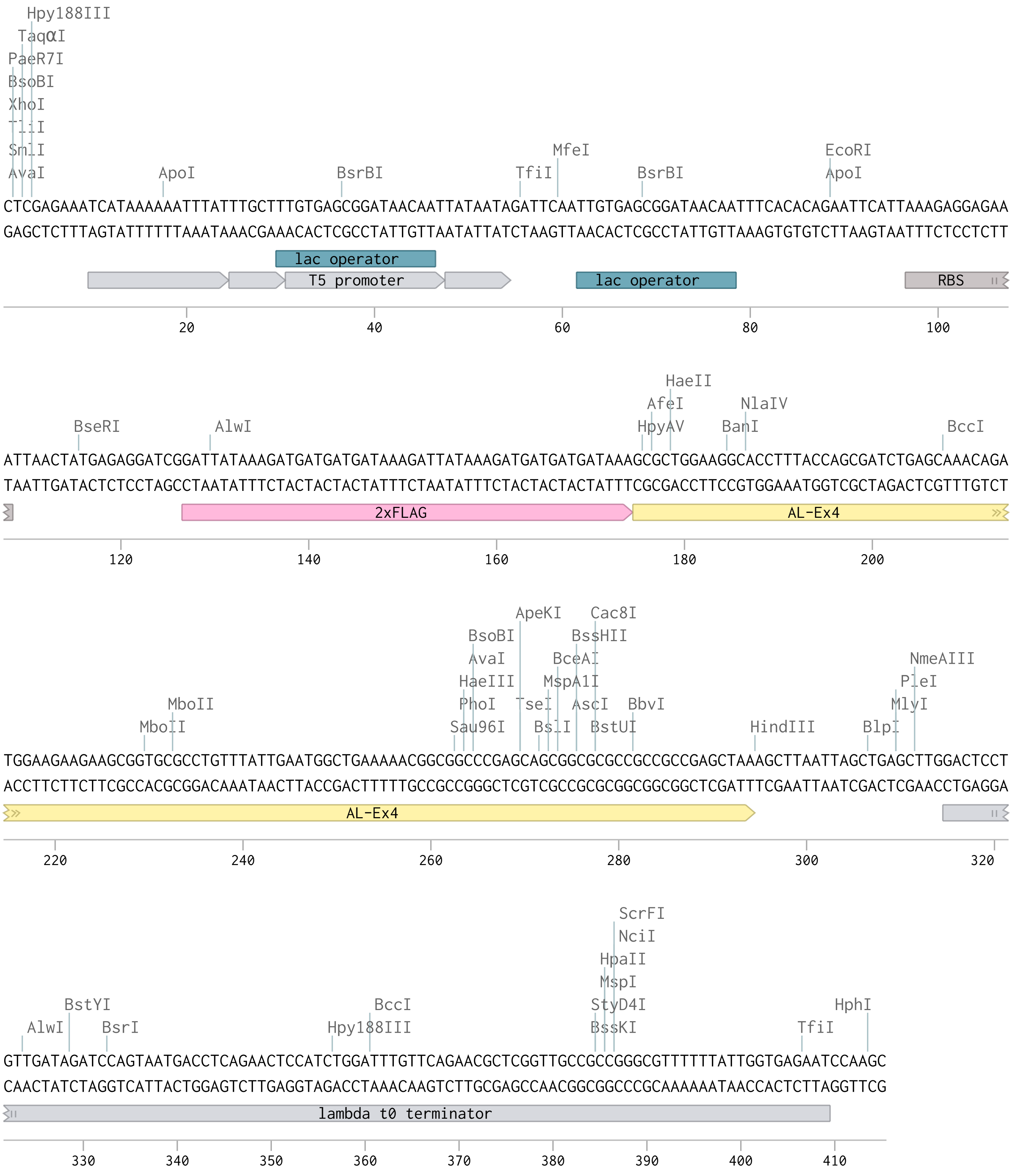

pKPY-TG-Ex4 (partial sequence) (415 bp)

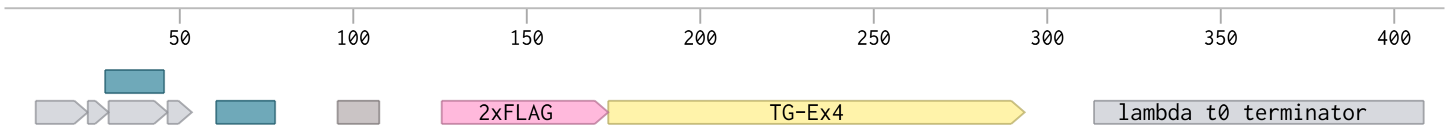

2019/2/18 14:49:40

pKPY-TG-Ex4 (partial sequence) (415 bp)

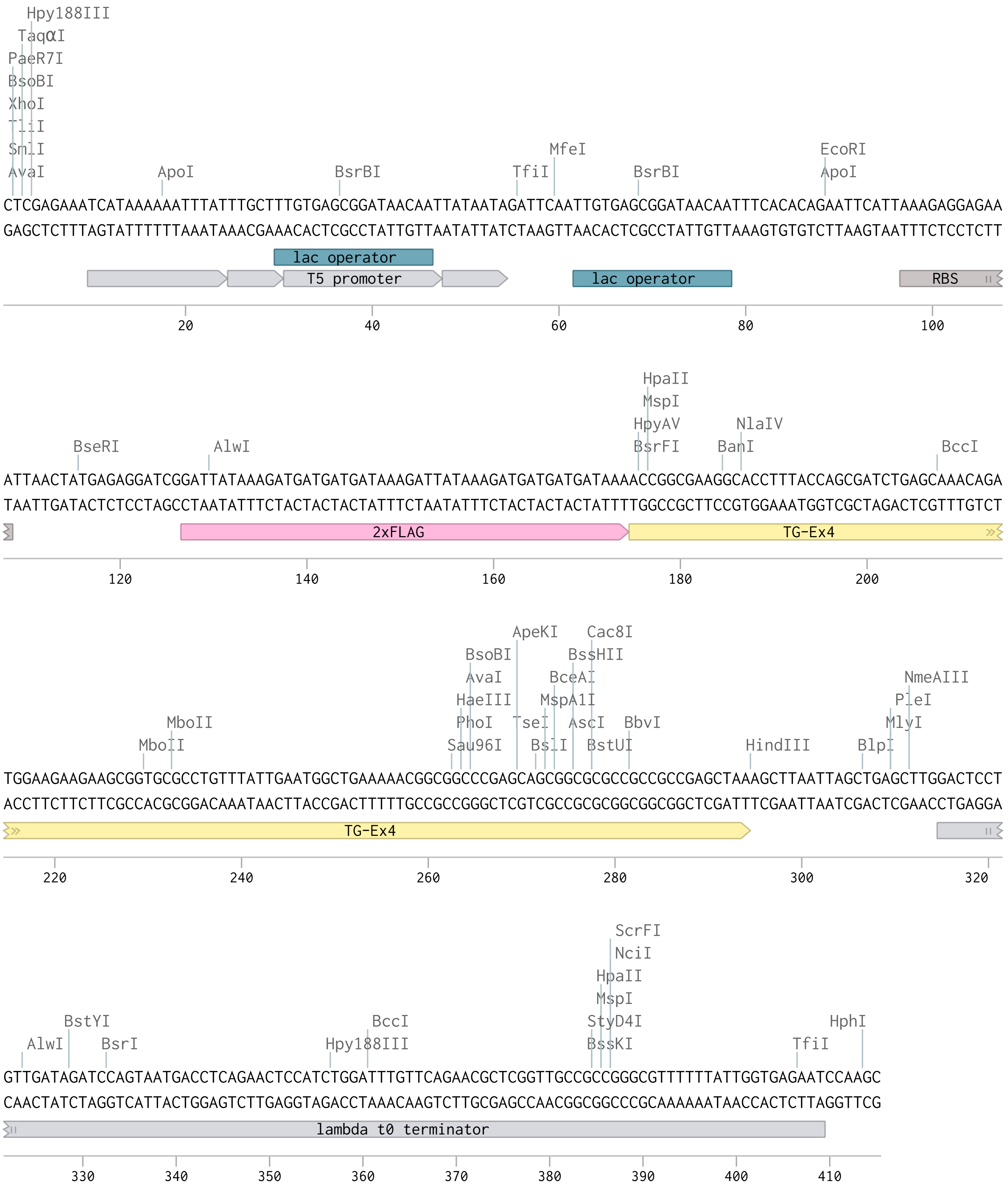

pKPY-SC-Ex4 (partial sequence) (415 bp)

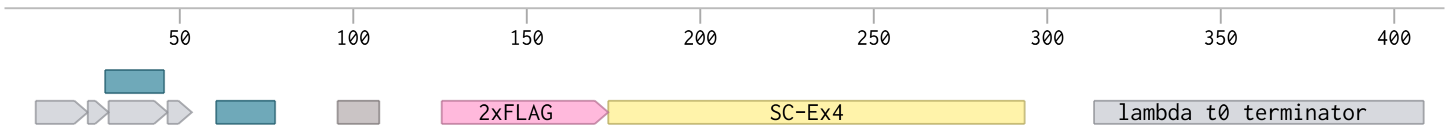

2019/2/18 14:44:13

pKPY-SC-Ex4 (partial sequence) (415 bp)

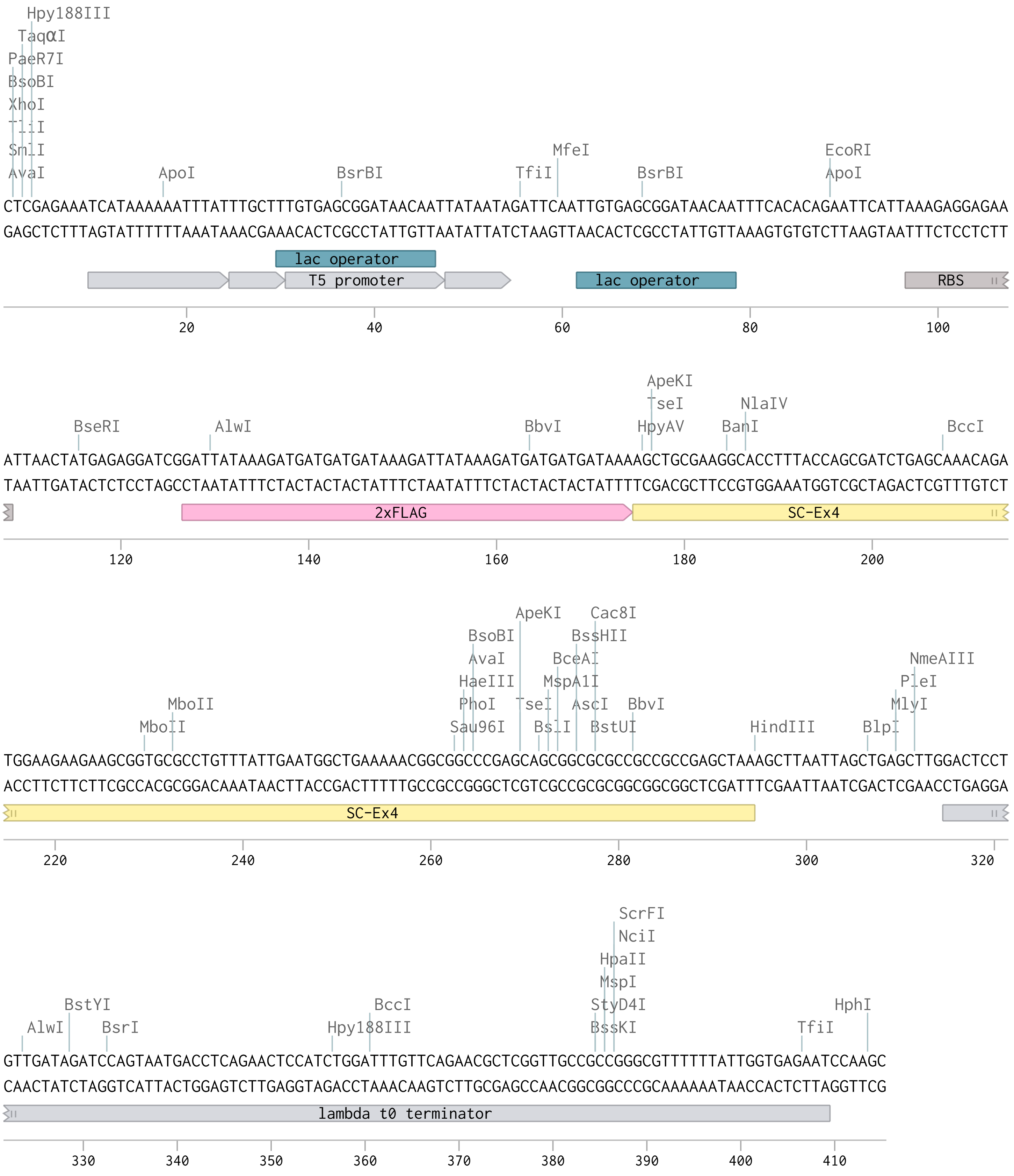

Supplement: Supplementary file 3 — Supplementary Information 3 [file 41598_2019_47388_MOESM3_ESM.pdf]
